# Supplementary material for: A human monoclonal antibody bivalently binding two different epitopes in streptococcal M protein mediates immune function
Source: EMBO Mol Med. 2022 Dec 12;15(2):e16208. doi: 10.15252/emmm.202216208 (PMC9906385; doi:10.15252/emmm.202216208)
Supplement: Supplementary file 2 — Expanded View Figures PDF [file EMMM-15-e16208-s006.pdf]

# Expanded View Figures

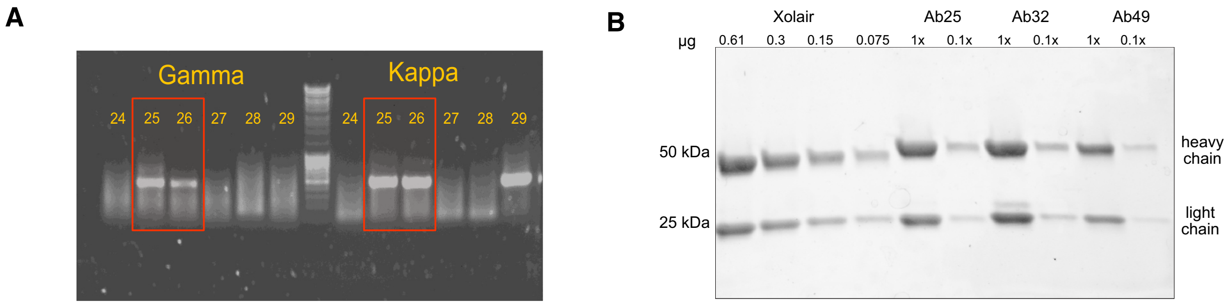


**Figure EV1. Single cell RT-PCR and antibody quality control.**

A RT-PCR was performed to amplify the VDJ and VJ regions coding for heavy and light variable chains, respectively. Two successful PCR examples are Ab25 and 26, shown in the red boxes with both heavy and light chains yielding amplicons.

B SDS-PAGE (4–20% gradient gel) analysis of Ab25, 32, and 49 compared to a serial dilution of Xolair. The gel was stained with Coomassie blue, destained, and imaged.

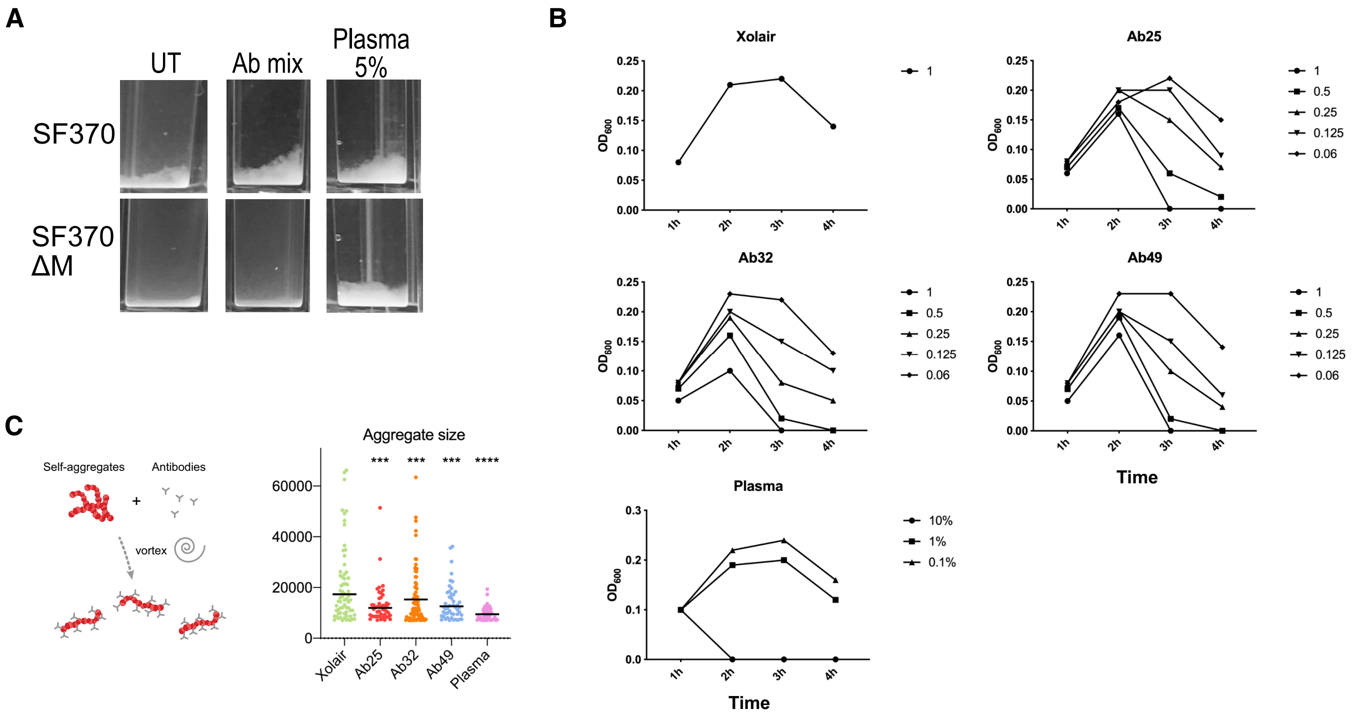


**Figure EV2. Agglutination and aggregate dissolution data.**

A SF370 overnight cultures were diluted 1:5 into THY and incubated with our antibodies (100 µg/ml) for 3 h in plastic cuvettes at 37°C before measuring their OD600. The agglutination effects of the antibody mix (Ab25, 32 and 49 at 100 µg/ml each) were compared on WT and ΔM bacteria. Donor plasma was used as a positive control.

B Dose–response experiment of antibody-mediated agglutination of bacteria, performed as in a with indicated antibody concentrations (µg/ml).

C Bacteria treated with individual antibodies were left standing for 3 h at 37°C before being vortexed. Aggregate size was quantified and tested using one-way ANOVA with multiple comparisons. The data in all the panels represents the results seen in at least three independent experiments. * denotes $P \leq 0.05$, ** for $P \leq 0.01$, *** for $P \leq 0.001$ and **** for $P \leq 0.0001$.

 © 2022 The Authors

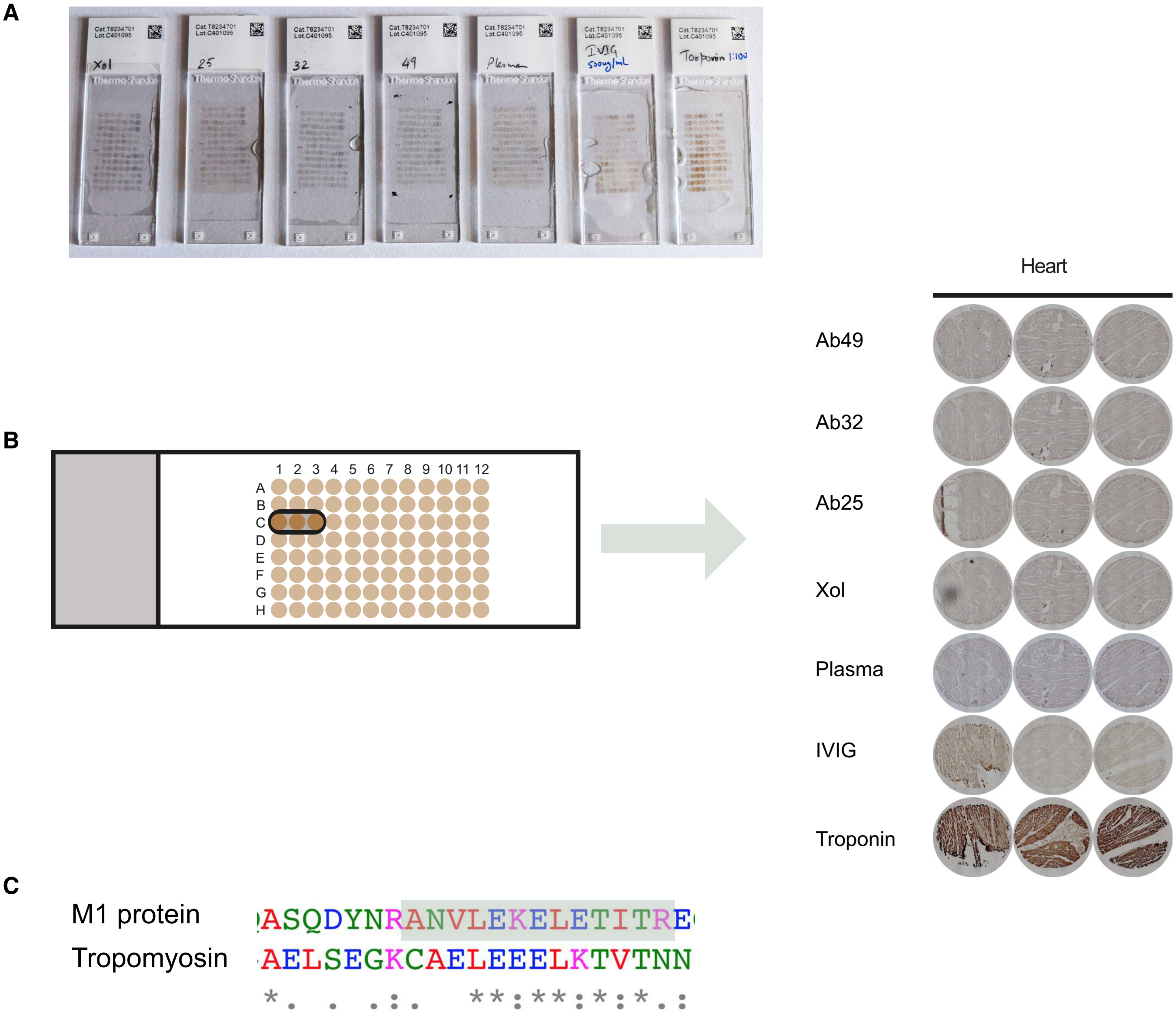


**Figure EV3. Tissue microarray analysis for anti-M antibodies.**

A Tissue microarray stained with 10 μg/ml of Xolair, Ab25, 32, and 49, or 2.5% plasma, 500 μg/ml IVIG or 1:100 anti-Troponin antibody. Each spot is a sampling from different tissue. Tissue sections are 6 μm thick, 1.5 mm wide, and were mounted on positively charged glass slides. None of the tissues showed reactivity with the monoclonal samples, except the positive control anti-Troponin, which was reactive with cardiac tissue, as shown. Out of the 30 tissue types present on the slides, the cardiac samples would otherwise be the tissue type that had the largest risk of reactivity due to the M-protein mimicry with heart tissue.

B Zoomed in images of tissue spots representing cardiac tissue.

C Sequence alignment between M1 protein and tropomyosin. The highlighted area indicates identified the cross-linked peptide (closest to the interaction site) between Ab25 and Ab49.

© 2022 The Authors

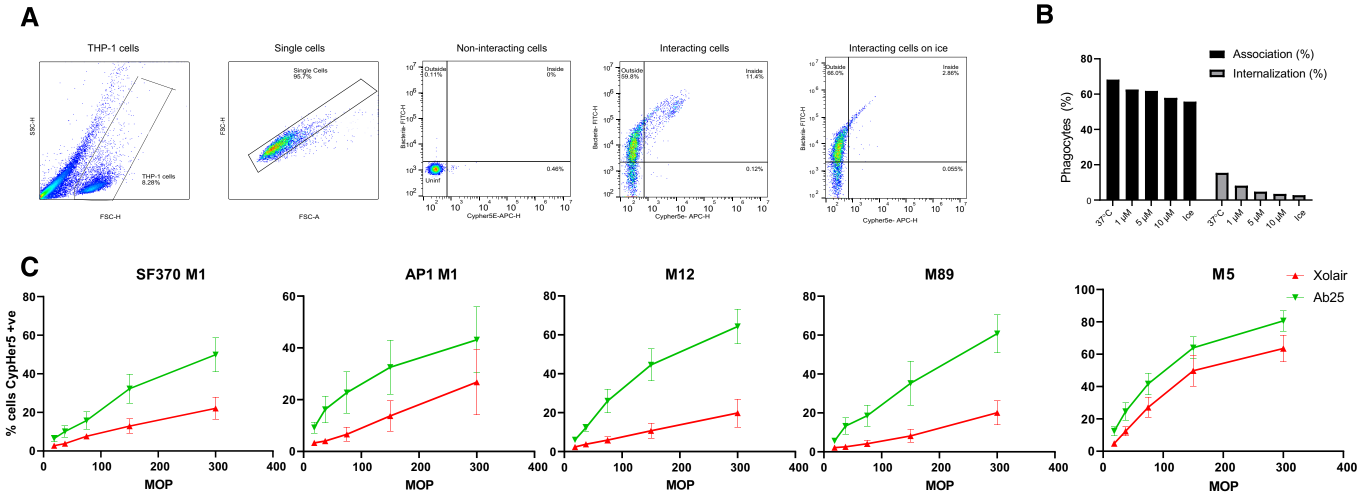


**Figure EV4. Cross-strain phagocytosis of Ab25, controls for phagocytosis, and gating strategy for phagocytosis experiments.**

A From left to right: THP-1 cells are gated based on FSC and SSC. Single cells were selected based on FSC—area and height parameters. Noninteracting cells that have no FITC (bacteria) or CypHer5E (internalized bacteria) signal. Interacting cells associated with bacteria show a shift to the upper left quadrant (outside), whereas THP-1 cells with internalized bacteria shift to the upper right quadrant due to acquisition of the APC signal. THP-1 cells inoculated with bacteria were kept on ice to reduce the phagocytosis. This is visible as a reduction in the number of events in the upper right quadrant.

B Fluorescent *E. coli* bacteria previously opsonized with 1 mg/ml of IVIG beads were incubated with THP-1 cells as in (A) at an MOP 100. In addition to the experiment at 37°C, interactions were allowed to occur on ice or with 1,5, or 10 µM Cytochalasin D. The cells were then analyzed by flow cytometry and the % of cells which had associated with bacteria (internalized or attached) or internalized bacteria were quantified.

C 10 µg/ml of Xolair or Ab25 were used to study phagocytic enhancement across different M serotypes. GAS with serotypes M1 (AP1), M12, M89, and M5 were compared in addition to the M1 SF370 strain. The effect of the antibody treatment on internalization rate was measured using flow cytometry. The error bars represent the standard error of the mean (SEM). The data is from three independent experiments.


© 2022 The Authors

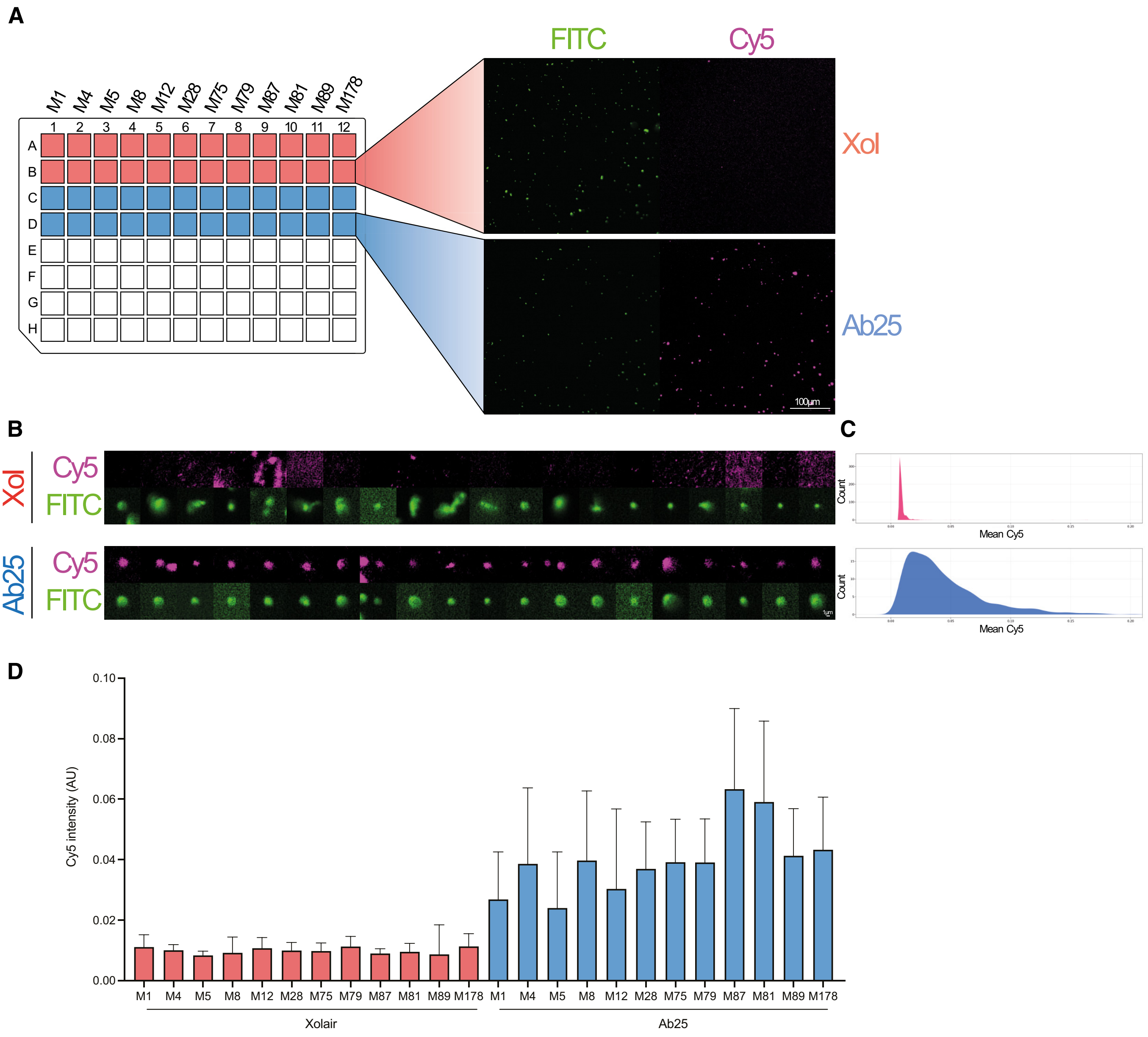


**Figure EV5. Cross-strain immunofluorescence.**

Heat-killed bacteria were stained with Oregon Green (FITC) and incubated with 10 µg/ml Xolair or Ab25, followed by fluorescent secondary antibody detection (Cy5).

A Images were acquired on a wide-field epifluorescence microscope using a 20× objective (NA = 0.75). The acquisition of images was automated using Nikon JOBs by scanning through a 96-well plate acquiring two images/sample. FITC (bacteria) was segmented by a background segmentation algorithm (> median + 5 std of background signal).

B, C Individual bacteria were labeled, and the overlapping signal in Cy5 was measured. Contrast adjustment is set to the same for all shown images. The images shown are from randomly selected bacteria corresponding to the median data population. As the bacteria were chosen by the software without human intervention, some events could be mildly out-of-focus, explaining the blurry appearance.

D Data is from two separate experiments and with a total of ~ 18,000 bacteria analyzed.

© 2022 The Authors
