# Supplementary material for: A human monoclonal antibody bivalently binding two different epitopes in streptococcal M protein mediates immune function
Source: EMBO Mol Med. 2022 Dec 12;15(2):e16208. doi: 10.15252/emmm.202216208 (PMC9906385; doi:10.15252/emmm.202216208)
Supplement: Supplementary file 3 — Table EV1 [file EMMM-15-e16208-s004.pdf]

Cross-link no.	Peptide1	Peptide2	Protein1	Protein2	Cross-link distance (Å)	Spectrum no. in Fig. S4
1	**K**ALELAIDQASQDYNR	DNS**K**NTLYLQMNSLR	M1-B2 repeat	AB25-heavy	21.7	A
2	**K**ALELAIDQASQDYNR	SYPH**K**R	M1-B2 repeat	AB25-heavy	35.4	B
3	ANVLE**K**ELETITR	DNS**K**NTLYLQMNSLR	M1-B3-S domain	AB25-heavy	12.7	C
4	QVE**K**DLANLTAELDK	EA**K**VQWK	M1-C1-C2 repeats	AB25-light	22.5	D
5	N**K**YYADSVK	DNS**K**NTLYLQMNSLR	AB25-heavy	AB25-heavy	39.3	E
6	GPSVFPLAPSS**K**STSGGTAALGCLVK	VEP**K**SCDK	AB25-heavy	AB25-heavy	11.3	F
7	GPSVFPLAPSS**K**STSGGTAALGCLVK	H**K**VYACEVTHQGLSSPVTK	AB25-heavy	AB25-light	24.9	G
8	GPSVFPLAPSS**K**STSGGTAALGCLVK	VYACEVTHQGLSSPVT**K**SFNR	AB25-heavy	AB25-light	11.1	H
9	GPSVFPLAPSS**K**STSGGTAALGCLVK	VDNALQSGNSQESVTEQDS**K**DSTYSLSSTLTLSK	AB25-heavy	AB25-light	18.0	I
10	DNS**K**NTLYLQMNSLR	EA**K**VQWK	AB25-heavy	AB25-light	54.6	J
11	ANVLE**K**ELETITR	DNS**K**NTLYLQMNSLR	M1-B3-S domain	AB49-heavy	30.5	K

Table EV 1. Identified cross-linked peptides between M1, Ab25 and Ab49. The cross-link number is indicated. For peptides 1 and 2, the cross-linked lysine residues are indicated in bold. Protein 1 and 2 indicate the parent proteins of the respective peptides. The cross-linked lysine residue distance is for each cross-link is given, and a reference to a corresponding spectrum in Figure EV 7.
