# Supplementary material for: A human monoclonal antibody bivalently binding two different epitopes in streptococcal M protein mediates immune function
Source: EMBO Mol Med. 2022 Dec 12;15(2):e16208. doi: 10.15252/emmm.202216208 (PMC9906385; doi:10.15252/emmm.202216208)
Supplement: Supplementary file 4 — Table EV2 [file EMMM-15-e16208-s002.pdf]

emm type	emm pattern type (1)	emm cluster (2)	Upper epitope KALELAIDQASQDYNRANVLEKELETITR	Lower epitope QVEKDLANLTAELDK
emm79	E	E3	No defined repeat	C repeat
emm4	E	E1	No defined repeat	C repeat
emm81	D	E6	No defined repeat	C repeat
emm75	E	E6	No defined repeat	C repeat
emm89	E	E4	No defined repeat	C repeat
emm8	E	E4	No defined repeat	C repeat
emm87	E	E3	No defined repeat	C repeat
emm28	E	E4	No defined repeat	C repeat
emm1	A-C	A-C3	B2-B3 repeat	C repeat
emm179	n.a.	Y (D)	No defined repeat	C repeat
emm104	E	E2	No defined repeat	C repeat
emm12	A-C	A-C4	B2-B3 repeat	C repeat
emm5	A-C	Y (A-C)	B2-B3 repeat	C repeat

Table EV 2. Emm type analysis. The M protein strains are listed according to Ab25 binding strength as reported in Fig. 1F. The emm pattern type, and emm cluster according to published reports by McMillan et al, and Sanderson-Smith et al. The majority belong to different E pattern type strains. All emm types have C repeats where the lower binding site of Ab25 is, whereas in most cases there are no defined repeats for the upper binding epitope. The *emm1* strain was used for the cross-linking experiment where the upper binding epitope (KALELAIDQASQDYNRANVLEKELETITR) of Ab25 was determined. In emm1, this epitope is placed in B2-B3 region. Ab49 also shares parts of this epitope (ANVLEKELETITR).

1. McMillan DJ, Drèze PA, Vu T, Bessen DE, Guglielmini J, Steer AC, et al. Updated model of group A *Streptococcus* M proteins based on a comprehensive worldwide study. Clin Microbiol Infect. 2013 May;19(5):E222-9.

2. Sanderson-Smith M, De Oliveira DMP, Guglielmini J, McMillan DJ, Vu T, Holien JK, et al. A systematic and functional classification of Streptococcus pyogenes that serves as a new tool for molecular typing and vaccine development. J Infect Dis. 2014 Oct 15;210(8):1325–38.
