# Supplementary material for: A human monoclonal antibody bivalently binding two different epitopes in streptococcal M protein mediates immune function
Source: EMBO Mol Med. 2022 Dec 12;15(2):e16208. doi: 10.15252/emmm.202216208 (PMC9906385; doi:10.15252/emmm.202216208)
Supplement: Supplementary file 5 — PDF+ [file EMMM-15-e16208-s001.pdf]

Article

TRANSPARENT PROCESS OPEN ACCESS

EMBO Molecular Medicine

A human monoclonal antibody bivalently binding two different epitopes in streptococcal M protein mediates immune function

Wael Bahnan[1], Lotta Happonen[1], Hamed Khakzad[2,3,†], Vibha Kumra Ahnlide[1], Therese de Neergaard[1], Sebastian Wrighton[1], Oscar André[1], Eleni Bratanis[1], Di Tang[1], Thomas Hellmark[4], Lars Björck[1], Oonagh Shannon[1], Lars Malmström[1], Johan Malmström[1] & Pontus Nordenfelt[1,*]

Abstract

Group A streptococci have evolved multiple strategies to evade human antibodies, making it challenging to create effective vaccines or antibody treatments. Here, we have generated antibodies derived from the memory B cells of an individual who had successfully cleared a group A streptococcal infection. The antibodies bind with high affinity in the central region of the surface-bound M protein. Such antibodies are typically non-opsonic. However, one antibody could effectively promote vital immune functions, including phagocytosis and *in vivo* protection. Remarkably, this antibody primarily interacts through a bivalent dual-Fab cis mode, where the Fabs bind to two distinct epitopes in the M protein. The dual-Fab cis-binding phenomenon is conserved across different groups of M types. In contrast, other antibodies binding with normal single-Fab mode to the same region cannot bypass the M protein's virulent effects. A broadly binding, protective monoclonal antibody could be a candidate for anti-streptococcal therapy. Our findings highlight the concept of dual-Fab cis binding as a means to access conserved, and normally non-opsonic regions, regions for protective antibody targeting.

Keywords dual-Fab; immune function; monoclonals; Streptococcus; therapeutics

Subject Categories Immunology; Microbiology, Virology & Host Pathogen Interaction

DOI 10.15252/emmm.202216208 | Received 22 April 2022 | Revised 14 November 2022 | Accepted 16 November 2022 | Published online 12 December 2022

EMBO Mol Med (2023) 15: e16208

Introduction

Antibodies are essential components of the immune system used to recognize and neutralize external intruders such as pathogenic bacteria. They are produced by B cells after their B cell receptor reacts with a specific antigen in the lymphoid tissue. B cell maturation and antibody responses have evolved to allow for an extraordinary variety enabling the binding to most foreign antigens. V(D)J recombination events, as well as somatic hypermutation, give rise to a vast repertoire of antibody variable domains (Pascual *et al*, 1994; Wilson *et al*, 1998). B cell activation, clonal expansion, maturation, and class switching result in the generation of IgG antibodies that offer long-term protection against infectious agents (Kurosaki *et al*, 2015).

An IgG antibody is a Y-shaped molecule composed of two identical Fab domains and one Fc domain, where the unique binding specificity is mediated via the Fab interaction. IgG typically binds the antigen with either one of the two Fabs. The two Fabs can bind to two copies of the same antigen to increase binding strength through avidity, a process that is dependent on antigen density and organization (Klein & Bjorkman, 2010). We designate the latter form of binding as dual-Fab trans binding. When bound to their target, IgG molecules carry out effector functions by triggering clustering of Fc receptors on immune cells (Woof & Burton, 2004). This induces cell signaling and leads to a variety of downstream effects such as phagocytosis, immune recognition, and activation (Lu *et al*, 2018).

Group A streptococcus (GAS) is a common human pathogen causing significant morbidity and mortality in the human population. GAS is an important causative agent of mild as well as severe invasive infections (Mitchell, 2003; Carapetis *et al*, 2005). The bacterium has evolved an extensive array of measures to

1 Division of Infection Medicine, Department of Clinical Sciences Lund, Faculty of Medicine, Lund University, Lund, Sweden
2 Equipe Signalisation Calcique et Infections Microbiennes, École Normale Supérieure Paris-Saclay, Gif-sur-Yvette, France
3 Institut National de la Santé et de la Recherche Médicale (INSERM) U1282, Gif-sur-Yvette, France
4 Department of Clinical Sciences Lund, Division of Nephrology, Lund University, Lund, Sweden
*Corresponding author. Tel: +46 46 222 85 71; E-mail: pontus.nordenfelt@med.lu.se
†Present address: Université de Lorraine, Inria, LORIA, Nancy, France

© 2022 The Authors. Published under the terms of the CC BY 4.0 license.

counteract the human immune response (Okumura & Nizet, 2014), including resistance to phagocytosis (Carlsson *et al*, 2003; Staali *et al*, 2006), and several immunoglobulin-targeting mechanisms (IdeS; von Pawel-Rammingen *et al*, 2002, EndoS; Collin & Olsén, 2001, protein M/H; Åkesson *et al*, 1994). The streptococcal M protein, a virulence determinant, has a long coiled-coil structure with different regions (A, B, S, C, and D). These regions are typically associated with distinct protein interactions and bind many components of the humoral immune response (Ghosh, 2018; Happonen *et al*, 2019), such as C4BP and factor H. Additionally, M protein forms complexes with fibrinogen (Macheboeuf *et al*, 2011) that can induce vascular leakage (Herwald *et al*, 2004) and contributes to phagocytosis resistance (Weineisen *et al*, 2004; Carlsson *et al*, 2005). M protein can specifically reduce phagocytosis by reversing the orientation of IgG by capturing IgG Fc domains (Åkesson *et al*, 1994; Nordenfelt *et al*, 2012). These pathogenic mechanisms deprive the immune system of crucial defenses, allowing GAS to disseminate within a host and across the population. Additionally, the M protein has been implicated in autoimmune sequelae to streptococcal infections. Post-streptococcal sequelae such as rheumatic heart disease and rheumatic fever are due to its molecular mimicry to cardiac tissues (Cunningham *et al*, 1989), further contributing to the pathogenicity of this bacterium.

Although GAS infections generate a humoral immune response, repeated exposures seem to be required to generate protective memory B cell immunity (Pandey *et al*, 2016). There are few candidates for antibacterial monoclonal antibody therapy in general (Motley *et al*, 2019), and none available for GAS. Most of the effort has been allocated to developing vaccines against GAS (Dale & Walker, 2020). The prime immunizing antigen is M protein, particularly M protein-based peptides (Azuar *et al*, 2019). Yet, no effective vaccine against GAS has been approved to date. It is unclear what makes it so challenging to generate immunity against the bacterium. Potentially the formation of antibody subsets could be suppressed by immunodominant regions (Lindahl, 2020) or the presence of cryptic epitopes (Ozberk *et al*, 2018). In severe life-threatening invasive GAS infections, intravenous IgG antibodies (IVIG) from human pooled plasma have been used as therapy, even though reports on their efficacy show contradictory results (Linnér *et al*, 2014; Kadri *et al*, 2017; Madsen *et al*, 2017; Parks *et al*, 2018). In the virus field, it is well established that certain antibodies have strong neutralizing abilities depending on epitope, binding angle, and glycan composition (Burton & Hangartner, 2016). So far, few studies have investigated the properties that constitute a protective antigen-specific antibody response against GAS.

Here, we have generated anti-M protein antibodies derived from a healthy donor who had previously recovered from a GAS infection. When exposed to GAS and M protein, the antibodies bind and exert various effects. We identify a new type of interaction where the two identical Fabs of one of the monoclonal IgG antibodies simultaneously bind to two distinct epitopes. We designate this form of bivalent binding as dual-Fab cis binding. Importantly, this broadly binding antibody efficiently promoted all types of studied protective immune functions, including bacterial agglutination, NF-κB activation, phagocytosis, and *in vivo* protection.

Results

Antigen baiting allows the development of human single-cell derived M-specific antibodies

To understand what constitutes a protective antibody against GAS infection, we generated functional human antibodies and analyzed their effects on virulence. We chose M protein as a target antigen, with a donor that had successfully cleared a streptococcal infection as a source of M protein-specific antibodies. To identify human antibodies with specificity toward streptococcal M protein, we isolated $CD19^+$ $CD3^-$ IgG^+ M-protein$^+$ B cells by baiting donor B cells with fluorescently conjugated M1 protein (Fig 1A). The IgG^+ B cells that did not react with the M protein could have specificities to any antigen, including other streptococcal antigens. Still, the focus of this work was on those B cells that were strongly reactive to M protein. The M protein we used for baiting is derived from an M1 serotype strain. We opted to use an M1 protein without knowing which serotype infected the original donor to increase the likelihood of identifying cross-strain reactive antibodies. Cloning RT-PCR of the variable regions of the heavy and light chains yielded 10 antibody pairs (Fig EV1A). SDS-PAGE and mass spectrometry analysis of the antibodies expressed in HEK293 cells showed correct expression of the intact antibodies (Fig EV1B). Four antibodies showed clear reactivity to surface-bound M1 protein on GAS (strain SF370), the most common M protein among GAS isolates (Fig 1B). Further experiments with three selected antibodies using a ΔM SF370 mutant strain lacking the M1 protein demonstrated that binding of Ab25, 32, and 49 to the streptococcal surface was M protein dependent (Fig 1C). We could confirm that all three antibodies could be found in the donor serum through mass spectrometric analysis of proteotypic peptides.

We measured the antibody-binding affinities to the surface of SF370 with either intact antibodies or $F(ab')_2$ fragments (Fig 1D), the latter to avoid contribution from M1's binding to IgGFc (Åkesson *et al*, 1994). Intact Xolair (Omalizumab, anti-IgE) showed a K_D of 3.2×10^{-6} M, signifying a low binding affinity in concordance with previous reported IgGFc affinity for purified M1 protein (3.4×10^{-6} M; Åkesson *et al*, 1994). $F(ab')_2$ fragments of Ab25, 32, and 49 had considerably higher affinities for M1; 2.3×10^{-9}, 9.1×10^{-9}, and 12.3×10^{-9} M, respectively (Fig 1E). To assess the reactivity of Ab25, 32, and 49 across different GAS M serotypes, we measured the binding of the antibodies to GAS *emm* serotypes 1, 4, 5, 8, 12, 28, 75, 79, 81, 87, 89, 104, and 179. We discovered that Ab25 and Ab49 have similar broad reactivity against these serotypes, with the strongest relative binding to *emm*4 and *emm*79 and the lowest relative binding to *emm*5 (Fig 1F). The cross-reactivity pattern was different for Ab32, including lack of binding to some strains. Whole-genome sequencing of the strains was performed, and genome contigs were assembled. When comparing the DNA sequences of the *emm* genes used in Fig 1F strains, we saw that overall genetic similarity could serve as a good predictor of relative antibody-binding strength (Fig 1G).

Characterization of anti-M antibodies

To characterize the identified anti-M antibodies, we performed a panel of biochemical and immunological assays. We used

 © 2022 The Authors

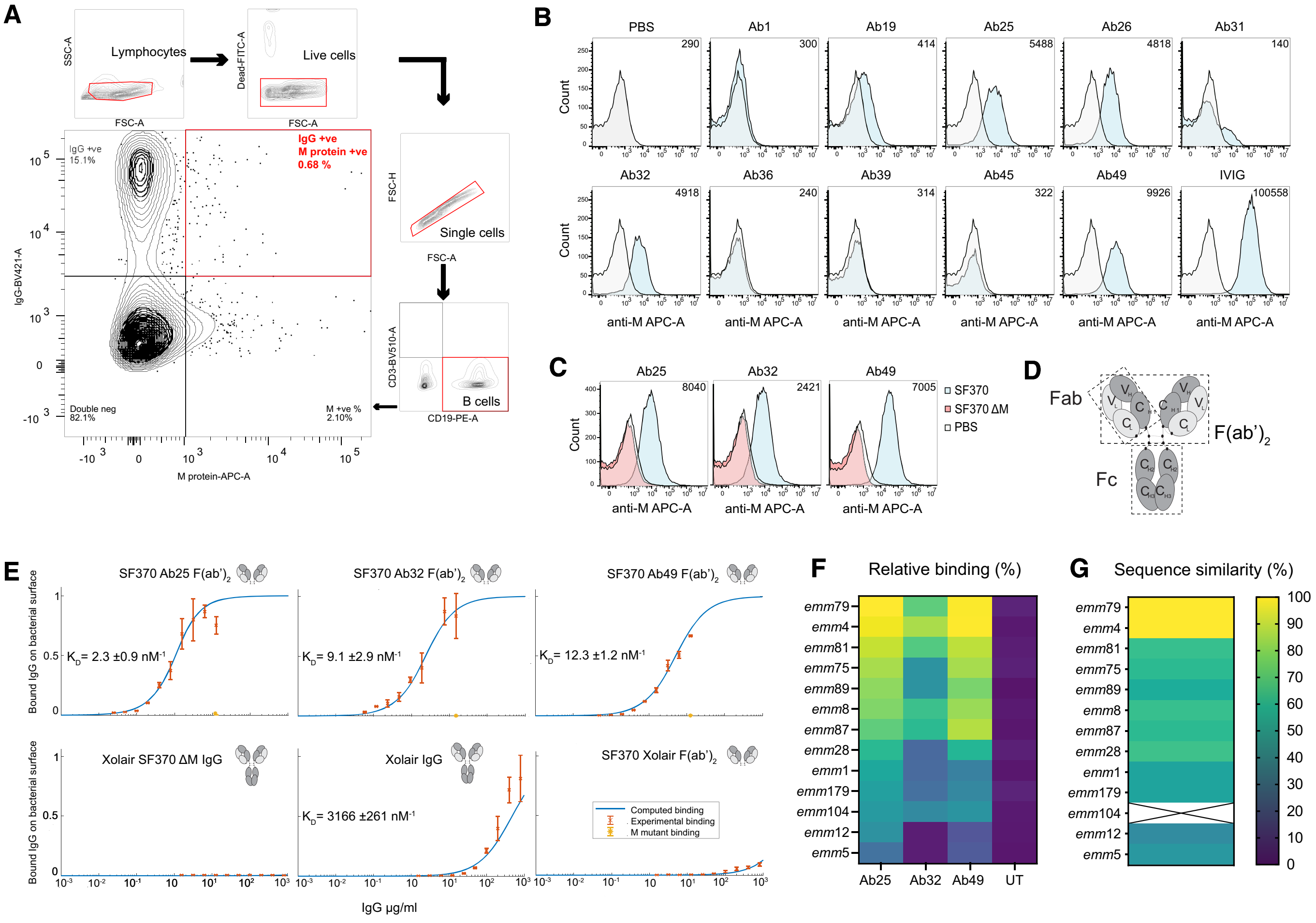

Figure 1. Antigen baiting allows the development of human single cell-derived M-specific antibodies.

A B cells were isolated using Rosettesep B and were FACS sorted into single lymphocytes which were live (Syto9-FITC negative), expressing CD19 (PE), lacking CD3 (BV510), and which were dually positive for IgG and M protein (BV410 and AF647, respectively).

B SF370 (GFP-transformed) bacteria were stained with the shown antibody (F(ab')2 fragments generated with IdeS digestion) and a secondary Fab anti-Fab antibody (conjugated to AF647) was used to generate the signal for flow cytometric analysis. The number in the upper right corner of the flow histograms indicates the median fluorescence intensity.

C The specificity of the successful antibodies was assessed by comparing the staining of WT SF370 and ΔM SF370. The bacteria were stained as in (B) with the aforementioned antibodies and were analyzed by flow cytometry.

D A schematic representation of an IgG antibody.

E Binding curves of antibodies to SF370 give an estimate of affinity to M-protein. The affinity of the specific antibodies is approximately 250–1,500 times higher than that of IgGFc binding to M-protein. Each plot here shows the measured binding (red error bars) of specific antibodies to mid-log grown SF370 or the M mutant as a function of the total antibody concentration, together with a fitted binding curve using the least-squares method (blue curve). The bottom three plots are controls with a non-specific antibody that have been normalized according to the fitting of the bottom left binding curve, making the binding comparable across the three controls. K_D values for the fitting are given in each plot, together with a confidence interval calculated using the Bootstrap method. The binding was measured using flow cytometry. The antibodies (antibody fragments) used are shown on the top-right side of each graph. The plots show no binding of the specific antibodies to the M mutant (orange error bar) at the highest measured total antibody concentration.

F Different M serotypes of GAS were heat-killed and then prepared as in (B) and tested for reactivity against IdeS-cleaved Ab25, 32, 49 or untreated (UT, only secondary). The bacteria were analyzed by flow cytometry, and the data was presented as a heat map. The displayed data is the combined result of three or more independent experiments.

G Whole-genome sequencing was performed on the strains used in (F). The contigs were assembled and the *emm* sequence similarity was compared to *emm79*. The heat map shows the *emm* sequence comparison results. Sequencing of *emm*104 failed and is therefore designated by X.

structured illumination microscopy (SIM) immunofluorescence (IF) to visualize the anti-M-binding pattern on the surface of SF370. Binding to the M protein shows a similar punctate distribution along the surface of the organism with all the monoclonal antibodies, including the Fc-mediated Xolair binding (Fig 2A). IVIG, which contains pooled IgG from thousands of donors, instead stained the whole surface of the bacteria indicating expected polyspecific coverage (Fig 2A). Ab25 showed the best reactivity with M protein using an anti-M ELISA (Fig 2B), whereas Ab32 showed the best binding to M protein in Western blot (WB) experiments (Fig 2C). Taken together, the data from IF, ELISA, and WB indicate that the monoclonals have different modes of

© 2022 The Authors

binding to the M1 protein, most likely due to different M protein conformations under the assay conditions.

Antibody-mediated bacterial agglutination is a well-documented antibody function and has important biological significance, such as enchaining bacteria for effective immune clearance (Mitsi *et al*, 2017; Moor *et al*, 2017). Another well-known interbacterial, GAS-specific phenomenon is the formation of M-dependent bacterial aggregates at the bottom of the growth tube (Frick *et al*, 2000). While it is impossible to grow GAS without self-aggregation, the antibodies greatly enhanced bacterial agglutination. Both the triple antibody cocktail and individual antibodies led to dose-dependent agglutination, as is the case with donor serum from the patient from which the M-reactive B cells were obtained (Figs 2D and EV2A and B). This agglutination was not observed for the ΔM strain, further validating that the antibody-dependent agglutination is an M-specific phenomenon. Serum, however, can agglutinate GAS without binding to the M protein as it contains antibodies targeting non-M surface proteins. Agglutinated bacteria and the typical GAS aggregates could be dissipated by vigorous vortexing in the presence of the anti-M antibodies or plasma (Fig EV2C). GAS agglutination and aggregate dissolution (after vortexing) were most pronounced in Ab25, 49, and lesser with Ab32, while Xolair (with only IgGFc binding) had no effect. This effect has not been seen before and its importance is unknown.

By ligating their antigens and mediating antigen uptake, antibodies also activate macrophages leading to a proinflammatory cytokine production (Bournazos *et al*, 2017). We addressed the antibody-dependent M protein-induced immune activation using THP-1 X-Blue reporter cells, which secrete SEAP (secreted embryonic alkaline phosphatase) as a quantitative indicator of NF-κB activation. We found that M protein alone cannot induce NF-κB signaling. However, combining M protein with Ab25 led to a significant 2.8-fold increase in NF-κB activation compared to M protein with Xolair (Fig 2E). Combining M protein with Ab49 had a modest effect on NF-κB activation (1.6-fold, ns), whereas its combination with Ab32 had no impact (1.2-fold). Combining all three antibodies led to a significant cumulative 3.9-fold increase in NF-κB activation, probably due to the combined amount of Ab25 and 49. Interestingly, we found that IVIG does not elicit the same antibody-mediated NF-κB activation upon THP-1 X-Blue exposure to the IVIG-treated M protein (1.4-fold).

Some anti-M protein antibodies have been known to cross-react with human cardiac tissues. We tested the autoreactivity of our antibodies using tissue microarrays and saw no detectable cross-reactivity with any human tissue (Fig EV3A and B). The only antibody that showed a clear level of autoreactivity with multiple tissue samples (seen as brown color) was the Troponin-specific antibody. Troponin is a cardiac protein, and we used an anti-Troponin antibody to replicate a cardiac-reactive antibody. The monoclonals' combined biochemical and immunological characterization shows that all are specific for M protein, bind to different epitopes, and can induce immunological effects without being autoreactive and that Ab25 has the most potent effect overall.

Anti-M antibody promotes efficient phagocytosis

Phagocytosis is a receptor-mediated process where prey are internalized into phagosomes, followed by their maturation into acidic, hostile compartments (Nordenfelt & Tapper, 2011). To investigate the ability of the antibodies to trigger phagocytosis, we used persistent association-based normalization (de Neergaard *et al*, 2021) to study both the antibodies' ability to increase phagocyte association as well as internalization. We incubated phagocytic THP-1 cells with pH-sensitive CypHer5-stained bacteria (Fig EV4A) at increasing multiplicities of prey (MOP). We used ice and Cytochalasin D as phagocytosis inhibition conditions to assess the baseline of our assays (Fig EV4A and B). We combined Ab25, 32, and 49 to assess their cumulative effect on bacterial association with THP-1 cells. Compared to Xolair, the antibody mix and, to a lesser extent, IVIG modestly increased the overall association of THP-1 cells to the bacteria (Fig 3A). This can be seen as a left shift in the curve, meaning fewer bacteria are required to achieve maximal association. When tested individually and assaying the number of bacteria per cell, only Ab25 showed a statistically significant increase in association of bacteria to phagocytes, indicating that the antibody mix-mediated increase in the association is solely due to Ab25 (Fig 3B). The antibody mixture increased internalization compared to Xolair, and the divergence between the two treatments was increased as a function of MOP (Fig 3C). Upon more detailed examination of individual antibodies, only Ab25 showed a statistically significant increase in internalization (Fig 3D). Dose–response analysis showed that Ab25 is significantly more effective than Xolair in mediating internalization (concentration at which 50% of THP-1 cells have internalized bacteria; EC_{50} 0.8 vs. 40.2 μg/ml; Fig 3E). Expanding on our previous cross-strain reactivity data (Fig 1F), we used Ab25 to set up an imaging-based binding assay (Fig EV5A and B). By utilizing multiple strains, we show that Ab25 has broadly binding potential against GAS compared to Xolair (Fig EV5C and D).

Figure 2. Characterization of anti-M antibodies.

A Structured illumination microscopy (SIM) super-resolution imaging was performed on bacteria stained with IdeS cleaved α-M antibodies and probed with Dylight488-conjugated Fab α-Fab fragments. AF647-conjugated WGA was used as a counter-stain to highlight the bacterial structures. The scale bar represents 5 μm.
B Antibodies were tested for reactivity against the M protein in an ELISA assay where the M protein was immobilized onto the well surface. Xolair and PBS were used as negative controls. The absorbance at 450 nm was measured and plotted as an average of quadruplicate wells.
C Protein lysates from logarithmically grown WT and ΔM SF370 bacteria were run on SDS-PAGE and, after blotting, were probed with the mentioned antibodies at a concentration of 2 μg/ml.
D Time course analysis of the effect of the antibody mix or individual antibodies used at 100 μg/ml each on bacterial agglutination (SF370 GAS and the ΔM) was assessed by OD_{600} measurements. An increase in OD signifies growth and a subsequent drop in OD represents agglutination.
E THP-1-Blue cells were treated with M1 protein (2 μg/ml) with or without anti-M specific antibodies.

Data information: The data shown are from triplicate samples, representing experiments done three times. Error bars represent the SD. Statistical significance was evaluated using a two-way ANOVA with Dunnett's multiple comparison correction. * denotes $P \leq 0.05$, ** for $P \leq 0.01$, and **** for $P \leq 0.0001$.

© 2022 The Authors

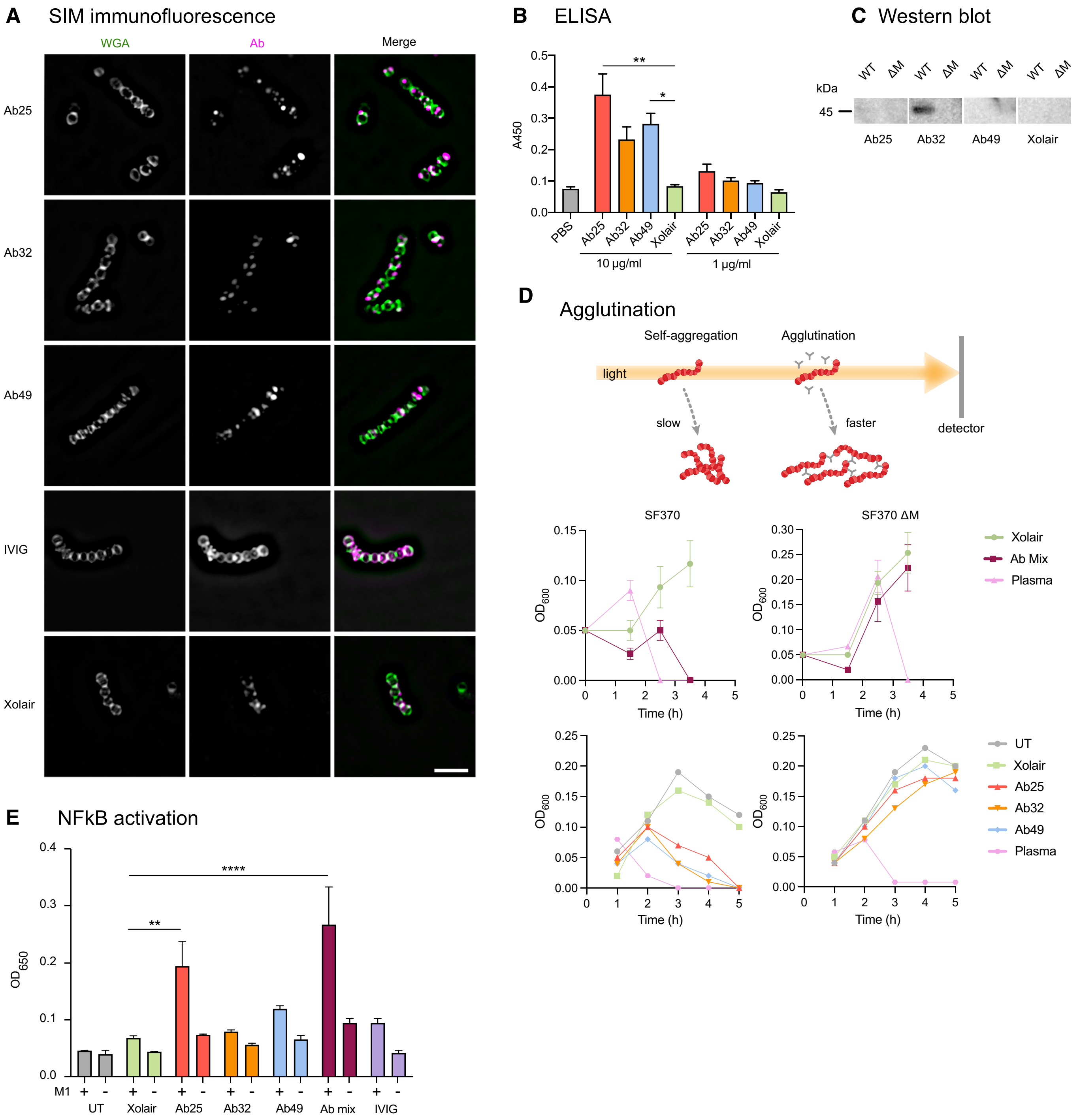

Figure 2.

The phagocytosis data showed that despite strong Fab-mediated binding and induction of other immunological effects by all monoclonals, only one antibody, Ab25, can promote phagocytosis of group A streptococci. We have shown earlier (Fig 1F) that Ab25 possesses broadly reacting potential against different M types. For that reason, we tested its ability to opsonize and mediate the phagocytosis of non-M1 serotypes. Opsonization with Ab25 led to increased phagocytic efficiency of the M1 (SF370 and AP1), M12, and M89 serotype strains, whereas the M5 was efficiently phagocytosed by THP-1 cells regardless of whether a binding opsonin was employed or not (Fig EV4C). We established a microscopy-based (Appendix Fig S1A) whole-blood phagocytosis assay to validate our *in vitro* findings with THP-1 cells. Upon infecting blood with antibody-opsonized live GFP-expressing M1, M5, M12, and M79 strains, we studied the effect of antibody opsonization on the association between the bacteria and the $CD18^{+}$ cells (Fig 3F). The

phagocytosis enhancement was most pronounced in M1 and M12 (Fig 3F and Appendix Fig S1B). This effect was not present in M5, which is in line with our previous data from THP-1 cells (Fig EV4C). The Ab25-mediated opsonization of M79 was donor dependent as one donor showed decreased phagocytosis upon bacterial opsonization, and one donor showed enhancement. Donor variability was also seen with the M1 strain, albeit less pronounced than with M79. We assessed the interaction between the CD18$^+$ cells (neutrophils in this case) with the GFP-expressing Ab25-opsonized bacteria. We saw that a large proportion of the neutrophils had internalized bacteria, and an example is shown in Fig 3G. Overall, the results from opsonization and phagocytosis show that Ab25 can bind to and opsonize multiple M strains and that this leads to internalization by human phagocytes.

Anti-M antibody protects mice from GAS infection

The induction of phagocytosis and NF-κB, as seen with Ab25, are important indicators of immune function. To test the potential protective effects of Ab25 *in vivo*, we used a mouse model of

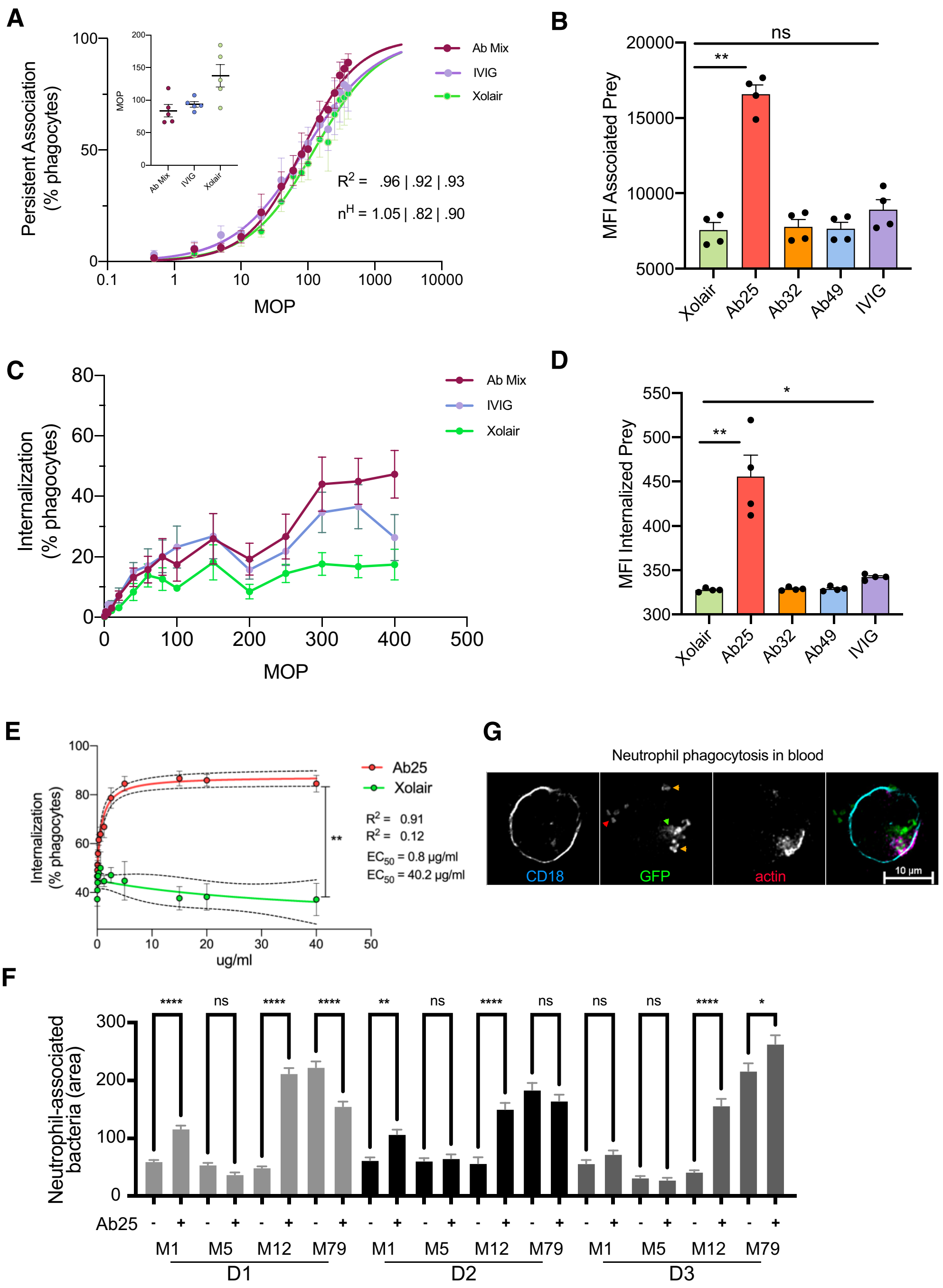

Figure 3.

 © 2022 The Authors

Figure 3. Anti-M antibody promotes efficient phagocytosis.

A THP-1 cells were incubated with increasing MOPs of heat-killed SF370 bacteria (opsonized with 10 μg/ml Xolair, anti-M antibody mix, or IVIG). The THP-1 cells were allowed to associate with and internalize the bacteria for 30 min before flow cytometric analysis. The curves represent the percentage of cells associated with bacteria as a function of the MOP. The inset displays the MOP_{50} for each opsonization condition. (n^H) represents the Hill coefficient.

B The MFI of THP-1 cells in the FITC channel indicates the effect of each antibody on bacteria association to THP-1 cells at MOP 400.

C THP-1 cells were incubated as in (A), but only the percentage of cells with internalized bacteria were plotted for each MOP.

D MFI of THP-1 cells, which had internalized bacteria pre-opsonized with 10 μg/ml individual antibodies, are presented at an MOP of 400 (internalized bacteria channel).

E Heat-killed SF370 opsonized with Ab25 or Xolair at concentrations of (0.017–40 μg/ml) were incubated with THP-1 cells at MOP 300. Phagocytosis was assessed as in (B). The data shown in this fig (A–E) is from the pooled results of three independent experiments.

F Whole blood phagocytosis was performed with samples from three donors (D1, D2, and D3). The blood was diluted and then infected (MOP 10) with live GFP-expressing bacteria (pre-opsonized with Xolair or Ab25 (10 μg/ml)) for 30 min before PFA fixation. The cells were then stained with SiR-Actin (Em:674), and anti-CD18 (BV421, blue) were immobilized on CD29-coated glass plates and imaged with 20× magnification. From each donor, the area of GFP signal associated with at least 440 cell data points was recorded. The data shown represent at least 560 cell events collected per condition. Similar results were seen in two independent experiments (4 donors).

G An example image of Ab25-opsonized M12 bacteria infecting a neutrophil with internalized bacteria (green arrow), extracellular bacteria (red arrow), and active internalization events (yellow arrow, indicated by actin polymerization) is shown. The cell was imaged with 60× magnification.

Data information: Error bars represent the SEM. Statistical significance was assessed using one-way ANOVA with Kruskal–Wallis multiple comparison correction and * denotes $P \leq 0.05$, ** for $P \leq 0.01$, and **** for $P \leq 0.0001$.

subcutaneous infection with GAS. We opted to only test Ab25 in our *in vivo* model since it had the strongest *in vitro* immune activity and we wanted to know whether or not it could be a useful therapeutic molecule. The mice were pretreated with intraperitoneal injections of Ab25 or IVIG. High-dose IVIG has been used in mice models of severe GAS infections (Sriskandan *et al*, 2006) and served as a positive control. Treatment with IVIG or Ab25 reduced the bacterial burden in the spleen, kidney, and liver compared to untreated controls, with Ab25 exhibiting better protection than IVIG (Fig 4A). Ab25 or IVIG treatment also reduced the cytokine mobilization of TNFα, MCP-1, and IL-6 in plasma (Fig 4B). The levels of IFNγ, IL-10, and IL-12p70 were below the level of detection under our experimental conditions. It is interesting to point out that the same Ab25-treated mice which were outliers in organ CFU counts were outliers in all other parameters. Taken together, the agglutination, NF-κB, phagocytosis, and animal experiments show that Ab25 has an immunomodulatory effect, which can protect animals from GAS infection.

Structural epitope characterization reveals bivalent dual-Fab cis-binding mode of interaction

Antibodies that bind via their Fabs with high affinity are typically expected to promote an immune response. However, only Ab25 promoted all the tested immune effector functions. To assess structural differences and their mode of binding, we performed targeted cross-linking coupled to mass spectrometry (TX-MS; Hauri *et al*, 2019) of the M-protein and the antibodies. TX-MS first models quaternary conformations of protein complexes that are, in a second step, confirmed by targeted cross-linking analysis of the most high-scoring models. The high-resolution models of the Fab fragments of Ab25, Ab32, and Ab49 binding to their respective epitopes on M protein (Fig 5) revealed a high degree of structural similarity between Ab25 to Ab49 over the CDR H3 loop (RMSD 1.0 Å). In contrast, the conformation of Ab32 CDR H3 is more divergent (Fig 5B and C). The conservation at the primary amino acid sequence level of Ab25 and Ab49 is less evident (Fig 5A). The subsequent targeted cross-linking analysis identified 10 cross-linked peptides between Ab25 and M1 protein. These cross-links are found between the $F(ab')_2$ and two different regions on the M-protein, indicating that Ab25 has two different binding sites in the B-S-C region (Fig 5D and F, Table EV1, Appendix Fig S2A–J). Structural mimicry between M protein and tropomyosin is well recognized and sequence alignment between the binding sites and tropomyosin shows similarity at one location (Fig EV3C). Our previous tissue microarray data show however that Ab25 and Ab49 are not cross-reactive with human tissues (Fig EV3A and B).

Superimposing the cross-linked distant constraints onto high-resolution docking models shows that Ab25 $F(ab')_2$ can simultaneously bind the two cross-linked epitopes without inducing large conformational changes in the hinge region. This implies that Ab25 is capable of dual-Fab binding in an intramolecular bivalent cis-binding fashion to two distinct, nonidentical epitopes. In contrast, the cross-link analysis of M-Ab49 generated two unique cross-linked peptides confined to only the upper epitope found with Ab25 (Fig 5E, Table EV1, Appendix Fig S2K and L).

The amino acid sequences corresponding to the M proteins obtained from whole-genome sequencing are shown in Appendix Data S1. Amino acid alignment and sequence comparison of the different M proteins are shown in Appendix Data S2. The domains of the M1 protein are shown in Fig 5F, and the two identified epitope peptides for Ab25 and 49 are highlighted. Ab49 only interacts with the upper epitope, whereas Ab25 interacts with both. Based on our genetic sequencing shown before (Fig 1G, Appendix Data S1 and S2), we displayed the *emm* sequence similarity as a percentage of M1 (Fig 5G). In the same panel we also compared the conservation of the Ab25-binding epitopes on the M protein across the strains. We saw that the lower binding site (binding site 2) for Ab25 is relatively conserved across strains, more so than the upper site (binding site 1; Fig 5G). Overall, both sites' conservation when compared to *emm*1 is higher than the general *emm* strain sequence conservation. The *emm* types can be divided into patterns (A–C, D, and E; McMillan *et al*, 2013), belonging to different clades and clusters (Sanderson-Smith *et al*, 2014). This classification shows that the majority of the strains used here belong to the E pattern type (Table EV2), but that *emm*1 belongs to a different clade. Since the strongest Ab25 binders have no clearly conserved regions in the upper binding site, it is likely that the original upper epitope is not found in a B repeat, but that it has cross-reactivity to the B2-B3 repeats in A–C pattern *emm* types. This could be

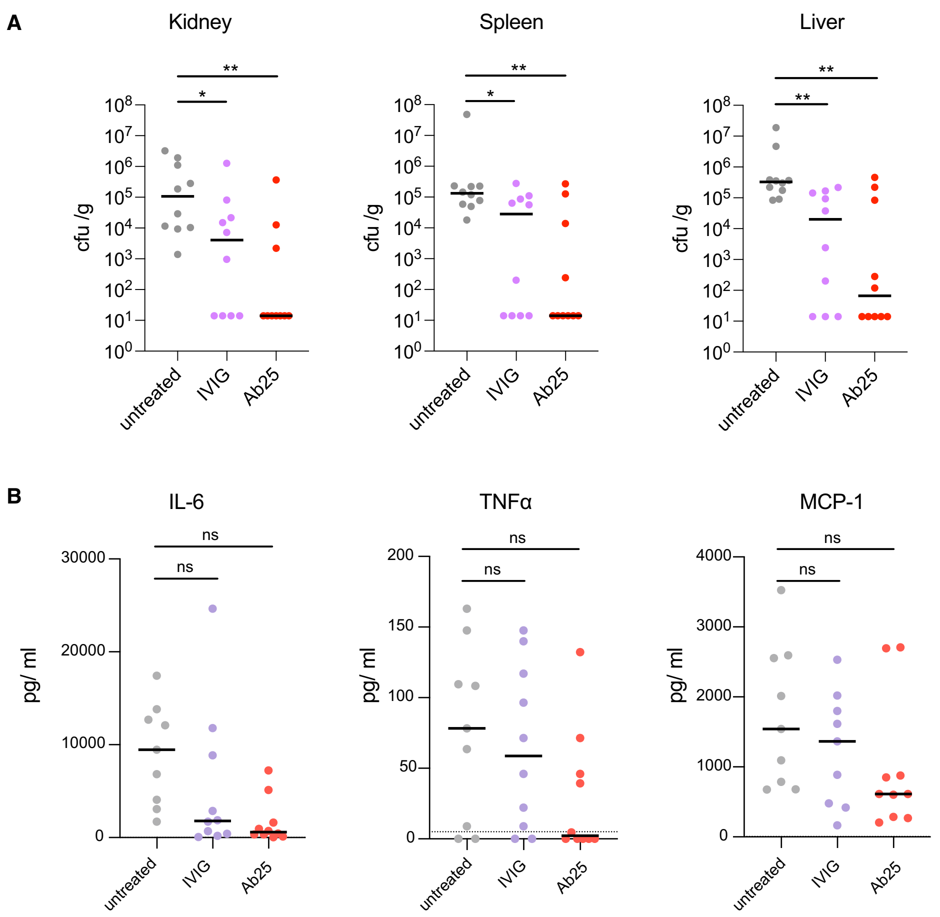

Figure 4. Anti-M antibody protects mice from GAS infection.

C57/BL6 mice were pretreated with 10 mg IVIG or 0.4 mg Ab25 per mouse before subcutaneous infection with 10^6 CFU of GAS (*S. pyogenes* AP1). The animals were sacrificed after 24 h.

A The bacterial burden in spleen, kidney, and liver tissue was measured by colony counts (CFU/gram of tissue).

B Cytokine levels in plasma were assessed using a cytometric bead array.

Data information: The data are pooled from two independent experiments (five mice per condition, per set). Statistical significance was evaluated using one-way ANOVA with Kruskal–Wallis multiple comparison corrections and * denotes $P \leq 0.05$, ** for $P \leq 0.01$.

explained by structurally similar and appropriately charged motifs which allow for the stable dual-Fab cis binding.

To further investigate the differences in the observed binding sites, we used site-localization microscopy (Kumra Ahnlide *et al*, 2022), where the relative distance between the fluorescently labeled cell wall and antibody-binding epitopes was determined by repeated measures of multiple individual bacteria (Fig 5H). The height analysis showed that all monoclonal $F(ab')_2$ fragments bind close to the Fc-binding domain (S) on the M1 protein (compared to Xolair Fc binding), which supports the TX-MS cross-linking-based results.

Dual-Fab cis mode of interaction is required for functional antibody binding

Intriguingly, Ab25 binds across the M1 protein S-region (Fig 5D and F) that has previously only been associated with Fc-mediated binding (Åkesson *et al*, 1994; Nordenfelt *et al*, 2012). Since the antibodies appear to interact with epitopes close to the Fc-binding S domain (as seen in Fig 5D–H), we further investigated whether the antibodies could interfere with Fc binding. The dual-Fab cis binding of Ab25 covers the S domain (colored in orange in Fig 5D) and would obstruct Fc binding, whereas single Fab interactions would lead to minor or no interference with Fc binding. We measured the binding of fluorescent Xolair to SF370 bacteria that had been preincubated with antibody samples (Fig 6A). Both blood plasma from the original B cell donor and Ab25 significantly obstructed Fc binding, whereas IVIG, Ab32, and Ab49 did not. Since Ab49 and Ab25 share one similar epitope, located above the S region, it indicates that binding there alone is not sufficient to break the Fc interaction, and strongly suggests that this is due to a dual-Fab cis-binding quality of Ab25. Our data also indicates that blood from patients convalescing after GAS infection is enriched with dual-Fab-binding antibodies (Fig 6D) compared to that from normal donors, including IVIG.

Dual-Fab cis antibody binding of two identical Fabs to two different epitopes on a single protein is, to our knowledge, a novel, previously undescribed mode of antibody interaction. Dual-Fab cis binding is also associated with an apparent gain in immunological

© 2022 The Authors

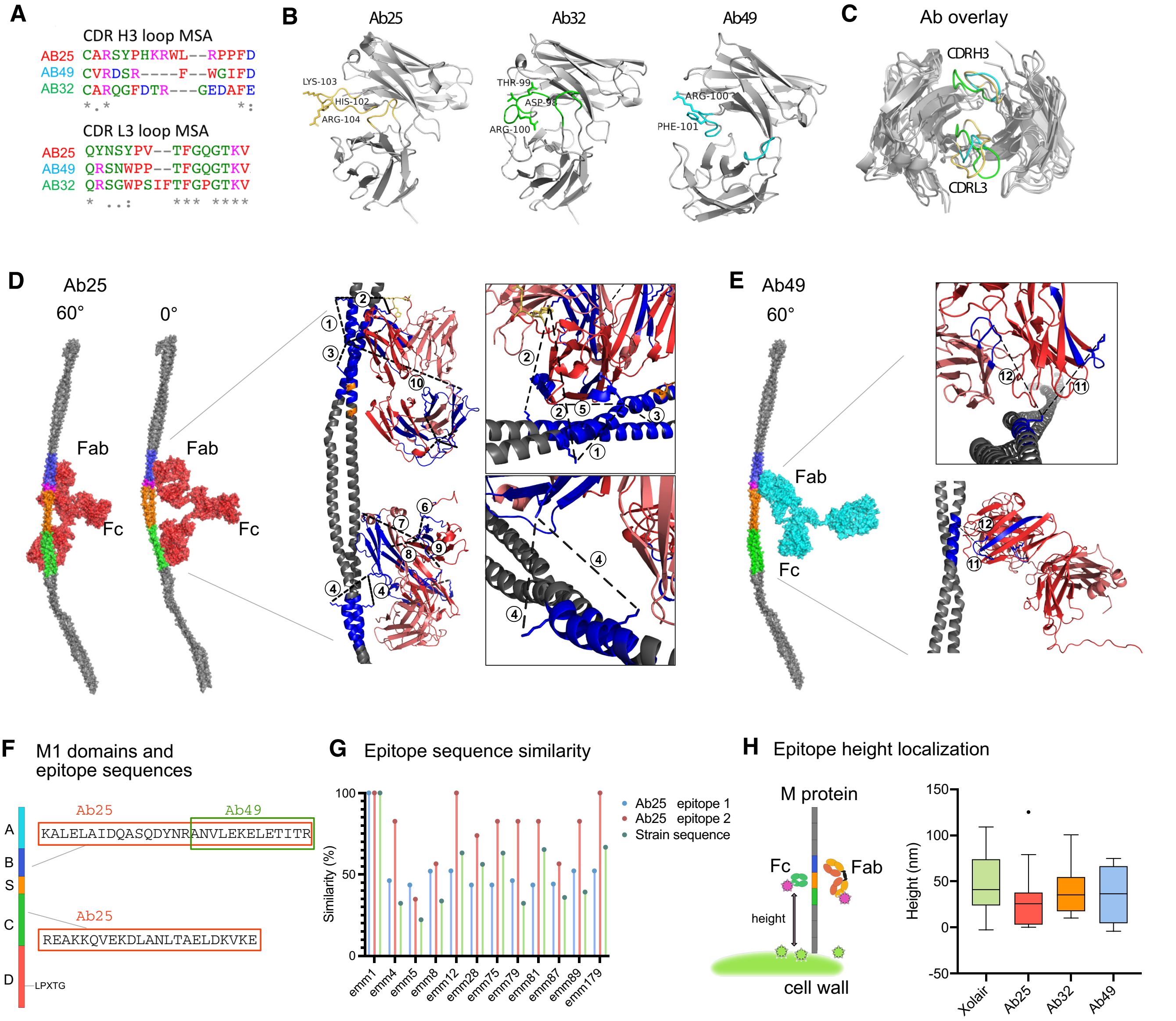

Figure 5. Structural epitope characterization reveals dual-Fab cis-binding mode of interaction.

A Multiple sequence alignment reflects the L3-H3 loop differences on the amino acid sequence level.

B Representations of the Fab fragments of the modeled antibodies Ab25, Ab32, and Ab49. The CDR H3 and L3 loops are indicated (Ab25, yellow; Ab32, green; and Ab45, turquoise).

C Structural alignment of Ab25 (yellow CDR loops), Ab32 (green loops), and Ab49 (turquoise loops).

D, E (D) The M1 interaction sites with Ab25 (red) reveal the binding epitopes. A rotation of 60° along the y-axis has been applied between the views. The contact surface length is 45 Å in the upper binding site (B2–B3-domain) and 18 Å in the lower (C-domain). (E) The M1 interaction site with Ab49 (turquoise) is presented. The length of the contact site for Ab49 (B2–B3 domain) is approximately 16 Å. Insets in (D) and (E) represent a full-length model generated by TX-MS (Hauri *et al*, 2019), showing the Fab-mediated interaction of Ab25 or Ab49 (heavy chain in dark red, light chain in a lighter shade) with the M1 protein (in gray). Cross-linked peptides are indicated in dark blue and cross-links between lysine (K) residues as dashed black lines. The CDR H3 loop is shown in yellow (for Ab25) or red (for Ab49). There are two interaction sites for Ab25 on the M1 protein, the upper one in the B2-B3-domain (inset) supported by four cross-links and the lower one in the C1-domain (inset) by six cross-links. Only one interaction site for Ab49 with M1 exists and is supported by two cross-links.

F Schematic presentation of the M1 protein with the two binding cross-links for Ab25 (binds both) and 49 (binds upper) being displayed.

G Sequence similarity on genetic sequences was performed to compare the two identified epitopes across strains.

H The M protein is associated with the bacterial cell wall through the LPXTG motif in the C-terminal D domain. The Fab epitopes and the Fc-binding region on the M protein are estimated by a fluorescence localization averaging method using structured illumination microscopy (SIM) images (Kumra Ahnlide *et al*, 2022). A relative binding site is determined by resolving the distance between the antibody and a reference channel (WGA, binds cell wall) using the cumulative signals from many images. The results shown here are from four independent experiments with $N = 9, 13, 9, 9$, respectively. Median values are shown, with boxes representing 25th to 75th percentile and whiskers adding a 1.5 interquartile range (Tukey). No significant differences in localization were found using a Kruskal–Wallis multiple comparisons test.

© 2022 The Authors

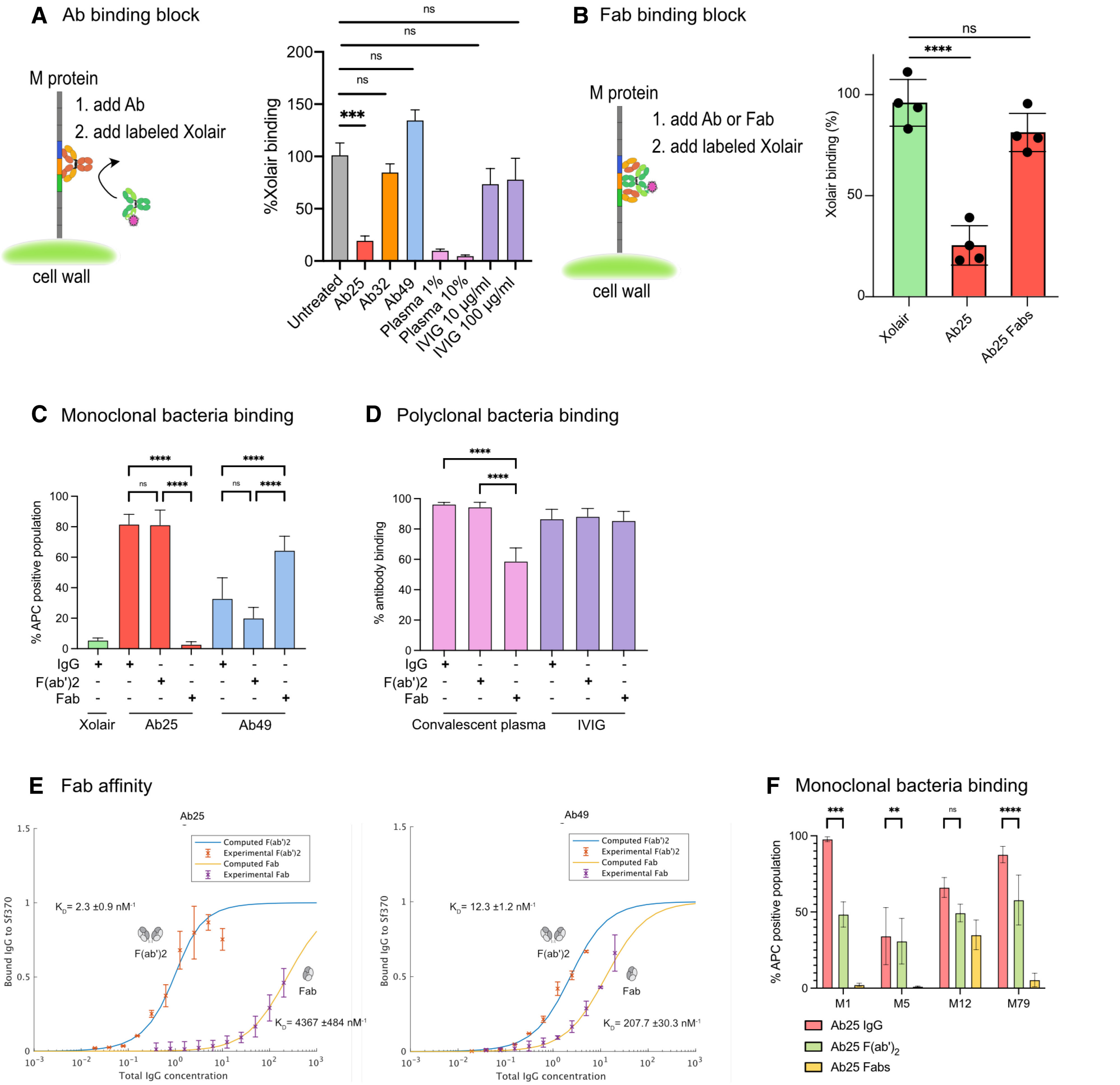

Figure 6. Dual-Fab mode of interaction is required for functional antibody binding.

A Binding of Alexa647-conjugated Xolair (100 μg/ml) to SF370 after pretreatment with indicated antibody samples (10 μg/ml). The data is from three independent experiments pooled together and error bars represent the SEM.

B Heat-killed bacteria previously stained with Oregon Green were incubated with 10 μg/ml of Ab25 or IgdE-digested (Spoerry *et al*, 2016) Ab25 (Fabs). The bacteria were then incubated with fluorescently labeled Xolair (AF647) before being analyzed with flow cytometry. Bacteria that bound Xolair via its Fc binding are shown as positive. The data shown here is compiled from quadruplicate experiments, with error bars representing the SD.

C Binding properties of Xolair, IdeS-cleaved, or IgdE-cleaved Ab25 or Ab49 were studied as previously by mixing the antibodies with heat-killed, Oregon Green bacteria. The bacteria were then washed and stained with AlexaFluor 647-conjugated Fab anti-Fabs and analyzed by flow cytometry. Data is from four independent experiments with mean and SD shown.

D Binding properties of IdeS-cleaved or IgdE-cleaved convalescent donor plasma or IVIG were analyzed as in (D). Data is from four independent experiments with mean and SD shown.

E Affinities for Fabs generated by IgdE treatment of Ab25 and Ab49 were performed as described previously in Fig 1. The new Fab data is overlaid on the data from Fig 1. The data represent pooled results from four independent experiments K_D values for the fitting are given in each plot, together with a confidence interval calculated using the Bootstrap method.

F Binding properties of IdeS-cleaved or IgdE-cleaved Ab25 were studied by mixing the antibodies (20 μg/ml) with indicated strains of live GFP-expressing bacteria. The bacteria were then washed and stained with AlexaFluor 647-conjugated Fab anti-Fabs and analyzed by flow cytometry. Data is from three independent experiments with mean and SD shown.

Data information: Statistical significance was evaluated using two-way ANOVA with Tukey multiple comparison corrections and * denotes $P \leq 0.05$, *** for $P \leq 0.001$, and **** for $P \leq 0.0001$.

© 2022 The Authors

protective function. We took multiple approaches to verify these findings and elucidated the precise nature of Ab25's dual-Fab-binding capacity. We investigated the ability of single Ab25 Fabs to obstruct Fc binding. We used IgdE protease (Spoerry *et al*, 2016) to prepare intact single Fabs of Ab25. If the binding of single Fabs to either Ab25-binding site on M protein could sustain a steric hindrance, we should see a reduction in Fc binding. However, Fc binding was not affected by single Ab25 Fabs (Fig 6B), suggesting that binding occurs via dual-Fab binding. Full cleavage of the antibodies into Fabs was verified by SDS-PAGE analysis (Appendix Fig S3). We wanted to see how well different forms of the antibodies could bind to bacteria. We compared the binding of whole IgG, F(ab')$_2$, and single Fabs to SF370. Strikingly, Ab25 single Fabs could not bind to the bacteria, whereas Ab49 Fabs increased their binding (Fig 6C). The latter is the expected result since single Fabs should have easier access, yet the results reveal that Ab25's predominant mode of interaction with the M1 protein is mainly via dual-Fab cis binding and not via single Fabs. We attempted to perform M-peptide ELISA experiments to further validate the dual-Fab cis binding but only full-length M protein worked, so no further details could be extracted through that approach (data not shown). This is not surprising as the complexity of the M protein folding has been well-established (Nilson *et al*, 1995; Morfeldt *et al*, 2001).

The results using IgG-cleaving enzymes provide a possible way to investigate the occurrence of dual-Fab cis-binding antibodies in polyclonal samples. By treating convalescent donor plasma and IVIG with IdeS and IgdE, we saw no effect on binding with F(ab')$_2$ compared to intact IgG (Fig 6D). However, we saw a significant reduction (~ 40%) in binding only with the convalescent plasma when comparing single Fabs to IgG (Fig 6D). It should be noted that the IgdE enzyme only cleaves IgG1. This indicates that there could be a significant portion of dual-Fab cis-binding antibodies in GAS-convalescent individuals. To assess how much weaker the binding via single Fabs is, we performed affinity measurements of single Fabs. These showed that Ab25 Fab binding has a 2000-fold lower affinity (4.4 μM^{-1}) compared to F(ab')$_2$ binding (2.3 nM^{-1}, Fig 6E). There was still a question of whether dual-Fab cis binding occurs across different M types. To answer this, we performed similar binding experiments to those in Fig 6C but used live bacteria expressing GFP. We found that single Fab fragments of Ab25 lost binding to both M5 and M79, as seen with M1, while they retained binding to the M12 strain. In contrast, binding with F(ab')$_2$ occurred for all the strains (Fig 6F). This means that for M12 we cannot determine the mode of interaction, only that both single and dual-Fab are possible, and thus draw no conclusion of whether it is dual-Fab that mediated phagocytosis in the M12 case. Importantly, our data supports dual-Fab-mediated function for the A–C *emm* pattern types (such as M1 and M5 strains) and for the E *emm* type strain M79, where Ab25 only binds via dual-Fab interaction.

Discussion

In this study, we describe a new broadly binding monoclonal antibody (Ab25) that binds to the M protein's central region and mediates bacterial clearance. Interestingly, this antibody has a peculiar mode of interaction with its target as it interacts with the M protein at two distinct epitopes simultaneously. As the M protein is anchored via an LPXTG motif found prior to the D domain, these epitopes located in the B/S/C domains would be displayed on the bacterial surface, as we have also previously seen with EM images of antibody Fc binding to the S domain (Nordenfelt *et al*, 2012). We now understand that in addition to the classical antibody–antigen interactions, which are extensively studied, dual-Fab binding of antibodies to antigens could potentially enhance antibody function. Mass spectrometric analysis of anti-M antibodies cross-linked to M protein, coupled with biochemical and immunological analysis, revealed that dual-Fab cis binding represents a bivalent interaction around the M protein S region. Importantly, our data show that dual-Fab cis biding can occur across different M types, even when their sequences are not highly conserved. We confirmed the dual-Fab cis-binding phenomenon using three independent assays (cross-linking TX-MS, inhibition of Fc-mediated binding, and loss of single-Fab binding). This is a testament to the complexity of antigen–epitope interactions where binding requires recognizing compatible 3D structures.

This antibody–M protein interaction can mediate phagocyte engagement and streptococcal phagocytosis. As seen with Ab25, dual-Fab cis binding seems to be a contributing factor in the triggering of an immune response. In contrast, as seen with the closely located Ab49, single-Fab binding has little or no immunological effect in the assays tested. It has been previously shown that antibodies against the hypervariable region or C-repeats (Jones & Fischetti, 1988; Carlsson *et al*, 2003; Lannergård *et al*, 2015; Happonen *et al*, 2019) of the M protein can also be opsonic and mediate phagocytosis. We employed two independent approaches to assay phagocytosis, using THP-1 cells, and human blood infections. THP-1 cells are incubated with heat-killed bacteria and are analyzed by flow cytometry. The blood-based experiment, however, utilizes live bacteria and whole donor blood. This creates a more complex system that can discern nuances that require multiple cell types or molecules, such as complement activation. Both experimental approaches suggest that Ab25 is a potentially opsonic antibody across multiple GAS *emm* types. Therefore, while in the context of the M protein central region, dual-Fab-binding antibodies are opsonic, other functional interactions between antibodies and antigens clearly exist. It is interesting to note that earlier studies on M6 protein revealed the central M region to not only be conserved among many M proteins but also non-opsonic (Jones & Fischetti, 1988), mirroring the results we observe with Ab32 and Ab49. The dual-Fab interaction of Ab25 is correlated with it being opsonic while still retaining the cross-reactivity with M proteins due to the conserved nature of the central M region.

The fact that Ab25 and Ab49 bind to such similar locations, yet their binding leads to such different outcomes, provides both an excellent internal control for dual-Fab cis binding and prompts questions as to why it is beneficial for immune stimulation. From a structural perspective, a two-point attachment is much more stable than a one-point attachment, probably stabilizing IgGFc–Fcγ receptor interactions. It would also provide a consistent angle of interaction, which might aid the clustering of receptors. The angle is relevant because the presentation of IgGFc's pointing in different directions could interfere with the zipper-like mechanism that Fcγ receptors typically use during phagocytosis (Swanson, 2008). The specific orientation that Ab25 adapts when binding M protein results in the IgGFc being oriented perpendicular relative to M protein. This

© 2022 The Authors

happens to be an optimal angle at which Fcγ receptors interact with IgGFc (Sondermann *et al*, 2000). Instead, interactions between IgA and Fcα receptors would have benefitted from an antibody being rotated 90° from that of Ab25 (Herr *et al*, 2003). Recent findings indicate that both antigen height, where below 10 nm is beneficial (Bakalar *et al*, 2018) as well as pericellular barriers where transmembrane pickets obstruct the interaction (Freeman *et al*, 2018) are important factors to consider for effective phagocytosis. Given that all three of our monoclonals bind at a similar height (40–50 nm), this in and of itself cannot be an explanation here. However, it is possible that the stable dual-Fab interaction shifts the perceived antigen height and thus results in a closer bacteria–phagocyte interaction. In this case, the pericellular barrier would be reduced by the same distance, allowing a more effective phagocytic interaction. Another obvious mechanism could be the reduced IgGFc binding that Ab25 achieves by spanning the IgGFc-binding site (obstructing the S region). However, this is most likely not the case for two reasons. First, the IgGFc binding has a low affinity in the μM range and only becomes relevant at high IgG concentrations, magnitudes larger than those used in the phagocytosis experiments. Second, if the Fc binding could block phagocytosis, then the phagocytosis experiments with mixed monoclonals would see an additive effect, as Ab25 would block Fc binding, allowing the other antibodies to function. As we saw no additive effect whatsoever, this cannot be the case. The reduced IgGFc binding that Ab25 confers might have additional protective effects on the host that remain to be investigated in future studies.

The requirement of dual-Fab cis binding to separate epitopes that Ab25 exhibits, as demonstrated by experiments with single Fabs, is an unexpected mode of functional antibody interaction. Dual-Fab cis binding is thus adding to the already astounding diversity found in antibody-binding mechanisms (Kanyavuz *et al*, 2019). A related phenomenon to the dual-Fab binding is the case of the anti-HIV 2G12 antibody (Trkola *et al*, 1996), which has a mutation in its hinge region leading to Fab dimerization (Gach *et al*, 2010). The two fabs of 2G12 bind to high-mannose sugars but due to the fact of their unorthodox dimerization, essentially behave as one large Fab (Calarese *et al*, 2005). In fact, normal single Fab-based interactions between multiple anti-HIV glycan antibodies gave similar biological outcomes as 2G12 (for a review see Kong *et al*, 2014). This indicates that while 2G12 has an unorthodox structure, its function is correlated to the specific epitope and not due to a distinct mode of interaction. In the context of unorthodox antibodies; bispecific antibodies (Kontermann & Brinkmann, 2015) or antigen clasping antibodies (Hattori *et al*, 2016) have been engineered for improved functionality. We show through dual-Fab cis binding that evolution has resulted in similar outcomes. The results presented here reveal an, up till now, unknown added value of using F(ab')2 fragments rather than single Fabs when screening for functional antibodies. Whether dual-Fab binding is an interesting natural quirk that occurs in just one patient or if it happens in large cohorts of people during GAS infection remains to be established. Either way, the correlation between dual-Fab and immune function sheds light on the importance of this unorthodox antibody-binding phenomenon.

The evolutionary mechanisms leading to dual-Fab cis-binding antibodies may partly explain why children and adolescents suffer the most from recurring GAS infections (Oliver *et al*, 2018). The time and exposures required to generate dual-Fab cis binding or other unorthodox antibodies could be related to the observation that frequent infections are needed to generate long-term immunity (Pandey *et al*, 2016). We speculate that through multiple rounds of GAS infection during childhood, antibody hypermutation could generate dual-Fab cis-binding antibodies, providing one potential mechanism to bypass the elaborate bacterial immune evasive mechanisms. It is difficult to predict how common this mode of binding is and how important it is in humans. Still, it is not unlikely that other bacterial proteins (such as adhesins or other molecules with long repetitive structures) might be targeted by dual-Fab-binding antibodies (Kline *et al*, 2009).

Dual-Fab cis-binding antibodies might have implications for antibody function and vaccine design. Our findings could explain why attempts at generating an effective vaccine against GAS have so far been unsuccessful (Dale & Walker, 2020). Even if a vaccine can induce high antibody titers, a large portion of these antibodies could be nonfunctional. If it turns out that dual-Fab cis-binding antibodies are critical for function, antibody-based vaccine development strategies could be modified to take this into account. If vaccine peptides do not maintain conformational integrity (Skwarczynski & Toth, 2016) or span dual-Fab possible epitopes (10–15 nm separation), they might not be able to exhibit effective antibody-based immunity. Cell-mediated immunity would not be affected since T cell–MHC interaction, by definition, needs to be based on short peptides. The discovery and characterization of a putatively protective antibody against experimental GAS infection opens the possibility for monoclonal immunotherapy, but further validation of *in vivo* functions is required before that could be explored.

Materials and Methods

Reagents and Tools table

Reagent or Resource	Source	Identifier
Antibodies		
CD19-PE	BD	555413
CD3-BV510	BD	564713
IgG-BV421	BD	562581
Xolair (Omalizumab)	Novartis	28268

 © 2022 The Authors

Reagents and Tools table (continued)

Reagent or Resource	Source	Identifier
Alexa Fluor® 647 AffiniPure F(ab')$_2$ Fragment Goat Anti-Human IgG, F(ab')$_2$ fragment specific	Jackson Immunoresearch	109-606-097
Human IgG (H&L) Secondary Antibody Peroxidase Conjugated Pre-Adsorbed	Rockland (BioNordika)	609-103-123
Privigen, 100 mg/ml	Behring CSL	126684
Alexa Fluor® 647 AffiniPure F(ab')$_2$ Fragment Goat Anti-Human IgG, Fcγ Fragment Specific	Jackson Immunoresearch	109-606-170
CD29	Pharmingen	30861A
CD18 BV421	BD	743370
Anti-human Troponin type 3, Cardiac	Novus Biologicals	NB110-2546
Bacterial and Virus Strains		
MC25	Collin & Olsén (2001)	
Mix'n'go DH5a E. coli	Nordic Biolabs	T3002
Group A Streptococcus SF370 M1	ATCC	700294
Group A Streptococcus AP1 M1	World Health Organization	40/58; covS truncated
Biological Samples		
Donor blood, plasma, serum		
Chemicals, Peptides, and Recombinant Proteins		
Phosphate Buffered Saline PBS	GIBCO	10010-015
Bovine Serum Albumin	VWR	422351S
Sytox-FITC live/dead stain	Thermofisher	S34860
RPMI-1640	Sigma Aldrich	R8758-6X500ML
DMEM	Fisher scientific	11584516
Chloroquine diphosphate	Fisher scientific	15368865
Polyethylenimine (PEI)	Fisher scientific	11460630
OptiMEM	Life technologies	31985070
Todd-Hewitt Yeast media	Thermo scientific	CM0189
Oregon Green 488-X succinimidyl ester	Thermo scientific	O6147
Cypher5E NHS ester	Fisher scientific	11505834
Fibrinogen	Sigma Aldrich	F4883-5G
Protein G-HRP	Biorad	1706425
B1B2B3	Proteogenix	
C1C2C3	Proteogenix	
Supersignal west femto chemiluminescent	Fisher scientific	10391544
Paraformaldehyde 16%	Fisher scientific	10751395
Wheat Germ Agglutinin, Alexa Fluor™ 488 Conjugate	ThermoFisher	W11261
X5 Prolong gold antifade reagent	Fisher scientific	11569306
Quanti blue solution	Invivogen	rep-qbs
SiR-Actin	Cytoskeleton	CY-SC001
Cytochalasin D	ThermoFisher	PHZ1063
Critical Commercial Assays		
Rosettesep B	Stem cell technologies	15064
Lymphoprep	Stem cell technologies	7811
Alexa Fluor 647 using the microscale labeling kit	Invitrogen	A30009
OneStep RT-PCR kit	Qiagen	210212
Gammabind Protein G beads	Cytiva	11564945
Cytometric bead assay	BD Biosciences	552364

© 2022 The Authors

Reagents and Tools table (continued)

Reagent or Resource	Source	Identifier
ZymoPURE II™ Plasmid Midiprep Kit	Nordic Biosite	D4200
Fabalactica	Genovis	A2-AFK-025
Human FDA Standard Tissue MicroArray (Normal)	Novus Biologicals	NBP2-78123
Experimental Models: Cell Lines		
THP-1 cells	Sigma Aldrich	88081201-1VL
THP-1-Xblue cells	Invivogen	thp-nfkb
HEK293	Sigma Aldrich	12022001-1VL
Experimental Models: Organisms/Strains		
C57BL/6J mice	Scanbur/ Charles River Laboratories	
Oligonucleotides		
All nucleotides published previously		Smith *et al* (2009)
Recombinant DNA		
pGFP1	lab of Dr. Anna Norrby-Teglund	Constitutively expressed GFP

Methods and Protocols

Single B cell purification, baiting, and isolation

B cell isolation was performed as described previously (Smith *et al*, 2009), with some modifications. Briefly, 35 ml of blood was drawn (into citrated collection tubes) from a young woman who had recently recovered from a group A streptococcal infection. The woman gave written consent, and the collection and analysis of human blood samples were approved by the regional ethics committee, permit number 2015/801, in agreement with the principles set out in the WMA Declaration of Helsinki and the Department of Health and Human Services Belmont Report. The blood was treated with 2.5 µl/ml Rosettesep B (Stemcell technologies) for 20 min at room temperature. The blood was then diluted 1:1 in phosphate-buffered saline (PBS) and layered onto Lymphoprep gradients. After centrifugation (30 min at 800 *g*), the plasma was collected and frozen while the B cell layer (around 7 ml) was removed, diluted with 43 ml of PBS, and centrifuged again. This washing step was repeated twice. The B cells were counted and kept at room temperature for staining (typical yields are 2–5 million cells per 30–40 ml of blood).

B cell staining, baiting, and sorting

The B cells were concentrated into a final volume of 500 µl in PBS. The cells were then blocked with 5% BSA for 20 min before being stained with antibodies against CD19-PE [(BD-555413) 20 µl per 10^6 cells] CD3-BV510 [(BD-564713) 5 µl per 10^6 cells], and IgG-BV421 [(BD-562581) 5 µl per 10^6 cells]. The B cells were also labeled with the Sytox-FITC live/dead stain [(Thermofisher-S34860) 1:1000 dilution]. Baiting of the B cells was done using soluble M1 protein isolated from an MC25 group A streptococcus M1 strain. The M1 protein isolation procedure was previously described elsewhere (Collin & Olsén, 2000). The M1 protein was directly conjugated to Alexa Fluor 647 using the microscale labeling kit (Invitrogen). In addition to the antibodies and live/dead stains, 0.1 µg/ml of AF694-M1 was added to the cells. The mixture was incubated at 32°C for 20 min (M1 undergoes a conformational change at 4°C, which could obscure important epitopes; Cedervall *et al*, 1995). After the incubation, the cells were washed with PBS twice and were kept on ice until further analysis. The gates for sorting were set on a FACSAria-Fusion sorter using unstained cells and FMO-1 samples. Hundred cells were sorted from 2.5 million B cells directly into 10 µl of water containing Rnase inhibitor in 96-well plates and were immediately transferred to a −80°C freezer. The cells at this point would have been lysed due to osmotic pressure and the RNA stabilized in solution.

Reverse transcription, family identification, and cloning

The previously frozen cells in plates were thawed on ice, and RT-PCR was performed using the OneStep RT-PCR kit (Qiagen) protocol without modification. The primer sequences used in the PCR steps were taken directly from table 1 in the Smith *et al* (2009) paper without any modifications. After the RT-PCR, the nested PCR was performed, and the bands corresponding to the variable regions of the heavy and light chains were sequenced to identify the antibody families. Family-specific cloning primers were used to clone the variable chains into the plasmids containing the constant regions of the heavy (Human IgG1 subclass) and light chains (all the antibodies were kappa light chains). The expression plasmids used are from the AbVec series and were generously donated by Dr. Patrick Wilson's group.

General cell culture and transfection

THP-1 cells (Leukemic monocytes) were kindly provided by Dr. Arianne Neuman at Lund University and were maintained in RPMI media supplemented with L-glutamine and 10% FBS. The cells were kept at a cell density between 5–10 × 10^5 cells per ml. THP-1-Xblue cells were maintained like regular THP-1 cells. HEK293 (sourced from Sigma-Aldrich cat. 12022001) cells were maintained in DMEM supplemented with L-glutamine and 10% FBS. The cells were never allowed to grow to 100% confluency. The day before transfection, 8 × 10^6 cells were plated in circular 150 mm dishes. This

 © 2022 The Authors

transfection format allowed for the most efficient antibody recovery. All cell lines were routinely tested for *Mycoplasma* contamination.

Transfection, expression, and purification

In total, 10 antibody construct pairs were successfully generated from 100 starting cells. The antibody pairs were transformed into Mix'n'go *E. coli*. Transformant colonies were verified by sequencing and the plasmids were further propagated and DNA was extracted using a Zymoresearch midiprep kit. Using PEI transfection, plasmid pairs encoding full antibodies were co-transfected into HEK293 cells. Cells were briefly treated with 25 μM Chloroquine for 5 h. After that, 20 μg of heavy and light chain expression plasmid DNA were diluted in OptiMEM (Life technologies) media containing polyethylenimine (PEI) at a 1:3 ratio (for 50 μg of DNA, 114 μl of a 1 mg/ml PEI stock was used). The cells were incubated at 37°C for 18 h before they were washed 2 times with PBS, and the DMEM media was exchanged with OptiMEM. The cells were incubated for 72 h before the supernatants were collected. The antibodies in the supernatants were purified using Protein G beads in a column setup. The antibodies were then titrated by comparing their concentrations on an SDS-PAGE to serial dilutions of a known concentration of Xolair (commercially bought Omalizumab, stored at 150 mg/ml).

Bacterial strains, growth, and transformation

Streptococcus pyogenes strain SF370 (*emm1* serotype), and AP1 (*emm1* serotype), was grown in Todd-Hewitt Yeast media (THY) at 37°C. The bacteria were maintained on agar plates for 3 weeks before being discarded. We chose to use SF370 in all of our experiments since it is an M1 serotype strain lacking protein H, which is a complicating factor (due to its strong Fc-binding capacity and extensive homology with M protein; Åkesson *et al*, 1990). The different M serotypes used in our cross-strain comparison were clinical isolates previously deposited into our in-house biobank. For experiments, overnight cultures were prepared in THY and were diluted at 1:20 on the day of the experiments. After dilution, 3 h of growth at 37°C ensured that the bacteria were in mid-log growth. For the generation of GFP-expressing strains, the SF370 and its ΔM isogenic counterpart, as well as M5 (Manfredo), M12, M79, and M89 strains, were grown to mid-log before being washed with ice-cold water. The electrocompetent bacteria were electroporated with 20 μg of the pGFP1 plasmid and plated on Erythromycin supplemented THY plates. The successful transformants were fluorescent when examined under ultraviolet light. All strains were successfully transformed with the pGFP1 plasmid and yielded fluorescent colonies except for the M89 strain. Heat killing the bacteria was done by growing the cultures to mid-log, washing them once in PBS, and incubating them on ice for 5 min. The bacteria were then heat shocked at 80°C for 5 min before being placed on ice for 15 min. For the phagocytosis assay, the heat-killed bacteria were centrifuged at 8,000 *g* for 3 min and resuspended in Na-medium (5.6 mM glucose, 127 mM NaCl, 10.8 mM KCl, 2.4 mM KH_2PO_4, 1.6 mM $MgSO_4$, 10 mM HEPES, 1.8 mM $CaCl_2$; pH adjusted to 7.3 with NaOH). Under gentle rotation, heat-killed bacteria were stained with 5 μM Oregon Green 488-X succinimidyl ester (Thermofisher) at 37°C for 30 min. The bacteria were then centrifuged and resuspended in Sodium carbonate buffer (0.1 M, pH 9.0) for an additional staining step with the pH-sensitive dye CypHer5E (Fisher scientific). This was used at a concentration of 7 μg/ml in a volume of 1.5 ml for 2 h at room temperature under gentle rotation, protected from light. The samples were washed once with Na-medium to remove excess dye and stored at 4°C for later use.

NGS of bacterial genomes

Whole-genome sequencing was done at the Center for Translational Genomics at Lund University. NextSeq 550 Illumina sequencing was used to sequence the bacterial genomes. The genome sequencing data were searched against the CDC database of M protein families to detect the target M protein sequence. Each M protein sequence was pairwise aligned with the target M1 protein using EMBOSS Needle web server, and the result is reported. The sequences are available at NCBI SRA database, accession number PRJNA895198 (https://www.ncbi.nlm.nih.gov/sra/PRJNA895198).

Antibody screening and flow cytometry

For ELISAs: ELISA plates were coated overnight with 10 μg/ml M1 at 4°C, purified from MC25 culture supernatants (Collin & Olsén, 2000). After a 1-h incubation at 37°C, the wells were washed 3 times with PBST and blocked with 2% BSA in 300 μl PBST for 30 min. After blocking, 300 μl of antibody-containing supernatants were added to the wells or diluted donor plasma as a control. The samples were incubated for 1 h at 37°C, washed, and a Protein G-HRP solution (diluted 1:3,000) was added to the wells and incubated at 37°C for 1 h. The samples were then washed and developed with 100 μl developing reagent (20 ml substrate buffer NaCitrate pH 4.5 + 1 ml ABTS peroxide substrate +0.4 ml H_2O_2). Absorbance was read at OD_{450} following 5–30 min of color development at room temperature.

For flow cytometric screening: Overnights of SF370-GFP bacteria or its ΔM counterpart were diluted 1:20 into THY and grown until mid-log. In total, 100 μl of the bacteria were distributed into wells of a 96-well plate. Antibodies purified from cell culture supernatants were diluted to 5 μg/ml and were digested with 1 μg/ml of IdeS for 3 h at 37°C. The digested antibodies were further diluted 1:10 into the bacterial suspension, reaching a final concentration of 0.5 μg/ml. The bacteria were incubated for 30 min at 37°C before being washed twice with PBS. AF647-conjugated Fab α-Fab antibody fragments were used as secondary antibodies to detect binding of the primary α-M antibodies. After a 30-min incubation with the Fab α-Fab fragments, the bacteria were washed and analyzed on a *Cytoflex* flow cytometer (Beckman Coulter). The gates for the GFP-expressing bacteria were set using the SF370 parent strain (not expressing the GFP plasmid). GFP-expressing bacteria within the GFP-expressing gate were assessed for antibody staining (APC channel). Antibody staining reflects the presence of surface-bound primary-secondary antibody complexes and is indicative of bound anti-M antibodies.

For western blotting: Antibody reactivity to linear epitopes was assessed by probing the lysates of SF370 and its ΔM mutant using western blotting. Briefly, pellets of logarithmically grown bacteria were incubated with phospholipase C for 30 min in PBS until the lysates became clear. The lysates were sonicated and cleared by centrifugation (15,000 *g* for 3 min). We loaded 40 μg of 5 replicate sets of SF370 vs. ΔM mutant protein on a gradient SDS-PAGE gel (4–20%). The gel electrophoresis was run for 60 min to achieve protein separation. The proteins were transferred from the gel to a PVDF membrane which was blocked for 45 min with 5% skimmed

© 2022 The Authors

milk in PBST. The replicate lanes of the membrane were then cut and probed with 2 or 10 µg/ml of Xolair, Ab25, 32, 49, or IVIG overnight at 4°C. The membranes were washed 3 times with PBST and probed with the secondary HRP-conjugated goat antihuman IgG secondary (Rockland) antibody for 1 h at room temperature. The secondary was washed, and the membrane developed using a chemiluminescence reagent (WestFemto substrate, Thermofisher).

Tissue microarray immunohistochemistry

Tissue microarrays (Novus biologicals, NBP2-78123) containing 90 tissue sections from human donors were deparaffinized and rehydrated before antigen retrieval (boiling in 10 mM Sodium Citrate buffer (pH 6.0)). The tissue sections were permeabilized and blocked before staining with 10 µg/ml of Xolair, Ab25, 32, 49, or 2.5% donor plasma. IVIG was used at 500 µg/ml, whereas anti-Troponin was used at 1:100 dilution to replicate a cardiac tissue-reactive antibody. The HRP-conjugated Rockland anti-human IgG antibody (609-103-123) was used as a secondary antibody at a 1:250 dilution.

Agglutination assays

For agglutination assays:

Overnight cultures of SF370 and its ΔM strain were diluted 1:5 in RPMI and were treated with 100 µg/ml of the anti-M antibodies or with 5% donor plasma. It is crucial for this series of experiments that the bacteria are incubated in a cuvette and are not shaken or vortexed during incubation. At indicated time points, the OD_{600} of the bacteria was measured, and at the 3.5-h mark, the cuvettes were photographed.

For aggregate dissolution experiments:

SF370 bacteria were grown overnight, diluted 1:20 in THY, and left to grow for 2 h. The bacteria were then supplemented with 100 µg/ml of the appropriate antibody. Two hours after inoculation, the bacteria were vortexed, imaged (randomly), and the aggregate areas were analyzed using Image J by segmenting the bacteria through automatic processing and then quantifying the pixel area of the identified bacterial regions.

SIM imaging

Logarithmic phase bacteria were sonicated (VialTweeter; Hielscher) for 0.5 min to separate any aggregates and fixed in 4% paraformaldehyde for 5 min on ice. After that, the bacteria were washed with PBS twice (10,000 *g* for 3 min). SF370 was stained with Alexa Fluor 647-conjugated wheat germ agglutinin (WGA). Bacteria were incubated with IdeS-cleaved (F(ab') fragments) Xolair, Ab25, Ab32, and Ab49 and stained with fluorescently labeled IgGFab or IgGFc specific F(ab')2 fragments (DyLight488-conjugated antihuman IgGFc or IgGFab; Jackson ImmunoResearch Laboratory). Samples were mounted on glass slides using Prolong Gold Antifade Mountant with No. 1.5 coverslips. Images of single bacteria were acquired using an N-SIM microscope with LU-NV laser unit, CFI SR HP Apochromat TIRF 100X Oil objective (N.A. 1.49), and an additional 1.5× magnification. The camera used was ORCA-Flash 4.0 sCMOS camera (Hamamatsu Photonics K.K.), and the images were reconstructed with Nikon's SIM software on NIS-Elements Ar (NIS-A 6D and N-SIM Analysis). Images of the bacteria were acquired with 488 and 640 nm lasers. For site localization, single bacteria were manually identified and imaged in time series with 50 frames. The analysis pipeline for site localization was implemented in Julia and is available on GitHub (Kumra Ahnlide *et al*, 2022). A cutoff of the initial signal-to-noise ratio (SNR) was set to 0.3, and timeframes included were the ones with at least 70% of the initial SNR.

Binding curves

SF370 bacteria were grown to mid-log, washed, and 10 ml of culture were concentrated into 1,000 µl of PBS. The bacteria were stained with halving serial dilutions of the anti-M antibodies. In total, 30 µl of bacteria were used per every 100 µl of IdeS treated antibody. The staining was performed at 4°C for 30 min (with shaking) before the bacteria were washed and stained with an excess of AF647-conjugated Fab anti-Fab fragments in a volume of 30 µl for 30 min at 4°C with shaking. The bacteria were then diluted to 250 µl in PBS and analyzed by flow cytometry. The theoretical fit was done in MATLAB using fminsearch for an ideal binding curve with the dissociation constant as an unknown variable.

Cross-linking of antibody F(ab')2 fragments to the M1 protein

For the cross-linking of Ab25, Ab32, and Ab49 F(ab')2 fragments to the M1 protein, we used two different preparations of the M1 protein; one expressed and purified as recombinant in *E. coli* as described for the B1B2B3 and C1C2C3 constructs above, and one purified from the culture supernatant of the *S. pyogenes* MC25 strain (Collin & Olsén, 2000). The antibody F(ab')2 fragments were cleaved and purified from the expressed intact antibodies using the FragIT-kit with Fc-capture columns (Genovis) according to the manufacturer's instructions. For cross-linking, 25 µg of the recombinant M1 protein or 8 µg of the MC25 M1 protein were incubated with 5 µg of the respective F(ab')2 fragments in 1× PBS pH 7.4 at 37°C, 800 rpm, 30 min. Heavy/light disuccinimidyl suberate (DSS; DSS-H12/D12, Creative Molecules Inc.) resuspended in dimethylformamide (DMF) was added to final concentrations of 250 and 500 µM and incubated for a further of 60 min at 37°C, 800 rpm. The cross-linking reaction was quenched with a final concentration of 50 mM ammonium bicarbonate at 37°C, 800 rpm, 15 min.

Sample preparation for MS

The cross-linked samples were mixed with 8 M urea and 100 mM ammonium bicarbonate, and the cysteine bonds were reduced with 5 mM TCEP (37°C for 2 h, 800 rpm) and alkylated with 10 mM iodoacetamide (22°C for 30 min, in the dark). The proteins were first digested with 1 µg of sequencing grade lysyl endopeptidase (Wako Chemicals; 37°C, 800 rpm, 2 h). The samples were diluted with 100 mM ammonium bicarbonate to a final urea concentration of 1.5 M, and 1 µg sequencing grade trypsin (Promega) was added for further protein digestion (37°C, 800 rpm, 18 h). Samples were acidified (to a final pH 3.0) with 10% formic acid, and the peptides purified with C18 reverse phase spin columns according to the manufacturer's instructions (Macrospin columns, Harvard Apparatus). Peptides were dried in a speedvac and reconstituted in 2% acetonitrile and 0.2% formic acid before mass spectrometric analyses.

Liquid chromatography tandem mass spectrometry (LC–MS/MS)

All peptide analyses were performed on Q Exactive HF-X mass spectrometer (Thermo Scientific) connected to an EASY-nLC 1200 ultra-

© 2022 The Authors

high-performance liquid chromatography system (Thermo Scientific). Peptides were loaded onto an Acclaim PepMap 100 (75 µm × 2 cm) C18 (3 µm, 100 Å) pre-column and separated on an EASY-Spray column (Thermo Scientific; ID 75 µm × 50 cm, column temperature 45°C) operated at a constant pressure of 800 bar. A linear gradient from 4 to 45% of 80% acetonitrile in aqueous 0.1% formic acid was run for 65 min at a flow rate of 350 nl min-1. One full MS scan (resolution 60,000 @ 200 *m/z*; mass range 390–1,210 *m/z*) was followed by MS/MS scans (resolution 15,000 @ 200 *m/z*) of the 15 most abundant ion signals. The precursor ions were isolated with 2 *m/z* isolation width and fragmented using HCD at a normalized collision energy of 30. Charge state screening was enabled, and precursors with an unknown charge state and a charge state of 1 were rejected. The dynamic exclusion window was set to 10 s. The automatic gain control was set to 3e6 and 1e5 for MS and MS/MS with ion accumulation times of 110 and 60 ms, respectively. The intensity threshold for precursor ion selection was set to 1.7e4.

Computational modeling

Several protocols of Rosetta software suit (Koehler Leman *et al*, 2019) were employed for macromolecular modeling of this study. To model the full-length antibodies, first the antigen-binding domains were characterized using Rosetta antibody protocol (Weitzner *et al*, 2017). Then, comparative models have been generated for both heavy and light chains using RosettaCM protocol (Song *et al*, 2013) and aligned on the antigen-binding domains to represent the initial structure of the antibody. HSYMDOCK (Yan *et al*, 2018) and DaReUS_loop (Karami *et al*, 2019) web servers were used for symmetric docking of the Fc-domains and to model the hinge regions, respectively. Finally, 4 K models were produced for each antibody as the final refinement and the top-scored models were selected based on XLs derived from mass spectrometry combined with Rosetta energy scores. Moreover, to characterize the M1 antibody interactions, TX-MS protocol was used (Hauri *et al*, 2019), through which 2 K docking models were generated and filtered out using distance constraints from DDA data. A final round of high-resolution modeling was performed on top models to repack the sidechains using RosettaDock protocol (Gray, 2006).

Fluorescent Xolair competition experiments

Logarithmically grown SF370 bacteria were heat killed and labeled with Oregon Green (as described previously). The bacteria were mixed with antibodies or plasma/IVIG and incubated for 30 min at 37°C while shaking. Fluorescently conjugated Xolair (conjugated to Alexafluor 647 using the protein labeling kit (Invitrogen) according to the manufacturer's instructions) was then added to the bacteria at a concentration of 100 µg/ml for an additional 30 min before being directly analyzed by flow cytometry. For experiments in which Fabs were used, the Fabs were generated using the Fabalactica digestion kit (Genovis) according to the manufacturer's instructions.

Imaging-based binding assays

All images were acquired using an inverted Nikon Ti2-E widefield fluoresence microscope using a Plan Apo 20X objective (NA = 0.75). Fluorescence was excited with the SPECTRA X light engine® (Lumencore inc, Beaverton, OR, USA) and collected with a Nikon DS-Qi2 CMOS controlled with NIS-Elements AR (v5.21.02). Multiple stage coordinates were automatically generated and imaged using NIS-Elements JOBs and a Nikon motorized stage.

For image analysis, cells (BV421) and bacteria (Green, FITC) were segmented based on a background threshold algorithm for each channel separately. The cell masks were dilated to account for the whole cell area. Interaction between cells and bacteria was quantified by generating masks where cell and bacteria overlap, and the area and intensity profiles (mean, median, std) within this region were measured. Described image analysis was written in Julia (v1.6.0).

Phagocytosis assay

The phagocytosis experiments were performed using persistent association normalization (de Neergaard *et al*, 2021). Before opsonization, the CypHer5E- and Oregon Green-stained SF370 bacteria were sonicated for up to 5 min (VialTweeter; Hielscher) to disperse any large aggregates of bacteria. Sonication was deemed sufficient when clump dispersal was confirmed by microscopy. Staining and bacterial count (events/µl in the FITC +ve gate) was assessed by flow cytometry (CytoFLEX, Beckman-Coulter). The pH responsiveness of CypHer5E was tested by measuring the bacterial fluorescent staining in the APC channel before and after the addition of 1 µl of sodium acetate (3 M, pH 5.0) to 100 µl of the bacterial suspension. The presence of an acid-induced shift in fluorescence indicated successful staining. On the day of experiments, the appropriate number of bacteria were opsonized to suit each experiment. The opsonization with our M-specific antibodies, Xolair or IVIG, was performed at 37°C for 30 min. For experiments with a variable MOP, serial dilutions of the opsonized bacteria were made and used to incubate with the THP-1 cells. By gating on the leukocyte population (Fig EV4A), specifically on single cells, we were able to group the cells into those associated with bacteria (FITC positive) and with internalized bacteria (FITC and APC positive). The panels in Fig EV4A show noninteracting cells compared to interacting cells as a result of phagocytosis at 4°C (or Cytochalasin D in Fig EV4B) and 37°C, respectively. In experiments where antibody concentration was the variable, serial dilutions of the antibodies were made in Na-medium in 96-well plates, and the bacteria were directly added to the antibodies for opsonization. THP-1 cells were washed in PBS on the day of the experiment and resuspended in Na-medium. The concentration of THP-1 cells was measured prior to phagocytosis by flow cytometry and adjusted to 2000 cells/µl (100,000 cells per well). The cells were then added to the 96-well plates previously prepared with varying concentrations of previously opsonized bacteria (MOP) or with different antibody concentrations. Finally, 50 µl of THP-1 cells were added on ice resulting in a final phagocytic volume of 150 µl. After a 5-min incubation on ice, the plate was directly transferred to a shaking heating block set to 37°C while being protected from light or kept on ice as a control for internalization. Phagocytosis was stopped by putting the samples on ice for at least 15 min before data acquisition. Three experiments were performed to assess the association curves, and four experiments were performed at MOP 400 to compare different antibodies.

For blood phagocytosis experiments, blood was drawn into heparinized tubes and was used immediately. Bacterial overnight cultures were diluted and grown to mid-log, washed with PBS, and resuspended in Na-medium. The 4 strains used in these experiments (emm1, 79, 12, and 5) were the ones competent to be transformed

© 2022 The Authors

with the pGFP plasmid that also cover a range of different emm strains. The bacteria were opsonized with 10 μg/ml of Xolair or Ab25 for 30 min at 37°C. After opsonization, the bacteria were sonicated for 1 min and analyzed by flow cytometry for confirming live bacteria which were then used to inoculate the blood (which was diluted 10-fold in Na-medium). The infection was allowed to proceed for 30 min at 37°C in 96-well plates while shaking. The infection was then terminated by adding 1% paraformaldehyde for 30 min at room temperature. The cells were then washed with PBS and stained with SiR-Actin (emission at 674 nm) an anti-CD18 mAb (in BV421) which stains leukocytes. The cells were washed again and immobilized onto glass-bottom 96-well plates which were coated with an anti-CD29 antibody to capture cells. The wells were washed twice with PBS and imaged. More than 560 cells were imaged and their data quantified to generate the presented figures.

Flow cytometric acquisition was performed using a CytoFLEX (Beckman-Coulter) with 488 nm and 638 nm lasers and filters 525/40 FITC and 660/10 APC. Threshold was set at FSC-H 70000 for phagocytosis and for bacteria FSC-H 2000 and SSC-H 2000. Gain was set to 3 for FITC and 265 for APC. Acquisition was set to capture at least 5,000 events of the target population with a velocity of 30 μl/min taking approximately 30 min to assess all samples. The 96-well plate was kept on an ice-cold insert throughout the data acquisition to inhibit further phagocytosis.

NF-κB activity luciferase assay

THP-XBlue-CD14 (Invivogen) cells were seeded at a density of 200,000 cells per well in 96-well plates. The cells were treated with the appropriate antibodies (at 0.5 μg/ml) with or without M1 protein (2 μg/ml) for 18 h at 37°C. After the incubation, 20 μl of the cell supernatant were aspirated and mixed with the developing reagent, as described by the assay instructions (QuantiBlue solution, Invivogen). The samples were incubated at 37°C until development was appropriate and the OD650 measurement of the samples was done using a multiwell spectrophotometer.

Animal model

The local Malmö/Lund Institutional Animal Care Care and Use Committee approved all animal use and procedures, ethical permit number 03681-2019. Nine-week-old female C57BL/6J mice (Scanbur/ Charles River Laboratories) were used. Mice were housed in Innovive cages at 2–5 animals per cage. The mice had free access to food, water, and environmental enrichment and were checked at least once a day. Monoclonal antibody Ab25 (0.4 mg/mouse), or intravenous immunoglobulin (10 mg/mouse), was administered intraperitoneally 6 h preinfection. The pretreatment groups were coded and the experimenters blinded to which group had which intervention. *S. pyogenes* AP1 were grown to logarithmic phase in Todd–Hewitt broth (37°C, 5% CO_2). Bacteria were washed and resuspended in sterile PBS. In total, 10^6 CFU of bacteria were injected subcutaneously into the scruff leading to systemic infection within 24 h. Mice were sacrificed 24 h post-infection, and organs (blood, livers, spleens, and kidneys) were harvested to determine the degree of bacterial dissemination (reported as CFU/g of tissue). The blood cell counts were analyzed by flow cytometry. Cytokines were quantified using a cytometric bead assay (CBA mouse inflammation kit, BD) according to manufacturer's instructions.

The paper explained

Problem

The streptococcal M protein is a major virulence factor that mediates bacterial adhesion and immune evasion. No human antibodies have been generated against the M protein, partly due to its inherent antibody-binding ability.

Results

We have generated and characterized three human anti-M monoclonal antibodies. Among these, only one antibody (Ab25) mediates immune effector function. We discovered that Ab25 employs an unexpected mode of antigen–antibody interaction, which we termed dual-Fab cis binding. Dual-Fab cis-binding antibodies function by binding two nonidentical epitopes on its target, which in our case is the M protein. Strikingly, compared to a single-Fab-binding antibody (Ab49) which shares one of Ab25's two binding sites, only the dual-Fab antibody exhibits strong immune function.

Impact

The existence of dual-Fab binding as an antibody–antigen interaction modality and its influential role in mediating immune function was until this report unknown. This is particularly important since the antibody response against the M protein is not always opsonic and can lack immune function. The prevalence of this phenomenon and how it influences immune outcomes upon vaccination or infection is an important factor to consider in the future development of anti-streptococcal therapeutics.

Statistics

Statistical tests were performed as indicated in the figure legends, see Appendix Table S1.

Data availability

The bacterial sequencing data is available at https://www.ncbi.nlm.nih.gov/sra/PRJNA895198. Antibody amino acid sequences are protected under patent application (P023265EP1).

Expanded View for this article is available online.

Acknowledgements

WB, LH, OS, LB, LM, JM and PN were funded by the Knut and Alice Wallenberg Foundation. OS, JM and PN weer funded by Vetenskapsrådet (2018-05795). PN was funded by the Crafoord Foundation. TH and equipment were funded by IngaBritt och Arne Lundbergs Forskningsstiftelse. HK is funded by Swiss National Science Foundation (grant no. P2ZHP3_191289). The production of the recombinant M1 and the B1B2B3 and C1C2C3 constructs was performed at the Lund Protein Production Platform, Lund University, Sweden (http://www.lu.se/lp3). We thank Åsa Pettersson for help with flow sorting, Dr. Berit Olofsson for antibody production and Gisela Hovold for technical assistance. We thank Center for Translational Genomics, Lund University and Clinical Genomics Lund, SciLifeLab for providing sequencing service. We thank Lund University Bioimaging Center (LBIC) for providing microscope services.

Author contributions

Wael Bahnan: Conceptualization; resources; data curation; formal analysis; validation; investigation; visualization; methodology; writing – original draft;

© 2022 The Authors

writing – review and editing. **Lotta Happonen:** Data curation; formal analysis; validation; investigation; visualization; methodology; writing – review and editing. **Hamed Khakzad:** Data curation; formal analysis; validation; investigation; visualization; methodology; writing – review and editing. **Vibha Kumra Ahnlide:** Data curation; formal analysis; validation; investigation; visualization; methodology; writing – review and editing. **Therese de Neergaard:** Data curation; formal analysis; supervision; validation; investigation; visualization; methodology; writing – review and editing. **Sebastian Wrighton:** Formal analysis; validation; investigation; visualization; methodology; writing – review and editing. **Oscar André:** Formal analysis; validation; investigation; visualization; methodology; writing – review and editing. **Eleni Bratanis:** Data curation; formal analysis; validation; investigation; visualization; methodology; writing – review and editing. **Di Tang:** Data curation; formal analysis; validation; investigation; methodology; writing – review and editing. **Thomas Hellmark:** Resources; data curation; funding acquisition; methodology; writing – review and editing. **Lars Björck:** Conceptualization; resources; data curation; funding acquisition; writing – review and editing. **Oonagh Shannon:** Conceptualization; data curation; formal analysis; supervision; funding acquisition; validation; investigation; visualization; methodology; writing – review and editing. **Lars Malmström:** Conceptualization; resources; data curation; software; formal analysis; supervision; funding acquisition; validation; investigation; visualization; methodology; writing – review and editing. **Johan Malmström:** Conceptualization; resources; data curation; software; supervision; funding acquisition; validation; visualization; project administration; writing – review and editing. **Pontus Nordenfelt:** Conceptualization; resources; data curation; formal analysis; supervision; funding acquisition; visualization; methodology; writing – original draft; project administration; writing – review and editing.

Disclosure and competing interests statement

WB, LH, HK, OS, LB, LM, JM, and PN have a patent application pending (P023265EP1) based on the findings in this manuscript.

References

Åkesson P, Cooney J, Kishimoto F, Björck L (1990) Protein H—A novel igg binding bacterial protein. *Mol Immunol* 27: 523 – 531

Åkesson P, Schmidt KH, Cooney J, Björck L (1994) M1 protein and protein H: IgGFc- and albumin-binding streptococcal surface proteins encoded by adjacent genes. *Biochem J* 300: 877 – 886

Azuar A, Jin W, Mukaida S, Hussein WM, Toth I, Skwarczynski M (2019) Recent advances in the development of peptide vaccines and their delivery systems against group a *Streptococcus*. *Vaccines (Basel)* 7: 58

Bakalar MH, Joffe AM, Schmid EM, Son S, Podolski M, Fletcher DA (2018) Size-dependent segregation controls macrophage phagocytosis of antibody-opsonized targets. *Cell* 174: 131 – 142

Bournazos S, Wang TT, Dahan R, Maamary J, Ravetch JV (2017) Signaling by antibodies: recent progress. *Annu Rev Immunol* 35: 285 – 311

Burton DR, Hangartner L (2016) Broadly neutralizing antibodies to HIV and their role in vaccine design. *Annu Rev Immunol* 34: 635 – 659

Calarese DA, Lee H-K, Huang C-Y, Best MD, Astronomo RD, Stanfield RL, Katinger H, Burton DR, Wong C-H, Wilson IA (2005) Dissection of the carbohydrate specificity of the broadly neutralizing anti-HIV-1 antibody 2G12. *Proc Natl Acad Sci U S A* 102: 13372 – 13377

Carapetis JR, Steer AC, Mulholland EK, Weber M (2005) The global burden of group a streptococcal diseases. *Lancet Infect Dis* 5: 685 – 694

Carlsson F, Berggård K, Stålhammar-Carlemalm M, Lindahl G (2003) Evasion of phagocytosis through cooperation between two ligand-binding regions in streptococcus pyogenes M protein. *J Exp Med* 198: 1057 – 1068

Carlsson F, Sandin C, Lindahl G (2005) Human fibrinogen bound to streptococcus pyogenes M protein inhibits complement deposition via the classical pathway. *Mol Microbiol* 56: 28 – 39

Cedervall T, Åkesson P, Stenberg L, Herrmann A, Åkerström B (1995) Allosteric and temperature effects on the plasma protein binding by streptococcal M protein family members. *Scand J Immunol* 42: 433 – 441

Collin M, Olsén A (2000) Generation of a mature streptococcal cysteine proteinase is dependent on cell wall-anchored M1 protein. *Mol Microbiol* 36: 1306 – 1318

Collin M, Olsén A (2001) EndoS, a novel secreted protein from *Streptococcus pyogenes* with endoglycosidase activity on human IgG. *EMBO J* 20: 3046 – 3055

Cunningham MW, McCormack JM, Fenderson PG, Ho MK, Beachey EH, Dale JB (1989) Human and murine antibodies cross-reactive with streptococcal M protein and myosin recognize the sequence GLN-LYS-SER-LYS-GLN in M protein. *J Immunol* 143: 2677 – 2683

Dale JB, Walker MJ (2020) Update on group a streptococcal vaccine development. *Curr Opin Infect Dis* 33: 244 – 250

Freeman SA, Vega A, Riedl M, Collins RF, Ostrowski PP, Woods EC, Bertozzi CR, Tammi MI, Lidke DS, Johnson P *et al* (2018) Transmembrane pickets connect Cyto- and Pericellular skeletons forming barriers to receptor engagement. *Cell* 172: 305 – 317

Frick IM, Mörgelin M, Björck L (2000) Virulent aggregates of streptococcus pyogenes are generated by homophilic protein-protein interactions. *Mol Microbiol* 37: 1232 – 1247

Gach JS, Furtmüller PG, Quendler H, Messner P, Wagner R, Katinger H, Kunert R (2010) Proline is not uniquely capable of providing the pivot point for domain swapping in 2G12, a broadly neutralizing antibody against HIV-1. *J Biol Chem* 285: 1122 – 1127

Ghosh P (2018) Variation, indispensability, and masking in the M protein. *Trends Microbiol* 26: 132 – 144

Gray JJ (2006) High-resolution protein-protein docking. *Curr Opin Struct Biol* 16: 183 – 193

Happonen L, Hauri S, Svensson Birkedal G, Karlsson C, de Neergaard T, Khakzad H, Nordenfelt P, Wikström M, Wisniewska M, Björck L *et al* (2019) A quantitative streptococcus pyogenes-human protein-protein interaction map reveals localization of opsonizing antibodies. *Nat Commun* 10: 2727

Hattori T, Lai D, Dementieva IS, Montaño SP, Kurosawa K, Zheng Y, Akin LR, Świst-Rosowska KM, Grzybowski AT, Koide A *et al* (2016) Antigen clasping by two antigen-binding sites of an exceptionally specific antibody for histone methylation. *Proc Natl Acad Sci U S A* 113: 2092 – 2097

Hauri S, Khakzad H, Happonen L, Teleman J, Malmström J, Malmström L (2019) Rapid determination of quaternary protein structures in complex biological samples. *Nat Commun* 10: 192

Herr AB, Ballister ER, Bjorkman PJ (2003) Insights into IgA-mediated immune responses from the crystal structures of human FcalphaRI and its complex with IgA1-fc. *Nature* 423: 614 – 620

Herwald H, Cramer H, Mörgelin M, Russell W, Sollenberg U, Norrby-Teglund A, Flodgaard H, Lindbom L, Björck L (2004) M protein, a classical bacterial virulence determinant, forms complexes with fibrinogen that induce vascular leakage. *Cell* 116: 367 – 379

© 2022 The Authors

Jones KF, Fischetti VA (1988) The importance of the location of antibody binding on the M6 protein for opsonization and phagocytosis of group a M6 streptococci. *J Exp Med* 167: 1114 – 1123

Kadri SS, Swihart BJ, Bonne SL, Hohmann SF, Hennessy LV, Louras P, Evans HL, Rhee C, Suffredini AF, Hooper DC *et al* (2017) Impact of intravenous immunoglobulin on survival in necrotizing fasciitis with vasopressor-dependent shock: a propensity score-matched analysis from 130 US hospitals. *Clin Infect Dis* 64: 877 – 885

Kanyavuz A, Marey-Jarossay A, Lacroix-Desmazes S, Dimitrov JD (2019) Breaking the law: unconventional strategies for antibody diversification. *Nat Rev Immunol* 19: 355 – 368

Karami Y, Rey J, Postic G, Murail S, Tufféry P, de Vries SJ (2019) DaReUS-loop: a web server to model multiple loops in homology models. *Nucleic Acids Res* 47: W423 – W428

Klein JS, Bjorkman PJ (2010) Few and far between: how HIV may be evading antibody avidity. *PLoS Pathog* 6: e1000908

Kline KA, Fälker S, Dahlberg S, Normark S, Henriques-Normark B (2009) Bacterial adhesins in host-microbe interactions. *Cell Host Microbe* 5: 580 – 592

Koehler Leman J, Weitzner BD, Lewis SM, Consortium R, Bonneau R (2019) Macromolecular modeling and design in Rosetta: new methods and frameworks. *Nat Methods* 17: 665 – 680

Kong L, Stanfield RL, Wilson IA (2014) Molecular recognition of HIV glycans by antibodies. In *HIV Glycans in infection and immunity*, Pantophlet R (ed), pp 117 – 141. Springer New York: New York, NY

Kontermann RE, Brinkmann U (2015) Bispecific antibodies. *Drug Discov Today* 20: 838 – 847

Kumra Ahnlide V, Kumra Ahnlide J, Wrighton S, Beech JP, Nordenfelt P (2022) Nanoscale binding site localization by molecular distance estimation on native cell surfaces using topological image averaging. *Elife* 11: e64709

Kurosaki T, Kometani K, Ise W (2015) Memory B cells. *Nat Rev Immunol* 15: 149 – 159

Lannergård J, Kristensen BM, Gustafsson MCU, Persson JJ, Norrby-Teglund A, Stålhammar-Carlemalm M, Lindahl G (2015) Sequence variability is correlated with weak immunogenicity in streptococcus pyogenes M protein. *Microbiology* 4: 774 – 789

Lindahl G (2020) Subdominance in antibody responses: implications for vaccine development. *Microbiol Mol Biol Rev* 85: e00078-20

Linnér A, Darenberg J, Sjölin J, Henriques-Normark B, Norrby-Teglund A (2014) Clinical efficacy of polyspecific intravenous immunoglobulin therapy in patients with streptococcal toxic shock syndrome: a comparative observational study. *Clin Infect Dis* 59: 851 – 857

Lu LL, Suscovich TJ, Fortune SM, Alter G (2018) Beyond binding: antibody effector functions in infectious diseases. *Nat Rev Immunol* 18: 46 – 61

Macheboeuf P, Buffalo C, Fu C, Zinkernagel AS, Cole JN, Johnson JE, Nizet V, Ghosh P (2011) Streptococcal M1 protein constructs a pathological host fibrinogen network. *Nature* 472: 64 – 68

Madsen MB, Hjortrup PB, Hansen MB, Lange T, Norrby-Teglund A, Hyldegaard O, Perner A (2017) Immunoglobulin G for patients with necrotising soft tissue infection (INSTINCT): a randomised, blinded, placebo-controlled trial. *Intensive Care Med* 43: 1585 – 1593

McMillan DJ, Drèze PA, Vu T, Bessen DE, Guglielmini J, Steer AC, Carapetis JR, Van Melderen L, Sriprakash KS, Smeesters PR (2013) Updated model of group A *Streptococcus* M proteins based on a comprehensive worldwide study. *Clin Microbiol Infect* 19: E222 – E229

Mitchell TJ (2003) The pathogenesis of streptococcal infections: from tooth decay to meningitis. *Nat Rev Microbiol* 1: 219 – 230

Mitsi E, Roche AM, Reiné J, Zangari T, Owugha JT, Pennington SH, Gritzfeld JF, Wright AD, Collins AM, van Selm S *et al* (2017) Agglutination by anti-capsular polysaccharide antibody is associated with protection against experimental human pneumococcal carriage. *Mucosal Immunol* 10: 385 – 394

Moor K, Diard M, Sellin ME, Felmy B, Wotzka SY, Toska A, Bakkeren E, Arnoldini M, Bansept F, Co AD *et al* (2017) High-avidity IgA protects the intestine by enchaining growing bacteria. *Nature* 544: 498 – 502

Morfeldt E, Berggård K, Persson J, Drakenberg T, Johnsson E, Lindahl E, Linse S, Lindahl G (2001) Isolated hypervariable regions derived from streptococcal M proteins specifically bind human C4b-binding protein: implications for antigenic variation. *J Immunol* 167: 3870 – 3877

Motley MP, Banerjee K, Fries BC (2019) Monoclonal antibody-based therapies for bacterial infections. *Curr Opin Infect Dis* 32: 210 – 216

de Neergaard T, Sundwall M, Wrighton S, Nordenfelt P (2021) High-sensitivity assessment of phagocytosis by persistent association-based normalization. *J Immunol* 206: 214 – 224

Nilson BH, Frick IM, Åkesson P, Forsén S, Björck L, Akerström B, Wikström M (1995) Structure and stability of protein H and the M1 protein from streptococcus pyogenes. implications for other surface proteins of gram-positive bacteria. *Biochemistry* 34: 13688 – 13698

Nordenfelt P, Tapper H (2011) Phagosome dynamics during phagocytosis by neutrophils. *J Leukoc Biol* 90: 271 – 284

Nordenfelt P, Waldemarson S, Linder A, Mörgelin M, Karlsson C, Malmström J, Björck L (2012) Antibody orientation at bacterial surfaces is related to invasive infection. *J Exp Med* 209: 2367 – 2381

Okumura CYM, Nizet V (2014) Subterfuge and sabotage: evasion of host innate defenses by invasive gram-positive bacterial pathogens. *Annu Rev Microbiol* 68: 439 – 458

Oliver J, Malliya Wadu E, Pierse N, Moreland NJ, Williamson DA, Baker MG (2018) Group a streptococcus pharyngitis and pharyngeal carriage: a meta-analysis. *PLoS Negl Trop Dis* 12: e0006335

Ozberk V, Pandey M, Good MF (2018) Contribution of cryptic epitopes in designing a group a streptococcal vaccine. *Hum Vaccin Immunother* 14: 2034 – 2052

Pandey M, Ozberk V, Calcutt A, Langshaw E, Powell J, Rivera-Hernandez T, Ho M-F, Philips Z, Batzloff MR, Good MF (2016) Streptococcal immunity is constrained by lack of immunological memory following a single episode of pyoderma. *PLoS Pathog* 12: e1006122

Parks T, Wilson C, Curtis N, Norrby-Teglund A, Sriskandan S (2018) Polyspecific intravenous immunoglobulin in clindamycin-treated patients with streptococcal toxic shock syndrome: a systematic review and meta-analysis. *Clin Infect Dis* 67: 1434 – 1436

Pascual V, Liu YJ, Magalski A, de Bouteiller O, Banchereau J, Capra JD (1994) Analysis of somatic mutation in five B cell subsets of human tonsil. *J Exp Med* 180: 329 – 339

von Pawel-Rammingen U, Johansson BP, Björck L (2002) IdeS, a novel streptococcal cysteine proteinase with unique specificity for immunoglobulin G. *EMBO J* 21: 1607 – 1615

Sanderson-Smith M, De Oliveira DMP, Guglielmini J, McMillan DJ, Vu T, Holien JK, Henningham A, Steer AC, Bessen DE, Dale JB *et al* (2014) A systematic and functional classification of streptococcus pyogenes that serves as a new tool for molecular typing and vaccine development. *J Infect Dis* 210: 1325 – 1338

Skwarczynski M, Toth I (2016) Peptide-based synthetic vaccines. *Chem Sci* 7: 842 – 854

Smith K, Garman L, Wrammert J, Zheng N-Y, Capra JD, Ahmed R, Wilson PC (2009) Rapid generation of fully human monoclonal antibodies specific to a vaccinating antigen. *Nat Protoc* 4: 372 – 384

Sondermann P, Huber R, Oosthuizen V, Jacob U (2000) The 3.2-a crystal structure of the human IgG1 fc fragment-fc gammaRIII complex. *Nature* 406: 267 – 273

 © 2022 The Authors

Song Y, DiMaio F, Wang RY-R, Kim D, Miles C, Brunette T, Thompson J, Baker D (2013) High-resolution comparative modeling with RosettaCM. *Structure* 21: 1735 – 1742

Spoerry C, Hessle P, Lewis MJ, Paton L, Woof JM, von Pawel-Rammingen U (2016) Novel IgG-degrading enzymes of the IgdE protease family link substrate specificity to host tropism of streptococcus species. *PLoS One* 11: e0164809

Sriskandan S, Ferguson M, Elliot V, Faulkner L, Cohen J (2006) Human intravenous immunoglobulin for experimental streptococcal toxic shock: bacterial clearance and modulation of inflammation. *J Antimicrob Chemother* 58: 117 – 124

Staali L, Bauer S, Mörgelin M, Björck L, Tapper H (2006) *Streptococcus pyogenes* bacteria modulate membrane traffic in human neutrophils and selectively inhibit azurophilic granule fusion with phagosomes. *Cell Microbiol* 8: 690 – 703

Swanson JA (2008) Shaping cups into phagosomes and macropinosomes. *Nat Rev Mol Cell Biol* 9: 639 – 649

Trkola A, Purtscher M, Muster T, Ballaun C, Buchacher A, Sullivan N, Srinivasan K, Sodroski J, Moore JP, Katinger H (1996) Human monoclonal antibody 2G12 defines a distinctive neutralization epitope on the gp120 glycoprotein of human immunodeficiency virus type 1. *J Virol* 70: 1100 – 1108

Weineisen M, Sjöbring U, Fällman M, Andersson T (2004) Streptococcal M5 protein prevents neutrophil phagocytosis by interfering with CD11b/CD18 receptor-mediated association and signaling. *J Immunol* 172: 3798 – 3807

Weitzner BD, Jeliazkov JR, Lyskov S, Marze N, Kuroda D, Frick R, Adolf-Bryfogle J, Biswas N, Dunbrack RL, Gray JJ (2017) Modeling and docking of antibody structures with Rosetta. *Nat Protoc* 12: 401 – 416

Wilson PC, de Bouteiller O, Liu YJ, Potter K, Banchereau J, Capra JD, Pascual V (1998) Somatic hypermutation introduces insertions and deletions into immunoglobulin V genes. *J Exp Med* 187: 59 – 70

Woof JM, Burton DR (2004) Human antibody-Fc receptor interactions illuminated by crystal structures. *Nat Rev Immunol* 4: 89 – 99

Yan Y, Tao H, Huang S-Y (2018) HSYMDOCK: a docking web server for predicting the structure of protein homo-oligomers with Cn or Dn symmetry. *Nucleic Acids Res* 46: W423 – W431

License: This is an open access article under the terms of the Creative Commons Attribution License, which permits use, distribution and reproduction in any medium, provided the original work is properly cited.

Expanded View Figures

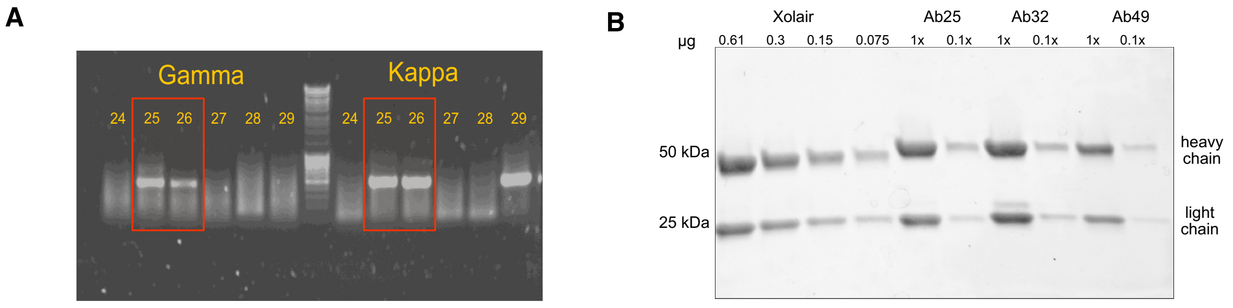

Figure EV1. Single cell RT-PCR and antibody quality control.

A RT-PCR was performed to amplify the VDJ and VJ regions coding for heavy and light variable chains, respectively. Two successful PCR examples are Ab25 and 26, shown in the red boxes with both heavy and light chains yielding amplicons.

B SDS-PAGE (4–20% gradient gel) analysis of Ab25, 32, and 49 compared to a serial dilution of Xolair. The gel was stained with Coomassie blue, destained, and imaged.

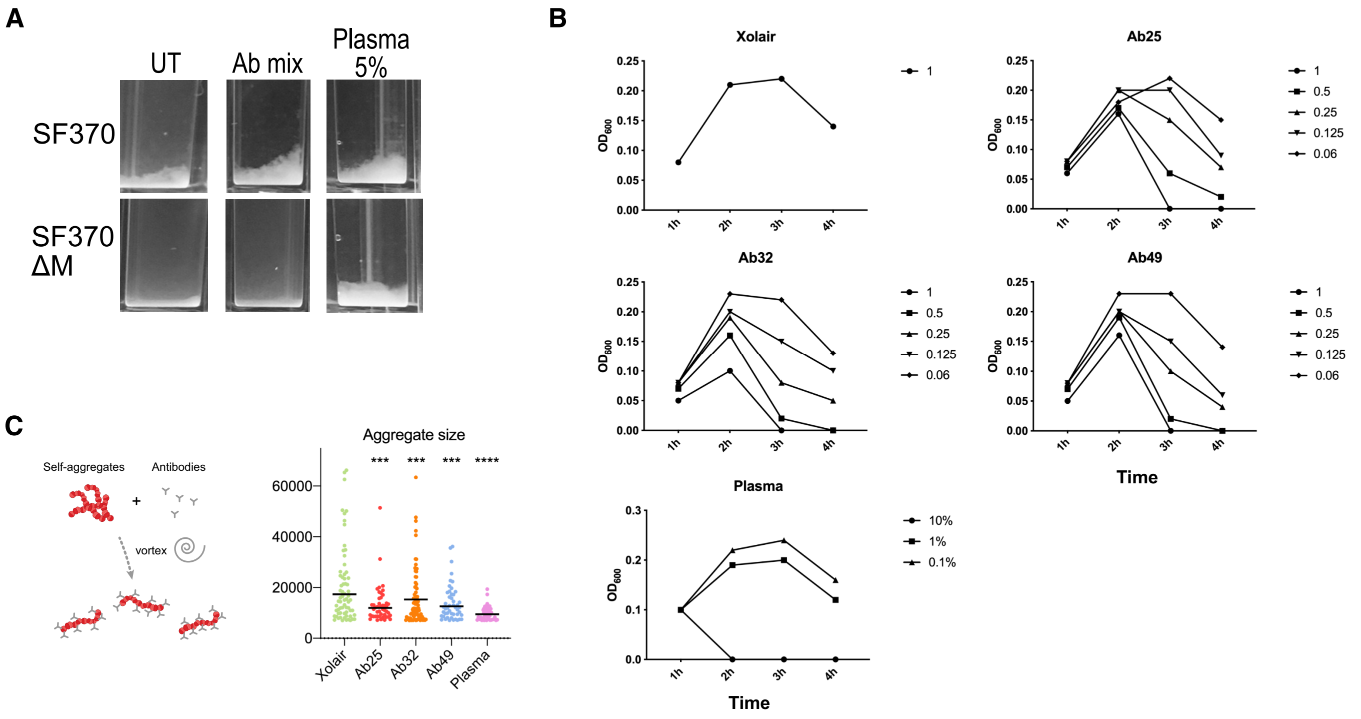

Figure EV2. Agglutination and aggregate dissolution data.

A SF370 overnight cultures were diluted 1:5 into THY and incubated with our antibodies (100 µg/ml) for 3 h in plastic cuvettes at 37°C before measuring their OD600. The agglutination effects of the antibody mix (Ab25, 32 and 49 at 100 µg/ml each) were compared on WT and ΔM bacteria. Donor plasma was used as a positive control.

B Dose–response experiment of antibody-mediated agglutination of bacteria, performed as in a with indicated antibody concentrations (µg/ml).

C Bacteria treated with individual antibodies were left standing for 3 h at 37°C before being vortexed. Aggregate size was quantified and tested using one-way ANOVA with multiple comparisons. The data in all the panels represents the results seen in at least three independent experiments. * denotes $P \leq 0.05$, ** for $P \leq 0.01$, *** for $P \leq 0.001$ and **** for $P \leq 0.0001$.

© 2022 The Authors

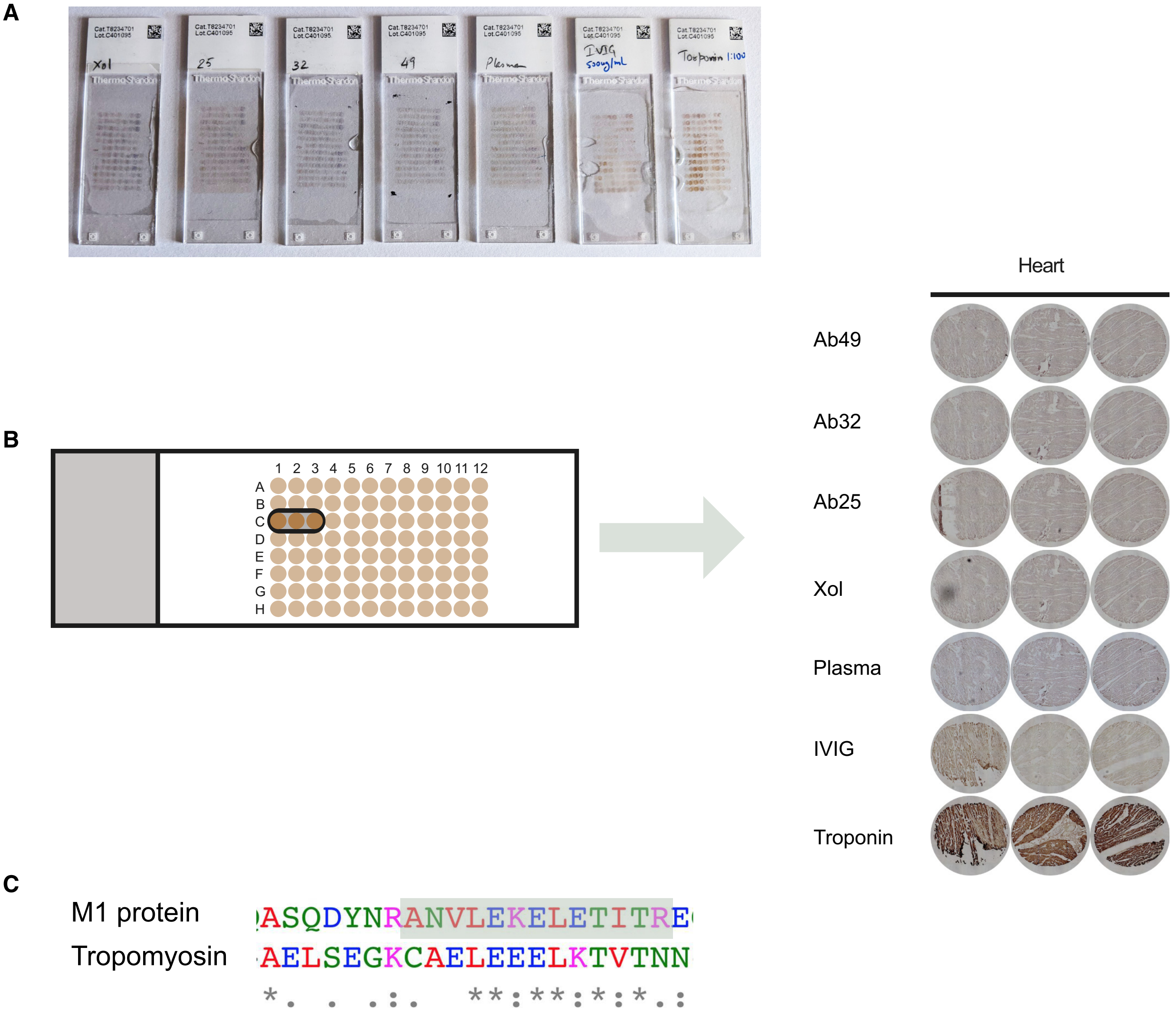

Figure EV3. Tissue microarray analysis for anti-M antibodies.

A Tissue microarray stained with 10 µg/ml of Xolair, Ab25, 32, and 49, or 2.5% plasma, 500 µg/ml IVIG or 1:100 anti-Troponin antibody. Each spot is a sampling from different tissue. Tissue sections are 6 µm thick, 1.5 mm wide, and were mounted on positively charged glass slides. None of the tissues showed reactivity with the monoclonal samples, except the positive control anti-Troponin, which was reactive with cardiac tissue, as shown. Out of the 30 tissue types present on the slides, the cardiac samples would otherwise be the tissue type that had the largest risk of reactivity due to the M-protein mimicry with heart tissue.

B Zoomed in images of tissue spots representing cardiac tissue.

C Sequence alignment between M1 protein and tropomyosin. The highlighted area indicates identified the cross-linked peptide (closest to the interaction site) between Ab25 and Ab49.

© 2022 The Authors

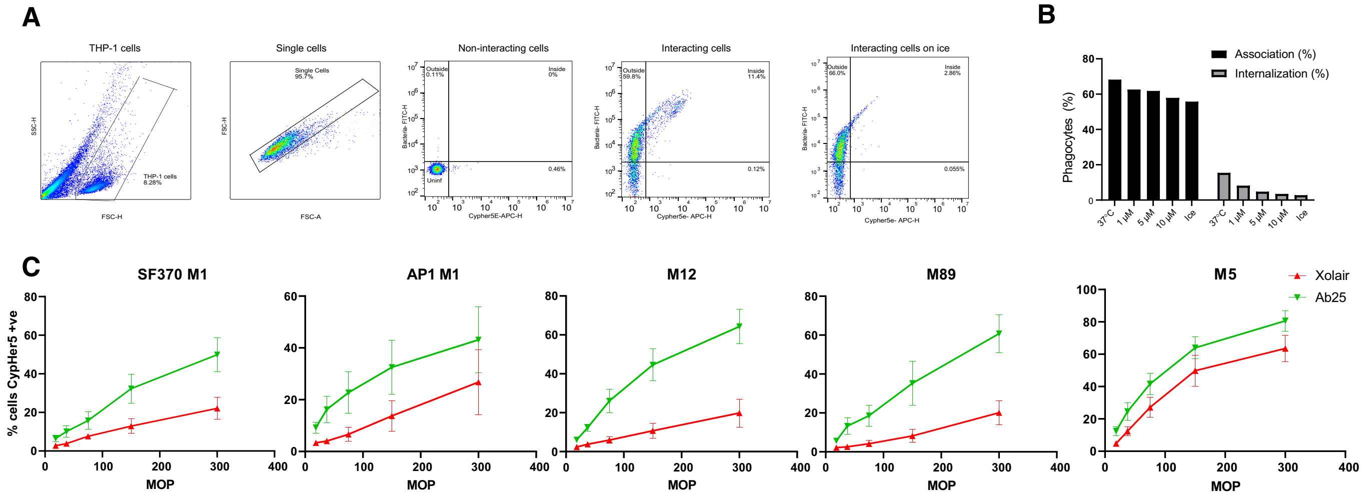

Figure EV4. Cross-strain phagocytosis of Ab25, controls for phagocytosis, and gating strategy for phagocytosis experiments.

A From left to right: THP-1 cells are gated based on FSC and SSC. Single cells were selected based on FSC—area and height parameters. Noninteracting cells that have no FITC (bacteria) or CypHer5E (internalized bacteria) signal. Interacting cells associated with bacteria show a shift to the upper left quadrant (outside), whereas THP-1 cells with internalized bacteria shift to the upper right quadrant due to acquisition of the APC signal. THP-1 cells inoculated with bacteria were kept on ice to reduce the phagocytosis. This is visible as a reduction in the number of events in the upper right quadrant.

B Fluorescent *E. coli* bacteria previously opsonized with 1 mg/ml of IVIG beads were incubated with THP-1 cells as in (A) at an MOP 100. In addition to the experiment at 37°C, interactions were allowed to occur on ice or with 1,5, or 10 μM Cytochalasin D. The cells were then analyzed by flow cytometry and the % of cells which had associated with bacteria (internalized or attached) or internalized bacteria were quantified.

C 10 μg/ml of Xolair or Ab25 were used to study phagocytic enhancement across different M serotypes. GAS with serotypes M1 (AP1), M12, M89, and M5 were compared in addition to the M1 SF370 strain. The effect of the antibody treatment on internalization rate was measured using flow cytometry. The error bars represent the standard error of the mean (SEM). The data is from three independent experiments.

© 2022 The Authors

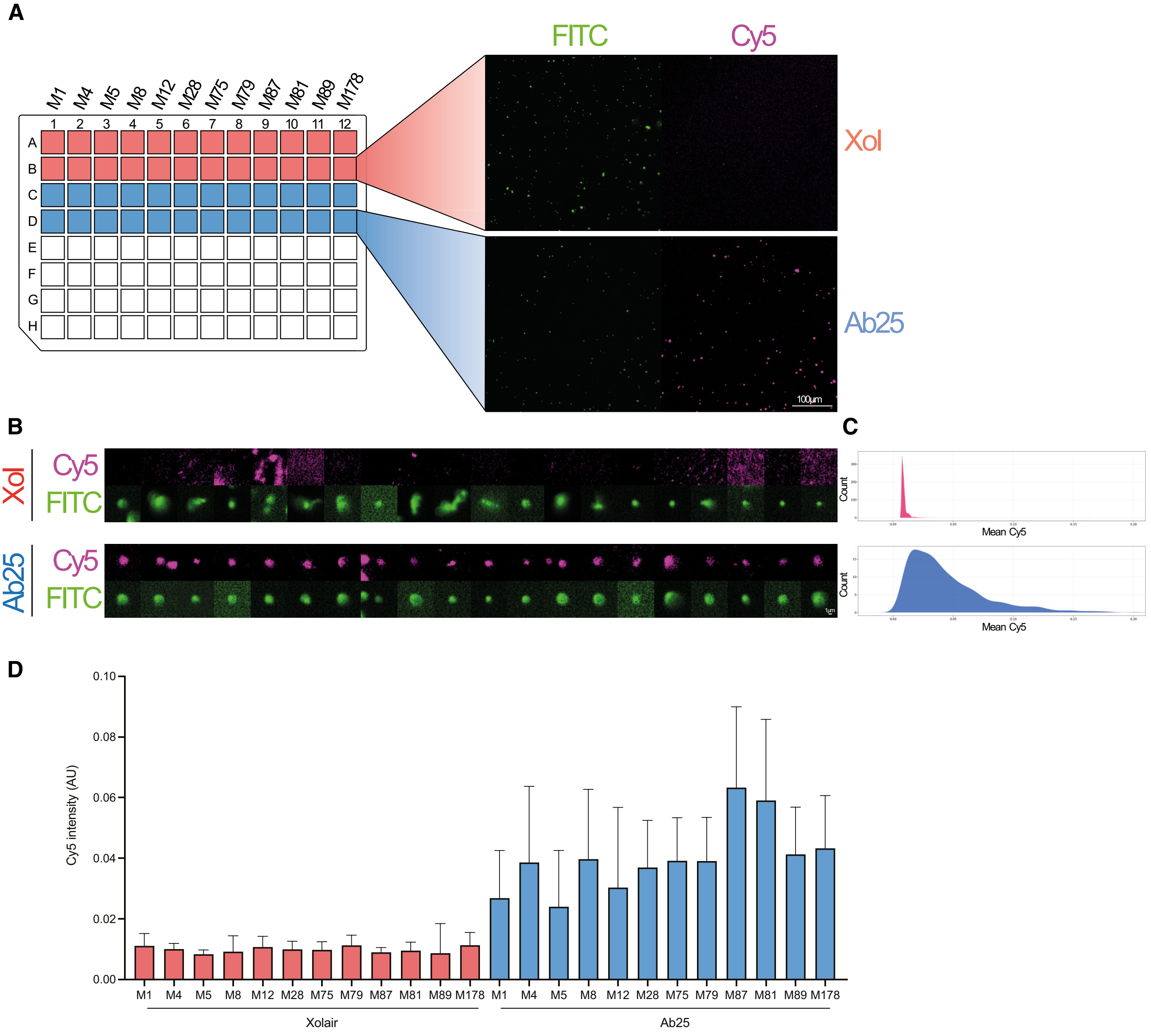

Figure EV5. Cross-strain immunofluorescence.

Heat-killed bacteria were stained with Oregon Green (FITC) and incubated with 10 μg/ml Xolair or Ab25, followed by fluorescent secondary antibody detection (Cy5).

A Images were acquired on a wide-field epifluorescence microscope using a 20× objective (NA = 0.75). The acquisition of images was automated using Nikon JOBs by scanning through a 96-well plate acquiring two images/sample. FITC (bacteria) was segmented by a background segmentation algorithm (> median + 5 std of background signal).

B, C Individual bacteria were labeled, and the overlapping signal in Cy5 was measured. Contrast adjustment is set to the same for all shown images. The images shown are from randomly selected bacteria corresponding to the median data population. As the bacteria were chosen by the software without human intervention, some events could be mildly out-of-focus, explaining the blurry appearance.

D Data is from two separate experiments and with a total of ~ 18,000 bacteria analyzed.

© 2022 The Authors
