# Supplementary material for: A human monoclonal antibody bivalently binding two different epitopes in streptococcal M protein mediates immune function
Source: EMBO Mol Med. 2022 Dec 12;15(2):e16208. doi: 10.15252/emmm.202216208 (PMC9906385; doi:10.15252/emmm.202216208)
Supplement: Supplementary file 6 [file EMMM-15-e16208-s005.pdf]

EMBO Molecular Medicine

A human monoclonal antibody bivalently binding to streptococcal M protein mediates immune function

Wael Bahnan, Lotta Happonen, Hamed Khakzad, Vibha Kumra Ahnlide, Therese de Neergaard, Sebastian Wrighton, Oscar André, Eleni Bratanis, Di Tang, Thomas Hellmark, Lars Björck, Oonagh Shannon, Lars Malmström, Johan Malmstrom, and Pontus Nordenfelt
DOI: 10.15252/emmm.202216208

Corresponding author: Pontus Nordenfelt (pontus.nordenfelt@med.lu.se)

Review Timeline:

Submission Date:	22nd Apr 22
Editorial Decision:	13th May 22
Revision Received:	22nd Sep 22
Editorial Decision:	21st Oct 22
Revision Received:	7th Nov 22
Editorial Decision:	9th Nov 22
Revision Received:	14th Nov 22
Accepted:	16th Nov 22

Editor: Lise Roth

Transaction Report:

(Note: With the exception of the correction of typographical or spelling errors that could be a source of ambiguity, letters and reports are not edited. Depending on transfer agreements, referee reports obtained elsewhere may or may not be included in this compilation. Referee reports are anonymous unless the Referee chooses to sign their reports.)

13th May 2022

Dear Dr. Nordenfelt,

Thank you for the submission of your manuscript to EMBO Molecular Medicine. We have now received feedback from the three reviewers who agreed to evaluate your manuscript. As you will see from the reports below, the referees acknowledge the interest of the study, but also raise a number of concerns that should be adequately addressed in a revised version of this manuscript.

Addressing the reviewers' concerns in full will be necessary for further considering the manuscript in our journal, and acceptance of the manuscript will entail a second round of review. EMBO Molecular Medicine encourages a single round of revision only and therefore, acceptance or rejection of the manuscript will depend on the completeness of your responses included in the next, final version of the manuscript. For this reason, and to save you from any frustrations in the end, I would strongly advise against returning an incomplete revision.

Revised manuscripts should be submitted within three months of a request for revision; they will otherwise be treated as new submissions, except under exceptional circumstances in which a short extension is obtained from the editor.

When submitting your revised manuscript, please carefully review the instructions that follow below. We perform an initial quality control of all revised manuscripts before re-review; failure to include requested items will delay the evaluation of your revision.

We require:

1) A .docx formatted version of the manuscript text (including legends for main figures, EV figures and tables). Please make sure that the changes are highlighted to be clearly visible.

2) Individual production quality figure files as .eps, .tif, .jpg (one file per figure). For guidance, download the 'Figure Guide PDF' (https://www.embopress.org/page/journal/17574684/authorguide#figureformat).

3) A .docx formatted letter INCLUDING the reviewers' reports and your detailed point-by-point responses to their comments. As part of the EMBO Press transparent editorial process, the point-by-point response is part of the Review Process File (RPF), which will be published alongside your paper.

4) A complete author checklist, which you can download from our author guidelines (https://www.embopress.org/page/journal/17574684/authorguide#submissionofrevisions). Please insert information in the checklist that is also reflected in the manuscript. The completed author checklist will also be part of the RPF.

5) It is mandatory to include a 'Data Availability' section after the Materials and Methods. Before submitting your revision, primary datasets produced in this study need to be deposited in an appropriate public database, and the accession numbers and database listed under 'Data Availability'. Please remember to provide a reviewer password if the datasets are not yet public (see https://www.embopress.org/page/journal/17574684/authorguide#dataavailability).
In case you have no data that requires deposition in a public database, please state so in this section. Note that the Data Availability Section is restricted to new primary data that are part of this study.

6) For data quantification: please specify the name of the statistical test used to generate error bars and P values, the number (n) of independent experiments (specify technical or biological replicates) underlying each data point and the test used to calculate p-values in each figure legend. The figure legends should contain a basic description of n, P and the test applied. Graphs must include a description of the bars and the error bars (s.d., s.e.m.).

7) We would also encourage you to include the source data for figure panels that show essential data. Numerical data should be provided as individual .xls or .csv files (including a tab describing the data). For blots or microscopy, uncropped images should be submitted (using a zip archive if multiple images need to be supplied for one panel). Additional information on source data and instruction on how to label the files are available at
.

8) Our journal encourages inclusion of *data citations in the reference list* to directly cite datasets that were re-used and obtained from public databases. Data citations in the article text are distinct from normal bibliographical citations and should directly link to the database records from which the data can be accessed. In the main text, data citations are formatted as follows: "Data ref: Smith et al, 2001" or "Data ref: NCBI Sequence Read Archive PRJNA342805, 2017". In the Reference list, data citations must be labeled with "[DATASET]". A data reference must provide the database name, accession number/identifiers and a resolvable link to the landing page from which the data can be accessed at the end of the reference.

Further instructions are available at .

9) We replaced Supplementary Information with Expanded View (EV) Figures and Tables that are collapsible/expandable online. A maximum of 5 EV Figures can be typeset. EV Figures should be cited as 'Figure EV1, Figure EV2" etc... in the text and their respective legends should be included in the main text after the legends of regular figures.
- For the figures that you do NOT wish to display as Expanded View figures, they should be bundled together with their legends in a single PDF file called *Appendix*, which should start with a short Table of Content. Appendix figures should be referred to in the main text as: "Appendix Figure S1, Appendix Figure S2" etc.
- Additional Tables/Datasets should be labeled and referred to as Table EV1, Dataset EV1, etc. Legends have to be provided in a separate tab in case of .xls files. Alternatively, the legend can be supplied as a separate text file (README) and zipped together with the Table/Dataset file.
See detailed instructions here:
.

10) The paper explained: EMBO Molecular Medicine articles are accompanied by a summary of the articles to emphasize the major findings in the paper and their medical implications for the non-specialist reader. Please provide a draft summary of your article highlighting
- the medical issue you are addressing,
- the results obtained and
- their clinical impact.
This may be edited to ensure that readers understand the significance and context of the research. Please refer to any of our published articles for an example.

11) For more information: There is space at the end of each article to list relevant web links for further consultation by our readers. Could you identify some relevant ones and provide such information as well? Some examples are patient associations, relevant databases, OMIM/proteins/genes links, author's websites, etc...

12) Conflict of interest: We updated our journal's competing interests policy in January 2022 and request authors to consider both actual and perceived competing interests. Please review the policy https://www.embopress.org/competing-interests and update your competing interests if necessary.

13) Every published paper now includes a 'Synopsis' to further enhance discoverability. Synopses are displayed on the journal webpage and are freely accessible to all readers. They include a short stand first (maximum of 300 characters, including space) as well as 2-5 one-sentences bullet points that summarizes the paper. Please write the bullet points to summarize the key NEW findings. They should be designed to be complementary to the abstract - i.e. not repeat the same text. We encourage inclusion of key acronyms and quantitative information (maximum of 30 words / bullet point). Please use the passive voice. Please attach these in a separate file or send them by email, we will incorporate them accordingly.
Please also suggest a striking image or visual abstract to illustrate your article as a PNG file 550 px wide x 300-600 px high.

14) As part of the EMBO Publications transparent editorial process initiative (see our Editorial at http://embomolmed.embopress.org/content/2/9/329), EMBO Molecular Medicine will publish online a Review Process File (RPF) to accompany accepted manuscripts.

In the event of acceptance, this file will be published in conjunction with your paper and will include the anonymous referee reports, your point-by-point response and all pertinent correspondence relating to the manuscript. Let us know whether you agree with the publication of the RPF and as here, if you want to remove or not any figures from it prior to publication.
Please note that the Authors checklist will be published at the end of the RPF.

EMBO Molecular Medicine has a "scooping protection" policy, whereby similar findings that are published by others during review or revision are not a criterion for rejection. Should you decide to submit a revised version, I do ask that you get in touch after three months if you have not completed it, to update us on the status.

I look forward to receiving your revised manuscript.

Yours sincerely,

Lise Roth

Lise Roth, PhD
Senior Editor
EMBO Molecular Medicine

***** Reviewer's comments *****

Referee #1 (Remarks for Author):

This study by Bahnan et al examines the interaction of antibodies with M proteins from GAS. Using B cells from an immune individual, they identified three antibodies specific to the M protein. They characterize the ability of these Abs to bind the bacterial surface, promote agglutination, activate macrophages and induce opsonophagocytosis. They identify Ab25 as being the more efficient at several activities, reacts with M proteins across several strains and protects hosts in an in vivo model. In comparing binding regions, they find compelling evidence using TX-MS that the Fab region of Ab25 can interact with two distinct epitopes on the M protein. Using assays with single Fab or F(ab')2 they demonstrate that the latter has better affinity to the M protein and is required for efficient bacterial binding.

Major comments
This study is of high relevance and provides important contributions to the field in terms of a potentially novel way of Ab/epitope interaction as well as protective Abs against GAS. The assays are very well controlled and the manuscript is easy to follow. A few points need clarification:

1) Clarify how F(ab')2 fragments were generated in Figure 1. Was fragmentation verified via WBs?
2) Figure 2 D is missing a few labels. Time for lower figures and what strain was used in each panel
3) Assessing bacterial killing by immune cells following opsonophagocytosis elicited by Ab 25 vs other Abs would strengthen the findings
4) IRB approvals/information and donor consent pertaining data from donors is missing
5) A discussion of whether both predicted epitopes of the M protein would be surface exposed when the protein is on the bacterial surface is warranted

Referee #2 (Comments on Novelty/Model System for Author):

This manuscript is of high technical quality, the findings are novel, and the model systems seem fine. The medical impact is difficult to assess as the authors do not show whether their interesting findings are the result of a rare accident of nature or a fundamental alternative mechanism of antibody/pathogen binding.

Referee #2 (Remarks for Author):

This manuscript describes high quality research detailing a provocative finding of the authors while they were characterizing an anti-streptococcal mAb obtained from a recovered strep patient. This mAb, Ab25, quite surprisingly binds two different epitopes at nearby sites on Group A streptococcal (GAS) M protein such that the two Fab components of the mAb bind simultaneously to each of the different sites. This novel binding property is called a 'dual-Fab' binding phenomenon. Apparently because of this property, the mAb was shown to possess enhanced anti-infective properties and surprisingly broad variant specificity, likely as a result of the synergy from the simultaneous binding of the two Fabs. The data appears robust and generally well-presented. What remains unclear, though, is whether this very unusual and novel property is a rare accident, or an important mechanism commonly employed by patients successfully recovering from GAS infections. Showing that this 'dual-Fab' is a general anti-infective mechanism for antibodies would substantially increase the impact of this paper. In addition, there are areas that would benefit from modification and clarification.

Specific comments:
1. Authors report the identification of 10 antibody pairs following single cell PCR from the IgG+, M+ cells. To better evaluate this critical discovery process, it would be of interest to know how many Ab pairs in total were analyzed, how often identical or related Ab pairs were also identified, and most importantly, what criteria was employed that resulted in the selection of the three Ab pairs used for the studies reported in the manuscript.
2. More information should be provided as to mAb expression. Clearly the Vh and Vl Ab pairs were introduced into the expression vector and produced as mAbs, but no information was offered as to the isotype or the source species of the Fc domain encoded by the mAb expression vector.
3. There is no referencing of Supp. Fig 2A in the Results and this figure has a labeling flaw needing correction.
4. The four panels in Fig. 2D need to be labeled and more details provided in the legend.
5. Supp. Fig. 4B is not referenced in the Results and association % is not explained in the legend.
6. Supp. Fig. 6A shows whole blood phagocytosis images of four strains of GAS expressing GFP. Please explain the basis for selecting these strain and how closely related they are.
7. Fig. 4A shows scatter plot data on mouse infection data. The results show that most mice appeared to be protected with Ab25 based on bacterial burden in different tissues and plasma cytokine measurements. A few mice though were outliers in each

assay. It would be useful for the authors to indicate, if information is available, whether the same mice were outliers in each assay, as might be expected.
8. Fig. 5H shows the estimated height of various M-protein binding mAbs relative to the cell wall. A comparison is made to Xolair which binds through the Fc domain. No data was apparently provided as to the significance of differences observed. Also, can the authors eliminate or discount contributions to these findings from mAbs binding to the Fc-binding site on M-protein?
9. Fig. 6A shows competition studies for Xolair Fc-binding by various anti-M protein mAbs and controls. Since Ab32 and Ab49 bind at much higher affinity than Xolair (Fig. 1E), and apparently bind at a nearby sites based on cross-linking analysis (Fig. 5E, Supp. Table 1) and their binding site height (Fig. 5H), it seems surprising that their binding does not sterically compete with Xolair binding. Perhaps the authors could comment on this.
10. It also seems that any antibody with an appropriate Fc domain should equally compete for Fc- binding by Xolair in Fig. 6A. Since the mAbs are 10-fold lower in concentration explains the low competition of these mAbs with Xolair in the Fig. 6A. More surprising is that an equivalent amount of IVIG (100 ug) did not noticeably compete for Xolair binding at the Fc-binding site. Please explain or clarify if possible.
11. The authors hypothesize that it is the 'dual-Fab' feature that is critical for the enhanced binding and anti-infective properties of Ab25. Yet in Fig. 6G, Ab25 binding to bacterial strain M12 occurred even with individual Fabs. Thus, Ab25 does not require dual Fab for binding, and still promotes M12 phagocytosis (Fig. 3F). This exception seems worth further analysis. It seems important to know if binding to M12 occurs because the Ab25 Fab has particularly high affinity to one site of the M protein epitopes on M12. This could explain the lack of a dual-Fab requirement for binding to this strain even if dual site binding occurs.
12. Fig. 6E may be the most provocative data in this paper as it suggests that the 'dual-Fab' mechanism may be quite widely employed in response to SAG infections. On the other hand, 'dual Fabs' must consist of Fabs that have acquired the ability to bind to two epitopes which are also positioned nearby. Because of these unusual and stringent requirements, it would seem that acquisition of this ability should be extremely rare. Confirming the dual-Fab mechanism as a common occurrence through additional studies would thus substantially strengthen this paper. Without this data, it seems more should be included in the discussion as to the potential general nature of this mechanism vs a rare quirk of nature.
13. Quite a few typos were noted that should be corrected with a spell-check.

Referee #3 (Comments on Novelty/Model System for Author):

In this study by Bahnan et al, entitled "A human monoclonal antibody bivalently binding two different epitopes in streptococcal M protein protects against infection", the authors generated several human monoclonal antibodies (mAbs) from the peripheral blood B cells of a convalescent individual. A meticulous characterization of these mAbs lead to the identification of a unique mAb, the Ab25, whose two Fab arms bind to different epitopes positioned at "arms length" of the same M-protein (a well-known virulence determinant of Group A streptococcus). Importantly, the Ab25 serves as a therapeutic mAb in a challenge model in vivo. To make this work unique, additional experiments are needed.

For example, the Ab49 recognizes similar emm serotypes as the Ab25 (Fig. 1F) and shares the site 1 of the Ab25 for its occupancy. The authors should consider the protection mediated by Ab 49 as a comparison to establish that the "bivalent dual-Fab cis mode" binding may be unique in its ability to protect in vivo. Such experiment will not only justify the title and validates the central message of this manuscript. Moreover, it will also support the speculations made in the last two paragraphs of the discussion related to "bivalent dual-Fab cis mode".

Referee #3 (Remarks for Author):

In this study by Bahnan et al, entitled "A human monoclonal antibody bivalently binding two different epitopes in streptococcal M protein protects against infection", the authors generated several human monoclonal antibodies (mAbs) from the peripheral blood B cells of a convalescent individual. A meticulous characterization of these mAbs lead to the identification of a unique mAb, the Ab25, whose two Fab arms bind to different epitopes positioned at "arms length" of the same M-protein (a well-known virulence determinant of Group A streptococcus). Importantly, the Ab25 serves as a therapeutic mAb in a challenge model in vivo. To make this work unique, additional experiments are needed.

Major comments.

1. The Ab49 recognizes similar emm serotypes as the Ab25 (Fig. 1F) and shares the site 1 of the Ab25 for its occupancy. The authors should consider the protection mediated by Ab 49 as a comparison to establish that the "bivalent dual-Fab cis mode" binding may be unique in its ability to protect in vivo. Such experiment will not only justify the title and validates the central message of this manuscript. Moreover, it will also support the speculations made in the last two paragraphs of the discussion related to "bivalent dual-Fab cis mode".

2. Female mice are relatively resistant to S. pyogenes infection compared to male mice. Therefore, it is very important to consider sex as a biological variable. Male mice should be included.

3. Since the "bivalent dual-Fab cis mode" binding can be maintained by Ab25 F(ab)2, even in the presence of IdeS (an IgG cleaving protease) expressed by the GAS, it is likely that Ab25 F(ab)2 can maintain certain effector functions in vivo. Did the authors consider the ability of the Ab25 F(ab)2 protect mice against GAS challenge as in Fig 4A?

4. What is the IgG subclass identity of these monoclonal antibodies e.g., Ab 25, 32 & 49 etc)? So that the effector functions can be predictable in humans.

Minor comment.

1. Fig 1A, B & C, the y axis should be labeled AF-647 not APC, since APC is not the actual fluorescent dye used.

2. Fig 6B, the green bar should be labelled untreated not Xolair.

3. Fig 6C should be in the supplementary data or can be deleted.

4. Fig 6D, the y axis should be labeled AF-647 not APC.

Dear editor and reviewers,

We thank the reviewers for their thoughtful comments. These helped us in clarifying some ambiguities in the text and figures, as well as correcting some errors. Based on the suggestion from one of the reviewers, we also attempted to do ambitious in vivo experiments that, despite our best efforts, did not yield any new information. This comment also made it clear that our title could be interpreted as if we had proven that dual-Fab was the protective mechanism in vivo. Our intention with the original animal experiments was to determine if Ab25 was a candidate for antibody mediated therapy, knowing how difficult it would be to determine the mechanism of protection in vivo since susceptibility in invasive bacterial infection models is a combination of bacteria mediated invasion and inflammatory damage. We, therefore, suggest a slightly modified title, replacing "protects against infection" with "mediates immune function", highlighting what we have clearly shown: that the bivalent dual-Fab Ab25 binding mediates immune function compared to monovalent interaction at the same site. We believe this and the other changes throughout the manuscript makes the contribution to the field clearer. Please see our detailed responses to all the reviewers' comments below.

Best regards,
Pontus Nordenfelt on behalf of the authors

****** Reviewer's comments ******

Referee #1 (Remarks for Author):

This study by Bahnan et al examines the interaction of antibodies with M proteins from GAS. Using B cells from an immune individual, they identified three antibodies specific to the M protein. They characterize the ability of these Abs to bind the bacterial surface, promote agglutination, activate macrophages and induce opsonophagocytosis. They identify Ab25 as being the more efficient at several activities, reacts with M proteins across several strains and protects hosts in an in vivo model. In comparing binding regions, they find compelling evidence using TX-MS that the Fab region of Ab25 can interact with two distinct epitopes on the M protein. Using assays with single Fab or F(ab')2 they demonstrate that the latter has better affinity to the M protein and is required for efficient bacterial binding.

Major comments
This study is of high relevance and provides important contributions to the field in terms of a potentially novel way of Ab/epitope interaction as well as protective Abs against GAS. The assays are very well controlled and the manuscript is easy to follow. A few points need clarification:

1) Clarify how F(ab')2 fragments were generated in Figure 1. Was fragmentation verified via WBs?
The generation of F(ab')2 fragments throughout the manuscript was performed the same way as in Fig. 6C, via cleavage using the IdeS-enzyme. The activity of the enzyme and the complete cleavage of whole plasma IgG has been described for this same batch before (Happonen et al., 2019). As it is an established method and enzyme that we routinely see efficient F(ab')2 fragment generation with, we did not find it necessary to repeat the WB analysis for digestion quality control for every experiment. We always confirm that new batches of the IdeS enzyme are

functional. We have further clarified this in the figure legend, see lines 1046-47.

2) Figure 2 D is missing a few labels. Time for lower figures and what strain was used in each panel.

We thank the reviewer for this comment. We have now fixed the time label and added the strain information to the figure legend, see line 1064-1065.

3) Assessing bacterial killing by immune cells following opsonophagocytosis elicited by Ab 25 vs other Abs would strengthen the findings.

Knowing that this is a common assay in the field, despite the clear issues with reproducibility with GAS, we have spent several months trying to attain reproducible results. Killing assays with GAS has been very problematic for us and others in the field, most likely due to the strong aggregation ability of GAS, introducing variability to CFU counts. We have attempted several variations of the Lancefield assay (Reglinski, 2020) but we have never seen robust results. We tried different cell lines (THP1, HL60) and primary cells (whole blood and purified neutrophils), without success. Our experience has been consistent with that of others and this has been published previously (Salehi et al., 2018). Here we show the data we got from the killing assay attempts. As one can easily see, there is no killing advantage by using high dose IVIG (gold standard) compared to untreated control. While the Ab25 data indicate that there might be some protection also in this assay, we simply do not trust this assay when the controls are not stable. For that reason, we shifted to the MOP50-based phagocytosis assays (Fig. 3) to assess opsonic ability of the antibodies, which are more sensitive and consistent.

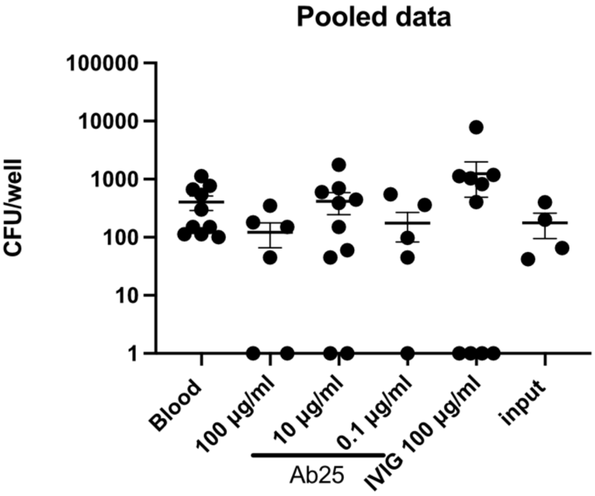

Blood killing assays based on the Lancefield assay published in Reglisnki (2020). Brielfly, 300 µl Blood was inoculated with 1000 CFU (of SF370, M1) that had been previously opsonized with Ab25 (37°C, 30 minutes). Three hours post infection, aliquots of the blood were plated onto blood agar plates and the colonies were enumerated the following day. Every data point on the graph refers to an experimental replicate. A total of 4 independent experiments were performed and the data was pooled.

4) IRB approvals/information and donor consent pertaining data from donors is missing

The approval from the ethics committee can be found in the methods section at lines 449-451. We now also added that the donor gave written consent.

5) A discussion of whether both predicted epitopes of the M protein would be surface exposed when the protein is on the bacterial surface is warranted.

The M protein is anchored in the cell wall via an LPXTG motif localized downstream of the D domain. Hence, the M protein would be displayed on the bacterial surface in the following manner: Cell wall- D- C- S- B-A. As the antibody contact epitopes are in the B/C domains, we assume that they will be presented on the bacterial surface since M protein is typically extending out from the surface. Supporting this, a previous paper with several of the current authors (Nordenfelt et al., 2012) show the location of the S epitope when antibodies are bound via Fc interaction using EM images. In these images it is evident that epitopes located closely to the S region would be exposed at the bacterial surface. We have added this information to the discussion, see lines 343-346.

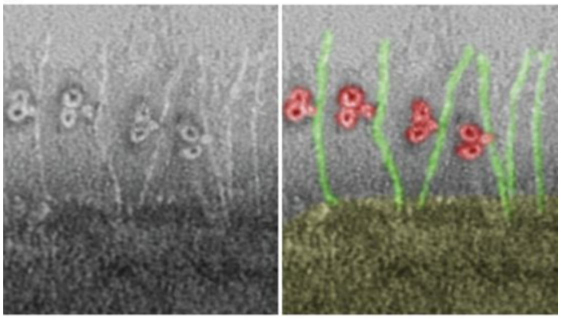

Negative staining EM was used to visualize the localization and orientation of IgG bound to the bacterial surface. High magnification shows single IgG molecules bound or via Fc in saliva. Pseudo-color variants are shown to better visualize bound IgG (red) and M proteins (green).
From Nordenfelt et al, 2012. J Exp. Med.

Referee #2 (Comments on Novelty/Model System for Author):

This manuscript is of high technical quality, the findings are novel, and the model systems seem fine. The medical impact is difficult to assess as the authors do not show whether their interesting findings are the result of a rare accident of nature or a fundamental alternative mechanism of antibody/pathogen binding.

Referee #2 (Remarks for Author):

This manuscript describes high quality research detailing a provocative finding of the authors while they were characterizing an anti-streptococcal mAb obtained from a recovered strep patient. This mAb, Ab25, quite surprisingly binds two different epitopes at nearby sites on Group A streptococcal (GAS) M protein such that the two Fab components of the mAb bind simultaneously to each of the different sites. This novel binding property is called a 'dual-Fab' binding phenomenon. Apparently because of this property, the mAb was shown to possess enhanced anti-infective properties and surprisingly broad variant specificity, likely as a result of the synergy from the simultaneous binding of the two Fabs. The data appears robust and generally well-presented. What remains unclear, though, is whether this very unusual and novel property is a rare accident, or an important mechanism commonly employed by patients successfully recovering from GAS infections. Showing that this 'dual-Fab' is a general anti-infective mechanism for antibodies would substantially increase the impact of this paper. In addition, there are areas that would benefit from modification and clarification.

Specific comments:
1. Authors report the identification of 10 antibody pairs following single cell PCR from the IgG+, M+ cells. To better evaluate this critical discovery process, it would be of interest to know how many Ab pairs in total were analyzed, how often identical or related Ab pairs were also identified, and most importantly, what criteria was employed that resulted in the selection of the three Ab pairs used for the studies reported in the manuscript.

We thank the reviewer for their comment. Our input material was 100 B cells. From those, we successfully cloned 10 antibodies and of those, 4 were reactive to the M protein. This information is present in the methods (line 496) and results section (line 106-108). Since then, we have modified our methods and will be using updated primers which allow higher PCR success for future studies.
As to the criteria of why the 3 antibodies were used instead of the 4, we had preliminary data that the 4th antibody (Ab26) also showed dual Fab binding, albeit to a different site. We were, however, not able to generate structural TX-MS data to verify this, and hence Ab26 was not discussed further in the paper.

2. More information should be provided as to mAb expression. Clearly the Vh and Vl Ab pairs were introduced into the expression vector and produced as mAbs, but no information was offered as to the isotype or the source species of the Fc domain encoded by the mAb expression vector.
We thank the reviewer for this comment. This has now been corrected in lines 481-484.

3. There is no referencing of Supp. Fig 2A in the Results and this figure has a labeling flaw needing correction.
This has now been fixed (line 145). Time labels have now been added to panel 2B in Supp. Fig 2.

4. The four panels in Fig. 2D need to be labeled and more details provided in the legend.
The time scale and strain used have now been added to the figure and legend (see line 1064-1066).

5. Supp. Fig. 4B is not referenced in the Results and association % is not explained in the legend.
For the reference of the figure, please see line 183. The legend has also been modified see Fig. EV4.

6. Supp. Fig. 6A shows whole blood phagocytosis images of four strains of GAS expressing GFP. Please explain the basis for selecting these strain and how closely related they are.
The strains were selected based on that we could make them GFP-positive (successful transformation) and that they also cover a range of emm strains. Compared to emm79 at the level of emm sequence homology, emm1 is most similar, followed by emm 12 and 5. This data is found in Fig 1F. We have added the selection basis to lines 748-750.

7. Fig. 4A shows scatter plot data on mouse infection data. The results show that most mice appeared to be protected with Ab25 based on bacterial burden in different tissues and plasma cytokine measurements. A few mice though were outliers in each assay. It would be useful for the authors to indicate, if information is available, whether the same mice were outliers in each assay, as might be expected.
In our mouse model of systemic infection, bacteria are administered subcutaneously in the scruff of the neck from which they disseminate through the blood to organs. The ability to disseminate is a multifactorial combination of bacterial virulence factors and host immune response, therefore there is always a heterogenous interindividual response. The CFUs in different tissue are correlated to one another and to the cytokine levels, and as such the same mice are indeed the outliers in all assays. This is now highlighted in the text in lines 232-233.

8. Fig. 5H shows the estimated height of various M-protein binding mAbs relative to the cell

wall. A comparison is made to Xolair which binds through the Fc domain. No data was apparently provided as to the significance of differences observed. Also, can the authors eliminate or discount contributions to these findings from mAbs binding to the Fc-binding site on M-protein?

The data presented in Fig. 5H is free of interference from Fc binding, as the antibodies were IdeS-cleaved to generate F(ab')2 fragments before being used to stain the bacteria. The secondary antibody used to visualize the primary binding was a fluorescent Fab anti-Fab antibody. Only for Xolair, however, Anti-Fc fluorescent secondary antibodies were used as they do not have Fab-based binding. Using a multiple comparison Kruskal-Wallis test, no significant differences in height were observed, indicating that they bind close to each other. Please refer to the methods section on SIM imaging. We have also made the fact clear that we use F(ab')2 fragments in lines 612-614, and mentioned the statistical test in the figure legend (lines 1137-1139).

9. Fig. 6A shows competition studies for Xolair Fc-binding by various anti-M protein mAbs and controls. Since Ab32 and Ab49 bind at much higher affinity than Xolair (Fig. 1E), and apparently bind at a nearby sites based on cross-linking analysis (Fig. 5E, Supp. Table 1) and their binding site height (Fig. 5H), it seems surprising that their binding does not sterically compete with Xolair binding. Perhaps the authors could comment on this.

The steric hindrance seen with Ab25 is not (as we believe) due to spatial adjacency of binding epitopes. Since Ab25 spans the B/S/C domains of the M protein, it can effectively create a bridge that precludes Fc binding. In fact, the experiment presented in Fig 6C was a specific test of the dual-Fab binding hypothesis that came from the structural modeling data. We have made this clearer in line 313-315.

10. It also seems that any antibody with an appropriate Fc domain should equally compete for Fc- binding by Xolair in Fig. 6A. Since the mAbs are 10-fold lower in concentration explains the low competition of these mAbs with Xolair in the Fig. 6A. More surprising is that an equivalent amount of IVIG (100 ug) did not noticeably compete for Xolair binding at the Fc-binding site. Please explain or clarify if possible.

This point has two questions in it. First, The Fc binding to the M protein is of very low affinity (see Fig. 1E of the manuscript, (Ahnlide et al., 2021) and (Akesson et al.). It, therefore, requires higher concentrations of antibodies to get Fc binding, compared to regular Fab binding. This is why we used 10-fold more Xolair than Ab25/32/49. Second, on the topic of IVIG; part of the discoveries in this work was that these dual-Fab antibodies are enriched in convalescing patient blood but not in healthy donor blood. This, we think, explains why there is very low incidence of dual-Fab antibodies in IVIG. We have made this clearer in lines 298-300.

11. The authors hypothesize that it is the 'dual-Fab' feature that is critical for the enhanced binding and anti-infective properties of Ab25. Yet in Fig. 6G, Ab25 binding to bacterial strain M12 occurred even with individual Fabs. Thus, Ab25 does not require dual Fab for binding, and still promotes M12 phagocytosis (Fig. 3F). This exception seems worth further analysis. It seems important to know if binding to M12 occurs because the Ab25 Fab has particularly high affinity to one site of the M protein epitopes on M12. This could explain the lack of a dual-Fab requirement for binding to this strain even if dual site binding occurs.

We value the reviewer's comment. Our hypothesis is that dual-Fab and single Fab binding can occur interchangeably with M12, where dual-Fab is what mediates function. We do elude to this in the results section, that we cannot identify the mode of action of Ab25 with M12, and whether it is functioning via monovalent or bivalent mode. We have attempted to clarify the conclusions from this experiment in the lines 333-335.

12. Fig. 6E may be the most provocative data in this paper as it suggests that the 'dual-Fab' mechanism may be quite widely employed in response to SAG infections. On the other hand, 'dual Fabs' must consist of Fabs that have acquired the ability to bind to two epitopes which are also positioned nearby. Because of these unusual and stringent requirements, it would seem that acquisition of this ability should be extremely rare. Confirming the dual-Fab mechanism as a common occurrence through additional studies would thus substantially strengthen this paper. Without this data, it seems more should be included in the discussion as to the potential general nature of this mechanism vs a rare quirk of nature.

We agree with the reviewer. We have addressed this in the discussion. Please see lines 424-429.

13. Quite a few typos were noted that should be corrected with a spell-check.

We have identified a number of typos that have now been fixed.

Referee #3 (Comments on Novelty/Model System for Author):

In this study by Bahnan et al, entitled "A human monoclonal antibody bivalently binding two different epitopes in streptococcal M protein protects against infection", the authors generated several human monoclonal antibodies (mAbs) from the peripheral blood B cells of a convalescent individual. A meticulous characterization of these mAbs lead to the identification of a unique mAb, the Ab25, whose two Fab arms bind to different epitopes positioned at "arms length" of the same M-protein (a well-known virulence determinant of Group A streptococcus). Importantly, the Ab25 serves as a therapeutic mAb in a challenge model in vivo. To make this work unique, additional experiments are needed.

For example, the Ab49 recognizes similar emm serotypes as the Ab25 (Fig. 1F) and shares the site 1 of the Ab25 for its occupancy. The authors should consider the protection mediated by Ab 49 as a comparison to establish that the "bivalent dual-Fab cis mode" binding may be unique in its ability to protect in vivo. Such experiment will not only justify the title and validates the central message of this manuscript. Moreover, it will also support the speculations made in the last two paragraphs of the discussion related to "bivalent dual-Fab cis mode".

Referee #3 (Remarks for Author):

In this study by Bahnan et al, entitled "A human monoclonal antibody bivalently binding two different epitopes in streptococcal M protein protects against infection", the authors generated several human monoclonal antibodies (mAbs) from the peripheral blood B cells of a convalescent individual. A meticulous characterization of these mAbs lead to the identification of a unique mAb, the Ab25, whose two Fab arms bind to different epitopes positioned at "arms length" of the same M-protein (a well-known virulence determinant of Group A streptococcus).

Importantly, the Ab25 serves as a therapeutic mAb in a challenge model in vivo. To make this work unique, additional experiments are needed.

Major comments.

1. The Ab49 recognizes similar emm serotypes as the Ab25 (Fig. 1F) and shares the site 1 of the Ab25 for its occupancy. The authors should consider the protection mediated by Ab 49 as a comparison to establish that the "bivalent dual-Fab cis mode" binding may be unique in its ability to protect in vivo. Such experiment will not only justify the title and validates the central message of this manuscript. Moreover, it will also support the speculations made in the last two paragraphs of the discussion related to "bivalent dual-Fab cis mode".

We thank the reviewer for this comment. We would like to discuss 3 points relating to their concerns:

A) We understand the reviewer's concerns, especially about the speculative comments we make in relation to in vivo protection. For that purpose, we have corrected the interpretation and implications of the in vivo results in the manuscript since a direct comparison of Ab25 and Ab49 is lacking. The animal infection was not designed to give mechanistic insights into in vivo efficacy of Ab25 but to determine whether Ab25 could also be effective in a multifactorial in vivo model and represent a potential proof of principle for future antibody mediated therapeutic intervention (lines 224-227). We highlight in the last sentence of our discussion (lines 439-441) where we discuss that more in vivo experiments are required to get deeper understanding of the mechanism and in vivo role of dual Fab binding. See also point C below regarding our proposed title change.

B) The animal model used in the paper is a systemic infection model where GAS bacteria are administered by subcutaneous injection in the scruff of the neck from which bacteria can invade and spread to cause bacteremia and dissemination to distant organs. The susceptibility to systemic infection is multifactorial and dependent on bacterial virulence, host genetics and immune status (Aziz et al., 2007). The bacterial inoculum is grown fresh for each experiment and the same CFUs are administered, however interindividual responses are always observed. This results in the heterogenous spread of bacteria load and cytokine responses in individual mice. In experimental cohorts where no or little bacterial dissemination occurs no therapeutic effect can be investigated. In 2018 when the animal experiments presented in Fig. 4 were performed, 3 experiments were performed, one of which did not yield bacterial dissemination or productive infection in the mice. The results from the two systemic infection models are reported in the manuscript. Based on the proposal of the reviewer, we performed the scruff infection again twice to compare Ab49 and Ab25. Unfortunately, systemic infection did not occur in either model this time and we are reluctant to repeat these experiments due to ethical constraints. Our lab has recently developed a flank infection model (Naegeli et al., 2019), which demonstrates a more robust dissemination of bacteria from this contained site and also has the benefit that a biopsy of the local infection can also be removed to quantity CFU at the local site of infection. This model will be used in future work to determine the niche at which IVIG, Ab25 or other candidate antibodies mediate protection and investigate the mechanism involved.

C) What our other combined experimental evidence supports is that the bivalent interaction (dual-Fab) of Ab25 does mediate an immune function. This is demonstrated by the many

comparisons to the monovalently interacting Ab49 throughout the manuscript, where Ab49 consistently does not provide a significant immunological difference to our IgG1 control (Xolair), whereas Ab25 does. Ab25 also provides in vivo protection, but as the reviewer alludes to, we cannot be sure through which mechanism, and unfortunately, the model we have used is not robust enough to provide this answer. We would like to clarify this by changing the title to: from 'A human monoclonal antibody bivalently binding two different epitopes in streptococcal M protein protects against infection' to 'A human monoclonal antibody bivalently binding two different epitopes in streptococcal M protein mediates immune function'.

2. Female mice are relatively resistant to S. pyogenes infection compared to male mice. Therefore, it is very important to consider sex as a biological variable. Male mice should be included.

The susceptibility to systemic GAS infection is multifactorial and dependent on bacterial virulence, host genetics and immune status (Aziz et al., 2007). In certain mouse backgrounds we certainly agree that male mice are more susceptible. In our C57/BL6 background and with this GAS strain we have previously demonstrated equivalent susceptibility to infection for males and females (Shannon et al., 2010). In our experiments, we are comparing females to females, in order to decrease the interindividual variation and focus on determining an antibody mediated effect. Sex is indeed an important variable for future work, but we do not address it in this particular proof of principle case.

3. Since the "bivalent dual-Fab cis mode" binding can be maintained by Ab25 F(ab)2, even in the presence of IdeS (an IgG cleaving protease) expressed by the GAS, it is likely that Ab25 F(ab)2 can maintain certain effector functions in vivo. Did the authors consider the ability of the Ab25 F(ab)2 protect mice against GAS challenge as in Fig 4A?

We believe that the protective effect seen with Ab25 are due to its effector immune function, and not aggregation or adhesion neutralization. Ab32 and 49 both aggregate bacteria (Supp Fig. EV2B) but have no in vitro immune function. As we have discussed in the first point of our response, we performed the animal experiments with the sole purpose of identifying any protective effect of Ab25 in vivo. It is therefore unlikely that we would see any F(ab')2 mediated protective effects in an in vivo experiment.

4. What is the IgG subclass identity of these monoclonal antibodies e.g., Ab 25, 32 & 49 etc)? So that the effector functions can be predictable in humans.

All the antibodies were expressed in an IgG1 background. At the time of the experiments, the methodology we used did not yield information on original (in vivo) subclass. We have made this clear in lines 481-484.

Minor comment.
1. Fig 1A, B & C, the y axis should be labeled AF-647 not APC, since APC is not the actual fluorescent dye used.

We have changed that.

2. Fig 6B, the green bar should be labelled untreated not Xolair.

We have changed that.

3. Fig 6C should be in the supplementary data or can be deleted.

We have changed that.

4. Fig 6D, the y axis should be labeled AF-647 not APC.

We have changed that.

References:

Ahnlide, V.K., Neergaard, T. de, Sundwall, M., Ambjörnsson, T., and Nordenfelt, P. (2021). A Predictive Model of Antibody Binding in the Presence of IgG-Interacting Bacterial Surface Proteins. Front Immunol *12*, 629103. https://doi.org/10.3389/fimmu.2021.629103.

Aziz, R.K., Kansal, R., Abdeltawab, N.F., Rowe, S.L., Su, Y., Carrigan, D., Nooh, M.M., Attia, R.R., Brannen, C., Gardner, L.A., et al. (2007). Susceptibility to severe streptococcal sepsis: use of a large set of isogenic mouse lines to study genetic and environmental factors. Genes Immun *8*, 404–415. https://doi.org/10.1038/sj.gene.6364402.

Happonen, L., Hauri, S., Birkedal, G.S., Karlsson, C., Neergaard, T. de, Khakzad, H., Nordenfelt, P., Wikström, M., Wisniewska, M., Björck, L., et al. (2019). A quantitative Streptococcus pyogenes–human protein–protein interaction map reveals localization of opsonizing antibodies. Nat Commun *10*, 2727. https://doi.org/10.1038/s41467-019-10583-5.

Naegeli, A., Bratanis, E., Karlsson, C., Shannon, O., Kalluru, R., Linder, A., Malmström, J., and Collin, M. (2019). Streptococcus pyogenes evades adaptive immunity through specific IgG glycan hydrolysis. J Exp Med *216*, 1615–1629. https://doi.org/10.1084/jem.20190293.

Nordenfelt, P., Waldemarson, S., Linder, A., Mörgelin, M., Karlsson, C., Malmström, J., and Björck, L. (2012). Antibody orientation at bacterial surfaces is related to invasive infection. J Exp Medicine *209*, 2367–2381. https://doi.org/10.1084/jem.20120325.

Reglinski, M. (2020). Group A Streptococcus, Methods and Protocols. Methods Mol Biology *2136*, 317–322. https://doi.org/10.1007/978-1-0716-0467-0_25.

Salehi, S., Hohn, C.M., Penfound, T.A., and Dale, J.B. (2018). Development of an Opsonophagocytic Killing Assay Using HL-60 Cells for Detection of Functional Antibodies against Streptococcus pyogenes. Msphere *3*, e00617-18. https://doi.org/10.1128/msphere.00617-18.

Shannon, O., Rydengård, V., Schmidtchen, A., Mörgelin, M., Alm, P., Sørensen, O.E., and Björck, L. (2010). Histidine-rich glycoprotein promotes bacterial entrapment in clots and decreases mortality in a mouse model of sepsis. Blood *116*, 2365–2372. https://doi.org/10.1182/blood-2010-02-271858.

Dear Dr. Nordenfelt,

I am getting back to you regarding your revised manuscript.
In addition to the points mentioned below, please also deposit your NGS data in a public database. It is mandatory to deposit primary datasets in an appropriate public database, and to list the accession numbers and database under 'Data Availability'. Please also include the Data EV1 and EV2 in the appendix.

Please let me know if you have any question,

With kind regards,

Lise Roth

21st Oct 2022

Dear Dr. Nordenfelt,

Thank you for the submission of your manuscript to EMBO Molecular Medicine. We have now received the reports from referees #2 and #3 who re-reviewed your manuscript. As you will see below, they are now supportive of publication, and I am therefore pleased to inform you that we will be able to accept your manuscript once the following editorial points will be addressed:

1/ Manuscript text:
- Please address the queries from our data editors in the Data edited file in track changes mode and use this file for any further modification.
- Please suggest up to 5 keywords.
- Please add the heading "Abstract" to the first paragraph.
- Material and methods:
o The table at the beginning of the Methods section should be removed from the manuscript and uploaded as a separate file.
o Antibody dilutions should be provided.
o Human samples: Include a statement that the patients provided written informed consent and that the experiments conformed to the principles set out in the WMA Declaration of Helsinki and the Department of Health and Human Services Belmont Report.
o Please provide the primers sequences.
o Cells: please indicate the commercial origin of the cells
o Animals: Please indicate the housing and husbandry conditions.
- Author contributions: CRediT has replaced the traditional author contributions section because it offers a systematic machine-readable author contributions format that allows for more effective research assessment. Please remove the Authors Contributions from the manuscript and use the free text boxes beneath each contributing author's name in our system to add specific details on the author's contribution. More information is available in our guide to authors.
- Acknowledgements and conflict of interest should be placed after the Material and Methods.
- Acknowledgements: please add funding: 2018-05795 in the acknowledgements.
- Conflict of interest: please update the title of this section to "Disclosure statement and competing interests"

2/ Figures
- Please provide exact p values (including for ns, non significant) in the figures or their legends.
- You currently have 8 EV figures, however we can accommodate a maximum of 5 EV figures. EV Figures should be cited as 'Figure EV1, Figure EV2' etc... in the text and their respective legends should be included in the main text after the legends of regular figures. The remaining figures should be bundled together with their legends in a single PDF file called *Appendix*, which should start with a short Table of Content. Appendix figures should be referred to in the main text as: "Appendix Figure S1, Appendix Figure S2" etc. Please also clarify what are Data EV1 and Data EV2.
- Figure EV5B: "Cross strain immune fluorescence" written across the lower panels, please remove.

3/ At EMBO Press we ask authors to provide source data for the main and EV figures. Our source data coordinator will contact you to discuss which figure panels we would need source data for and will also provide you with helpful tips on how to upload and organize the files.
Numerical data should be provided as individual .xls or .csv files (including a tab describing the data). For blots or microscopy, uncropped images should be submitted (using a zip archive if multiple images need to be supplied for one panel). Additional information on source data and instruction on how to label the files are available at
.

4/ Please provide "The paper explained": EMBO Molecular Medicine articles are accompanied by a summary of the articles to

emphasize the major findings in the paper and their medical implications for the non-specialist reader. Please provide a draft summary of your article highlighting
- the medical issue you are addressing,
- the results obtained and
- their clinical impact.
This may be edited to ensure that readers understand the significance and context of the research. Please refer to any of our published articles for an example.

5/ Every published paper now includes a 'Synopsis' to further enhance discoverability. Synopses are displayed on the journal webpage and are freely accessible to all readers. They include a short stand first (maximum of 300 characters, including space) as well as 2-5 one-sentences bullet points that summarizes the paper. Please write the bullet points to summarize the key NEW findings. They should be designed to be complementary to the abstract - i.e. not repeat the same text. We encourage inclusion of key acronyms and quantitative information (maximum of 30 words / bullet point). Please use the passive voice. Please attach these in a separate file or send them by email, we will incorporate them accordingly.

Please also suggest a striking image or visual abstract to illustrate your article as a PNG file 550 px wide x 300-600 px high.

6/ As part of the EMBO Publications transparent editorial process initiative (see our Editorial at http://embomolmed.embopress.org/content/2/9/329), EMBO Molecular Medicine will publish online a Review Process File (RPF) to accompany accepted manuscripts.
This file will be published in conjunction with your paper and will include the anonymous referee reports, your point-by-point response and all pertinent correspondence relating to the manuscript. Let us know whether you agree with the publication of the RPF and as here, if you want to remove or not any figures from it prior to publication.
Please note that the Authors checklist will be published at the end of the RPF.

I look forward to receiving your revised manuscript.

Yours sincerely,

Lise Roth

Lise Roth, PhD
Senior Editor
EMBO Molecular Medicine

***** Reviewer's comments *****

Referee #2 (Remarks for Author):

I feel as though the authors have responded adequately to my comments.

Referee #3 (Remarks for Author):

The response from you is very impressive with all the details. I agree with you in revising the title of this manuscript.

On clarifying the IgG isotype being IgG1, I have a suggestion for future direction for the mAb25. Perhaps you may have thought about it. Re-engineer this mAb to different classes of Fc, IgG2, 3, 4 etc and determine the effector functions. We may learn more on the protection/efficacy of this unique antibody. All the best.

The authors addressed the editorial issues.

9th Nov 2022

Dear Dr. Nordenfelt,

Thank you for providing your revised files. There are a few minor issues that still need to be addressed before I can proceed with acceptance of your manuscript:

1/ Manuscript text:
- Material and methods, Human samples: Please include the full statement that the experiments conformed to the principles set out in the WMA Declaration of Helsinki and the Department of Health and Human Services Belmont Report.
- Data availability: Thank you for depositing the NGS data. Please remove "Bacterial amino acid translation of genomic DNA sequences is available in Appendix Supplementary Data 1." from this section.

2/ Figures:
- Please provide exact p values, not a range (including for ns, non significant) in the figures or their legends. Some people found that to keep the figures clear, providing an appendix supplemental table with all exact p-values was preferable. You are welcome to do this if you want to.

3/ Checklist:
- Please indicate in which section of the manuscript the information is available for all sections of the checklist.

5/ Synopsis:
- Thank you for providing a nice synopsis picture. Please resize it as a PNG/JPEG/TIFF file 550 px wide x 300-600 px high, and make sure that the text remains legible.
- The synopsis text should be shortened to include a brief stand first (maximum of 300 characters, including space) as well as 2-5 one-sentences bullet points that summarizes the paper (maximum of 30 words / bullet point). Please write the bullet points to summarize the key NEW findings. They should be designed to be complementary to the abstract - i.e. not repeat the same text.

Thank you for bearing with these last minor issues. I look forward to receiving your revised manuscript.

With kind regards,

Lise Roth

Lise Roth, PhD
Senior Editor
EMBO Molecular Medicine

.

The authors addressed the remaining editorial issues.

16th Nov 2022

Dear Dr. Nordenfelt,

Thank you for submitting your revised files. I am pleased to inform you that your manuscript is accepted for publication and is now being sent to our publisher to be included in the next available issue of EMBO Molecular Medicine.

We would like to remind you that as part of the EMBO Publications transparent editorial process initiative, EMBO Molecular Medicine will publish a Review Process File online to accompany accepted manuscripts. If you do NOT want the file to be published or would like to exclude figures, please immediately inform the editorial office via e-mail.

Please read below for additional IMPORTANT information regarding your article, its publication and the production process.

Congratulations on your interesting work!

With kind regards,

Lise Roth

Lise Roth, Ph.D
Senior Editor
EMBO Molecular Medicine

Follow us on Twitter @EmboMolMed
Sign up for eTOCs at embopress.org/alertsfeeds

EMBO Press Author Checklist

Corresponding Author Name: Nordenfelt	
Journal Submitted to: EMBO Molecular Medicine	
Manuscript Number: EMM-2022-16208-V2	

USEFUL LINKS FOR COMPLETING THIS FORM
The EMBO Journal - Author Guidelines
EMBO Reports - Author Guidelines
Molecular Systems Biology - Author Guidelines
EMBO Molecular Medicine - Author Guidelines

Reporting Checklist for Life Science Articles (updated January 2022)
This checklist is adapted from Materials Design Analysis Reporting (MDAR) Checklist for Authors. MDAR establishes a minimum set of requirements in transparent reporting in the life sciences (see Statement of Task: 10.31222/osf.io/9sm4x). Please follow the journal's guidelines in preparing your manuscript.
Please note that a copy of this checklist will be published alongside your article.

Abridged guidelines for figures
1. Data
The data shown in figures should satisfy the following conditions:
- the data were obtained and processed according to the field's best practice and are presented to reflect the results of the experiments in an accurate and unbiased manner.
- ideally, figure panels should include only measurements that are directly comparable to each other and obtained with the same assay.
- plots include clearly labeled error bars for independent experiments and sample sizes. Unless justified, error bars should not be shown for technical replicates.
- if n<5, the individual data points from each experiment should be plotted. Any statistical test employed should be justified.
- Source Data should be included to report the data underlying figures according to the guidelines set out in the authorship guidelines on Data Presentation.

2. Captions
Each figure caption should contain the following information, for each panel where they are relevant:
- a specification of the experimental system investigated (eg cell line, species name).
- the assay(s) and method(s) used to carry out the reported observations and measurements.
- an explicit mention of the biological and chemical entity(ies) that are being measured.
- an explicit mention of the biological and chemical entity(ies) that are altered/varied/perturbed in a controlled manner.
- the exact sample size (n) for each experimental group/condition, given as a number, not a range;
- a description of the sample collection allowing the reader to understand whether the samples represent technical or biological replicates (including how many animals, litters, cultures, etc.).
- a statement of how many times the experiment shown was independently replicated in the laboratory.
- definitions of statistical methods and measures:
  - common tests, such as t-test (please specify whether paired vs. unpaired), simple χ2 tests, Wilcoxon and Mann-Whitney tests, can be unambiguously identified by name only, but more complex techniques should be described in the methods section;
  - are tests one-sided or two-sided?
  - are there adjustments for multiple comparisons?
  - exact statistical test results, e.g., P values = x but not P values < x;
  - definition of 'center values' as median or average;
  - definition of error bars as s.d. or s.e.m.

Please complete ALL of the questions below.
Select "Not Applicable" only when the requested information is not relevant for your study.

Materials

Newly Created Materials	Information included in the manuscript?	In which section is the information available? (Reagents and Tools Table, Materials and Methods, Figures, Data Availability Section)
New materials and reagents need to be available; do any restrictions apply?	Yes	Data Availability Section

Antibodies	Information included in the manuscript?	In which section is the information available? (Reagents and Tools Table, Materials and Methods, Figures, Data Availability Section)
For **antibodies** provide the following information:<br>- Commercial antibodies: RRID (if possible) or supplier name, catalogue number and or/clone number<br>- Non-commercial: RRID or citation	Not Applicable	

DNA and RNA sequences	Information included in the manuscript?	In which section is the information available? (Reagents and Tools Table, Materials and Methods, Figures, Data Availability Section)
**Short novel DNA or RNA including primers, probes**: provide the sequences.	Not Applicable	

Cell materials	Information included in the manuscript?	In which section is the information available? (Reagents and Tools Table, Materials and Methods, Figures, Data Availability Section)
**Cell lines:** Provide species information, strain. Provide accession number in repository **OR** supplier name, catalog number, clone number, and/**OR** RRID.	Yes	Materials and Methods
**Primary cultures:** Provide species, strain, sex of origin, genetic modification status.	Yes	Materials and methods
Report if the cell lines were recently **authenticated** (e.g., by STR profiling) and tested for mycoplasma contamination.	Yes	Materials and Methods

Experimental animals	Information included in the manuscript?	In which section is the information available? (Reagents and Tools Table, Materials and Methods, Figures, Data Availability Section)
**Laboratory animals or Model organisms:** Provide species, strain, sex, age, genetic modification status. Provide accession number in repository **OR** supplier name, catalog number, clone number, **OR** RRID.	Yes	Materials and methods
**Animal observed in or captured from the field:** Provide species, sex, and age where possible.	Not Applicable	
Please detail **housing and husbandry conditions**.	Not Applicable	

Plants and microbes	Information included in the manuscript?	In which section is the information available? (Reagents and Tools Table, Materials and Methods, Figures, Data Availability Section)
**Plants:** provide species and strain, ecotype and cultivar where relevant, unique accession number if available, and source (including location for collected wild specimens).	Not Applicable	
**Microbes:** provide species and strain, unique accession number if available, and source.	Yes	Materials and methods

Human research participants	Information included in the manuscript?	In which section is the information available? (Reagents and Tools Table, Materials and Methods, Figures, Data Availability Section)
If collected and within the bounds of privacy constraints report on age, sex and gender or ethnicity for all study participants.	Yes	Materials and methods

Core facilities	Information included in the manuscript?	In which section is the information available? (Reagents and Tools Table, Materials and Methods, Figures, Data Availability Section)
If your work benefited from core facilities, was their service mentioned in the acknowledgments section?	Yes	Acknowledgements

Design

Study protocol	Information included in the manuscript?	In which section is the information available? (Reagents and Tools Table, Materials and Methods, Figures, Data Availability Section)
If study protocol has been **pre-registered, provide DOI in the manuscript**. For clinical trials, provide the trial registration number **OR** cite DOI.	Not Applicable	
Report the **clinical trial registration number** (at ClinicalTrials.gov or equivalent), where applicable.	Not Applicable	

Laboratory protocol	Information included in the manuscript?	In which section is the information available? (Reagents and Tools Table, Materials and Methods, Figures, Data Availability Section)
Provide DOI OR other citation details if **external detailed step-by-step protocols** are available.	Not Applicable	

Experimental study design and statistics	Information included in the manuscript?	In which section is the information available? (Reagents and Tools Table, Materials and Methods, Figures, Data Availability Section)
Include a statement about **sample size** estimate even if no statistical methods were used.	Yes	Materials and methods, figure legends
Were any steps taken to minimize the effects of subjective bias when allocating animals/samples to treatment (e.g. **randomization procedure**)? If yes, have they been described?	Yes	materials and methods
Include a statement about **blinding** even if no blinding was done.	Yes	materials and methods
Describe **inclusion/exclusion criteria** if samples or animals were excluded from the analysis. Were the criteria pre-established?<br><br>If sample or data points were omitted from analysis, report if this was due to attrition or intentional exclusion and provide justification.	Yes	materials and methods
For every figure, are **statistical tests** justified as appropriate? Do the data meet the assumptions of the tests (e.g., normal distribution)? Describe any methods used to assess it. Is there an estimate of variation within each group of data? Is the variance similar between the groups that are being statistically compared?	Yes	Materials and methods, figure legends

Sample definition and in-laboratory replication	Information included in the manuscript?	In which section is the information available? (Reagents and Tools Table, Materials and Methods, Figures, Data Availability Section)
In the figure legends: state number of times the experiment was **replicated** in laboratory.	Yes	Figure legends
In the figure legends: define whether data describe **technical or biological replicates**.	Yes	Figure legends

Ethics

Ethics	Information included in the manuscript?	In which section is the information available? (Reagents and Tools Table, Materials and Methods, Figures, Data Availability Section)
Studies involving **human participants**: State details of **authority granting ethics approval** (IRB or equivalent committee(s), provide reference number for approval.	Yes	Materials and methods
Studies involving **human participants**: Include a statement confirming that **informed consent** was obtained from all subjects and that the experiments conformed to the principles set out in the WMA Declaration of Helsinki and the Department of Health and Human Services Belmont Report.	Yes	Materials and methods
Studies involving **human participants:** For publication of **patient photos**, include a statement confirming that consent to publish was obtained.	Not Applicable	
Studies involving experimental **animals**: State details of **authority granting ethics approval** (IRB or equivalent committee(s), provide reference number for approval. Include a statement of compliance with ethical regulations.	Yes	Materials and methods
Studies involving **specimen and field samples:** State if relevant **permits** obtained, provide details of authority approving study; if none were required, explain why.	Not Applicable	

Dual Use Research of Concern (DURC)	Information included in the manuscript?	In which section is the information available? (Reagents and Tools Table, Materials and Methods, Figures, Data Availability Section)
Could your study fall under dual use research restrictions? Please check biosecurity documents and list of **select agents and toxins** (CDC): https://www.selectagents.gov/sat/list.htm	Not Applicable	
If you used a select agent, is the security level of the lab appropriate and reported in the manuscript?	Not Applicable	
If a study is subject to dual use research of concern regulations, is the name of the **authority granting approval and reference number** for the regulatory approval provided in the manuscript?	Not Applicable	

Reporting

The MDAR framework recommends adoption of discipline-specific guidelines, established and endorsed through community initiatives. Journals have their own policy about requiring specific guidelines and recommendations to complement MDAR.

Adherence to community standards	Information included in the manuscript?	In which section is the information available? (Reagents and Tools Table, Materials and Methods, Figures, Data Availability Section)
State if relevant guidelines or checklists (e.g., **ICMJE, MIBBI, ARRIVE, PRISMA**) have been followed or provided.	Not Applicable	
For **tumor marker prognostic studies,** we recommend that you follow the **REMARK** reporting guidelines (see link list at top right). See author guidelines, under 'Reporting Guidelines'. Please confirm you have followed these guidelines.	Not Applicable	
For **phase II and III randomized controlled trials**, please refer to the **CONSORT** flow diagram (see link list at top right) and submit the CONSORT checklist (see link list at top right) with your submission. See author guidelines, under 'Reporting Guidelines'. Please confirm you have submitted this list.	Not Applicable	

Data Availability

Data availability	Information included in the manuscript?	In which section is the information available? (Reagents and Tools Table, Materials and Methods, Figures, Data Availability Section)
Have **primary datasets** been deposited according to the journal's guidelines (see 'Data Deposition' section) and the respective accession numbers provided in the Data Availability Section?	Yes	Data availability
Were **human clinical and genomic datasets** deposited in a public access-controlled repository in accordance to ethical obligations to the patients and to the applicable consent agreement?	Not Applicable	
Are **computational models** that are central and integral to a study available without restrictions in a machine-readable form? Were the relevant accession numbers or links provided?	Not Applicable	
If publicly available data were reused, provide the respective **data citations in the reference list**.	Not Applicable	
